# First Evidence-Based Guideline for Interventions in FASD

**DOI:** 10.1055/a-2547-4610

**Published:** 2025-03-19

**Authors:** Sonja Strieker, Florian Heinen, Annika Ziegler, Christine Schmucker, Ina Kopp, Mirjam N. Landgraf

**Affiliations:** 1 Department of Paediatric Neurology and Developmental Medicine, iSPZ Hauner MUC, Dr. von Hauner Children's Hospital, Ludwig-Maximilians-University, Munich, Germany; 2 Institute for Evidence in Medicine, Faculty of Medicine and Medical Centre, University of Freiburg, Germany; 3 Association of the Scientific Medical Societies in Germany, Berlin, Germany

**Keywords:** fetal alcohol spectrum disorder, fetal alcohol syndrome, alcohol-related neurodevelopmental disorder

## Abstract

**Background:**

Prenatal alcohol exposure causes disruptions in brain development. The resulting disorder, fetal alcohol spectrum disorder (FASD), cannot be cured, but interventions can help improve the daily functioning of affected children and adolescents and the quality of life for the entire family.

**Objective:**

The aim of the German guideline version 2024 is to provide validated and evidence-based recommendations on interventions for children and adolescents with FASD.

**Methods:**

We searched for international guidelines and performed a systematic literature review and a hand search to identify literature (published 2012–2022) on interventions for children (0–18 years) with FASD. The quality of the literature was assessed for predefined outcomes using the GRADE method (grading of recommendations, assessment, development, and evaluation). We established a multidisciplinary guideline group, consisting of 15 professional societies, a patient support group, and 10 additional experts in the field. The group agreed on recommendations for interventions based on the systematic review of the literature and formulated additional recommendations, based on clinical experience/expert evidence in a formal consensus process.

**Results:**

No international guideline focusing on interventions for patients with FASD was found. Thirty-two publications (4 systematic reviews and 28 original articles) were evaluated. The analysis resulted in 21 evidence-based recommendations and 26 expert consensus, covering the following topics: neuropsychological functioning, adverse effects of therapy, complications/secondary conditions, quality of life, caregiver burden, knowledge of FASD, and coping and self-efficacy.

**Conclusion:**

The German guideline is the first internationally to provide evidence-based recommendations for interventions in children and adolescents with FASD.

## Introduction

Fetal alcohol spectrum disorder (FASD) includes a range of conditions resulting from prenatal alcohol exposure (PAE) during pregnancy. Maternal alcohol consumption during pregnancy carries the risk of affecting fetal development, leading to lifelong physical, behavioral, and cognitive impairments. FASD serves as an umbrella term for three primary clinical entities: fetal alcohol syndrome (FAS), partial fetal alcohol syndrome (pFAS), and alcohol-related neurodevelopmental disorder (ARND), each manifesting with varying severity levels. However, there is ongoing debate regarding the classification of FASD subtypes, with some definitions also including alcohol-related birth defects (ARBD) and neurobehavioral disorders associated with prenatal alcohol exposure (ND-PAE) as described in the DSM-5. 1

With an estimated incidence of approximately 1.77% 2 of live births in Germany, FASD stands as one of the most prevalent chronic conditions present at birth.

International guidelines have predominantly focused on evidence-based diagnostic criteria to facilitate early and precise identification of the disorder, 3 4 5 6 laying the groundwork for ongoing care and support for children and adolescents with FASD and their families.

Tailored interventions (measures designed to support the child's development and well-being) and support services addressing the specific needs of children and adolescents with FASD can mitigate the occurrence of secondary conditions and comorbidities of this disease, thus enhancing the quality of life for affected individuals and their social environment.

However, international guidelines seldom provide specific recommendations for the care of individuals with FASD. Beyond diagnosis, guidelines primarily offer general advice for managing individuals with FASD, such as employing clear and simple language, maintaining routines, and structuring daily activities. 7 Various guidelines advocate for the use of management plans 7 8 9 and connecting individuals with FASD and their families to resources that may improve outcomes. 7 8 9 10 11 12 13 Emphasis is also placed on the importance of educating both patients and their entire social environment about the condition. 7 11 12 Policy-level guidelines focus on establishing basic infrastructures for improved care of individuals with FASD. 14 While some specific suggestions on interventions are based on expert and patient opinions or findings from focus groups, 9 11 12 13 these studies provide valuable insights into the lived experiences and practical needs of individuals with FASD and their caregivers. Despite these efforts, there remains a critical gap in evidence-based intervention recommendations aimed at enhancing specific functions in individuals with FASD. The combination of qualitative findings and evidence-based research is essential for developing comprehensive, patient-centered care approaches. Therefore, addressing the gap in evidence-based recommendations is essential to improving outcomes for this population.

The German guideline presented here marks a significant advancement as the first internationally to provide evidence-based recommendations for interventions in children and adolescents with FASD. This represents a fundamental step toward improving the health and well-being of children with FASD.

## Methods

We tried to reduce potential bias in the guideline by ensuring a balanced composition of the guideline group, which was established in 2022 and consisted of representatives from 15 German professional societies, 10 FASD experts, and two members of the patient support group “FASD Deutschland.” Additionally, two nonvoting observers from the German Ministry of Health (Manuela Schumann, Kirsten Reinhard MD) participated in the guideline conferences and the consensus process was overseen by methodological supervisors and moderators ( Supplementary Table S1 , available in the online version).

Each member of the consensus group provided a declaration of interest according to international requirements, 15 which was reviewed by an independent person (conflict of interest officer). These declarations were discussed at the inaugural guideline conference (July 1, 2022). None of the consensus group members had conflicts of interest that warranted exclusion from the voting process or any related activities.

The project on which this publication is based was funded by the Innovation Fund of the Federal Joint Committee (Gemeinsamer Bundesausschuss—G-BA, funding code 01VSF21012). The funding did not influence the development and content of the guideline in any way.

The key question for the systematic literature search was consented to in the first consensus conference and structured in PICOS format (PICOS: population, intervention, comparator, outcome, and study design). The relevance of each outcome was rated on a 1 to 9 scale (1–3: limited importance; 4–6: important but not critical; 7–9: critical). The PICOS scheme, upon which the inclusion criteria for the systematic literature review were based, is detailed in Supplementary Table S2 (available in the online version).

The key question was:

Which interventions (I) are associated with positive outcome criteria (O) compared to no interventions, placebos, contextual effects, alternative interventions, or pre–post comparisons (C) in children and adolescents (0–18 years) with FASD (P)?

The “positive outcome criteria” were further specified into the following domains:

- Improvement in the neuropsychological functions of children/adolescents with FASD
- Avoidance of adverse effects of the interventions
- Reduction of complications/secondary diseases
- Improving the participation of children/young people with FASD
- Improving the quality of life of children/young people with FASD
- Relief for caregivers (biological, foster, and adoptive parents, other caregivers) and improving the quality of life of the entire family/institution
- Enhancing knowledge of the health condition or disability and fostering insight into the associated challenges

The outcomes selected for this guideline address the multifaceted needs of children with FASD and their support systems. Improvement of neuropsychological functions was prioritized due to its alignment with the German S3 guideline on FASD diagnostics, reflecting its centrality to cognitive, emotional, and social development. Other outcomes, such as avoiding side effects, complications, and secondary conditions, highlight the importance of safe and preventive care. Enhancing participation, quality of life, and caregiver support aligns with person-centered approaches, acknowledging the critical role of families and social integration in successful interventions. Finally, knowledge dissemination and caregiver empowerment were included to address gaps in awareness and promote sustainable care practices. Together, these outcomes offer a comprehensive framework for improving both individual and systemic care for children and adolescents with FASD.

Based on our key question, we conducted a systematic literature search in the databases Medline via PubMed, Wiley Online Library via Cochrane Library, EBSCO (PsycINFO, PsycARTICLES, PSYNDEX), and Epistemonikos, covering English and German literature published between January 1, 2012, and August 9, 2022. Supplementary Tables S2 and S3 (available in the online version) present the search strategy and inclusion and exclusion criteria used to identify eligible publications, respectively.

The quality of evidence for outcomes was assessed using the GRADE method (grading of recommendations, assessment, development, and evaluation). First, we evaluated the risk of bias of each publication individually using RoB 2 (Cochrane risk-of-bias tool—second version 16 ) for randomized controlled trials, ROBINS-I (Tool for assessing risk of bias in nonrandomized studies of interventions 17 ) for nonrandomized controlled trials, a modified version of ROBINS-I instrument for noncontrolled studies, and AMSTAR-2 instrument (a measurement tool to assess systematic reviews—second version 18 ) for systematic reviews. Afterward, we assessed the quality of evidence for each predefined outcome using the GRADE criteria (risk of bias/study limitations, indirectness, inconsistency of results, imprecision, publication bias, effect size, dose-response gradient, and the influence of residual and plausible confounders). The quality of evidence was categorized into four levels: very low, low, moderate, and high.

Based on the evidence found in the literature, recommendations were formulated according to the requirements of the Association of the Scientific Medical Societies in Germany (AWMF): recommendations with the highest level A are expressed as “should,” followed by level B “ought to,” and the lowest level 0 “may be considered.”

In cases where insufficient evidence was available to make evidence-based recommendations, expert consensus was sought. This process involved gathering insights and opinions from professionals with extensive experience in the field. Experts were asked to provide their perspectives on relevant interventions and practices, ensuring that recommendations were still grounded in practical expertise and current clinical experience. Expert consensus allowed us to address areas with limited or no empirical data, ensuring comprehensive guidance for practitioners despite the lack of robust evidence. These expert consensus were formulated according to the evidence-based recommendations.

The recommendations and expert consensus for interventions in children and adolescents with FASD were discussed and modified by the guideline group in the third (March 31, 2023), and fourth (June 7, 2023) online consensus conference, considering the evidence, clinical relevance, practical applicability, risk-benefit assessments, and ethical considerations. Guided by an independent methodologically experienced moderator, the resulting recommendations and expert consensus were consented upon through a formal consensus process, utilizing the Nominal Group Technique. 19 For reaching “consensus” an agreement of > 75% of the participating guideline group members was required. “strong consensus” represents an agreement of > 95%.

## Results

**Fig. 1:**
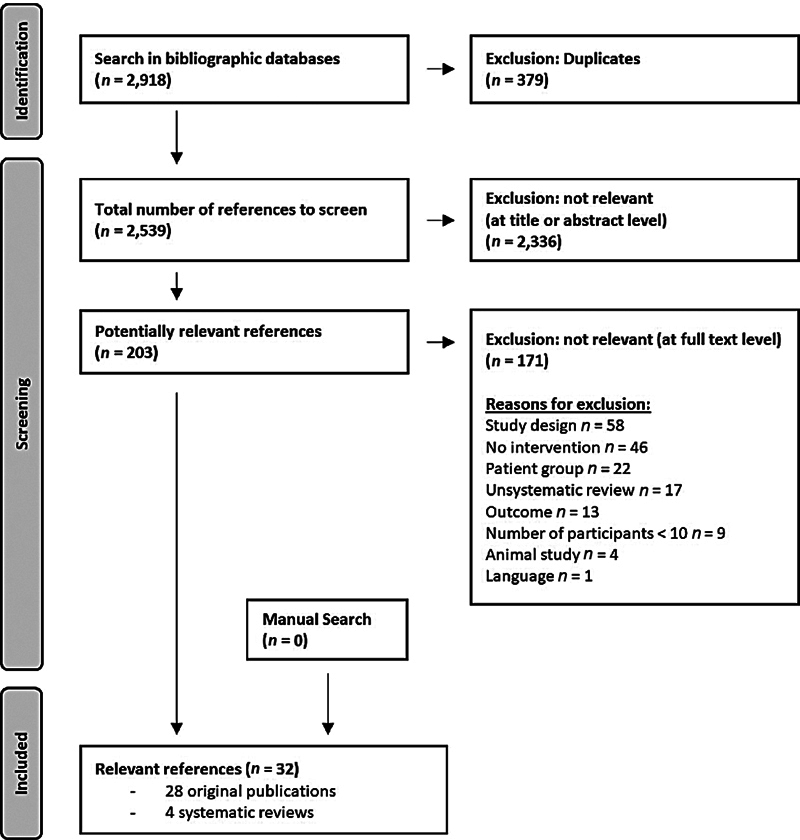
Flowchart of the systematic literature search.

We identified a total of 2,539 publications after deduplication. We did not find any international guidelines for interventions in children or adolescents with FASD. After title/abstract screening and full-text screening we included 32 publications (including four systematic reviews) for quality assessment ( Fig. 1 ). To access the complete list of publications included in the analyses, the risk of bias assessment, and the summary of findings tables (GRADE), please refer to Supplementary Documents S1–S3 (available in the online version).

The guideline group agreed on 21 evidence-based recommendations and 26 expert consensus. In the following, all recommendations and expert consensus are listed by outcomes. In compliance with AWMF regulations, the exact wording of the consented recommendations and expert consensus statements has been faithfully translated from German into English. This ensures that neither the content nor the phrasing is altered, maintaining the intended meaning and recommendation strength.

Expert consensus statements are marked with EC.

For each recommendation, we provide the following information in parentheses:

1) Evidence grading (EG) based on the GRADE methodology, classified as:
  - Low
  - Moderate
  - High
2) Recommendation grading (RG), categorized as:
  - A = strong recommendation (“should”)
  - B = moderate recommendation (“ought to”)
  - 0 = open recommendation (“may be considered”)
3) The corresponding reference source

Additionally, background information is included where necessary.

Disclaimer: All interventions need to consider the individual circumstances of the person being treated as well as their social environment and their financial situation.

### Improvement in the Neuropsychological Functions of Children/Adolescents with FASD

We divided this outcome into sub-outcomes according to the German guideline that identified specific functions of the central nervous system that are often impaired in individuals with FASD and, therefore, part of the diagnostic criteria. 20 21

Table 1 shows the recommendations and expert consensus for the improvement of the neuropsychological functions, divided into ten FASD-relevant CNS domains.

**Table 1:** Recommendations for improving the neuropsychological functions of children/adolescents with FASD

Sub-outcome	Recommendation/expert consensus
Cognitive performance/intelligence	• Children and adolescents with FASD and intellectual disability should not be excluded from guideline-based therapies (guideline “intellectual disability”) a (EC)
Development	• Infants, toddlers, and primary school children with FASD should undergo developmental assessments at regular intervals so that developmental impairments can be diagnosed at an early stage and appropriate support measures can be initiated (EC)
Epilepsy	• In children with FASD and epilepsy, drug and nondrug therapies to reduce seizure symptoms should be based on the usual therapeutic measures and the guideline “diagnostic principles for childhood epilepsy” a (EC).
Language	• For children with FASD, interventions to improve language development should be based on the guideline “therapy of language development disorders” a (EC) • Regarding therapy, an interdisciplinary decision (including developmental diagnostics, speech pedagogy/logopedics, and psychology) ought to be made to ensure individually adapted support (EC)
Fine-/graphomotoric skills or gross motor coordination	• Interventions to improve coordination disorders in children with FASD should be based on the guideline “circumscribed developmental disorders of motor functions” a (EC) • The support ought to be adapted to the child's neurological and neurocognitive impairments and, due to the common difficulty of transferring learned content, ought to be closely aligned with everyday life (EC)
Spatial-visual perception or spatial-constructive abilities	• Children with FASD and visual-spatial dysfunctions, visual impairment should be clinically ruled out by an ophthalmologist. If a visual impairment is present, appropriate aids (e.g., glasses, eye covering) should be prescribed and, depending on the clinical symptoms, visual support should be initiated (EC) • It may be considered to offer individually adapted occupational therapy to the child and practical exercise instructions to caregivers in order to improve the visual-spatial functions of children with FASD (EC)
Executive functions	• Transcranial direct current stimulation (tDCS) ought not to be used solely to improve executive functions in children with FASD (EG: high; RG: B 39 ) • Training aimed at promoting inhibitory control, emotion regulation, and behavior regulation, combined with parent training, ought to be used to enhance executive functions in school-aged children with FASD (EG: moderate; RG: B 28 47 49 )
Mathematical skills	• Training to develop arithmetic thinking and skills ought to be used to improve arithmetic abilities in preschool- and school-aged children with FASD. The training should be adapted to FASD and the child's developmental stage (EG: high; RG: B 29 45 50 51 ).
Learning and memory skills	• TDCS ought not to be used solely to improve learning and memory in children with FASD (EG: high; RG: B 39 ).
Attention	• Drug therapy recommendations to improve attention in children and adolescents with FASD and ADHD should be based on the guideline “ADHD in children, adolescents and adults” a (EC) • TDCS ought not to be used solely to improve attention in children with FASD (EG: high; RG: B 39 ) • It may be considered using extrinsic reinforcement to support children with FASD in certain areas of attention (EG: low; RG: 0 37 ) • Neurocognitive interventions focusing on self-control and/or attention control strategies ought to be offered to improve attention performance in preschool- and school-aged children with FASD (EG: moderate; RG: B 30 31 40 50 51 ) • It may be considered using parent training in addition to neurocognitive training of the children in order to increase the therapeutic effect on the children's attention performance (EG: moderate; RG: 0 31 40 )
Social skills and behavior	• Children with FASD ought to receive social skills training tailored to FASD to increase their knowledge of appropriate social behavior and improve their social skills (EG: moderate; RG: B 41 50 51 52 ) • Neurocognitive training focusing on the development of regulation strategies should be used to improve the behavioral and emotional regulation in children with FASD (EG: high; RG: A 28 30 31 38 40 42 43 47 ) • In addition to neurobehavioral and neurocognitive training of children with FASD to improve emotional and behavioral regulation, a therapy attempt with neuroleptics can be considered for severe behavioral disorders. This is an off-label use for most active substances (EC) • Children/adolescents (≥6 years of age) with FASD and ADHD should be offered therapy with methylphenidate to improve hyperactivity and impulsivity (EG: high; RG: A 35 50 53 ) • Social skills training ought to be supplemented by psychoeducation of parents/caregivers (EG: moderate; RG: B 41 50 51 52 ) • Neurocognitive training ought to be supplemented by resource-oriented psychoeducation of parents/caregivers in order to further improve the children's regulation strategies (EG: high; RG: B 31 38 40 42 43 47 ) • Psychoeducational measures should be offered to parents/caregivers of children with FASD to encourage positive behavioral change in the children (EG: moderate; RG: A 48 ) • When providing psychoeducation to parents, their cognitive abilities and any existing neurological and psychiatric disorders (including FASD) should be considered (EC)
Additional recommendations/expert consensus	• Children with FASD should receive pedagogical support tailored to their individual abilities (cognitive abilities, executive functions, social-adaptive abilities, and behavioral regulation) in kindergarten and school (EC) • All educators and teachers should receive information regarding fetal alcohol spectrum disorders and strategies adapted to the clinical profile when teaching and interacting with children and adolescents with FASD (EC) • A support and treatment plan that is tailored to the specific needs of the child or adolescent with FASD should be developed, formulated, and implemented. This process should involve a collaboration of the legal guardians/parents, FASD professionals (e.g., doctors or psychologists providing care), and educators/teachers. Additionally, a potential compensation for disadvantages should be considered (EC)

Abbreviations: EC, expert consensus; EG, evidence grading (low; moderate; high); FASD, fetal alcohol spectrum disorder; RG, recommendation grading (A—strong recommendation; B—recommendation; 0—open recommendation).

a The guideline is available only in German.

#### Background

##### Attention

There is evidence that high choline intake may improve attention in children with FASD. In order to prevent adverse effects of choline supplementations (e.g., fishy body odor) a choline-rich diet can be used to ensure a sufficient supply of choline for the child.

Before starting drug treatments, all relevant factors (e.g., age, severity of problems, comorbidities, individual needs) must be considered. The treatment must be discussed with the children/adolescents with FASD (if old enough) and their parents/legal guardians and they must be informed about possible adverse effects. Further, regular monitoring of possible adverse effects and the effectiveness of the treatment must be performed and modified if needed.

Neurocognitive training includes neurobehavioral, cognitive, and behavioral therapies, serious games as well as similar therapeutic modalities targeting domains, such as attention, memory, problem-solving, spatial reasoning, language, interaction, and executive functions. It aims at strengthening neural connections, facilitating the formation of new synapses, and enhancing existing neural networks.

Social skills training is a therapeutic approach focused on improving individuals' ability to interact effectively in social situations. It involves teaching specific social behaviors, communication skills, and interpersonal strategies to enhance social competence, confidence, and relationships.

### Avoidance of Adverse Effects of the Interventions

In the field of preventing side effects of interventions, two expert consensuses have been adopted ( Table 2 ).

**Table 2:** Recommendations for avoiding adverse effects of interventions in children/adolescents with FASD

Recommendation/expert consensus
• Due to potential adverse drug reactions drug therapies ought to be administered to children and adolescents with FASD if pedagogical-psychological treatments (e.g., neurocognitive training) are not sufficiently effective in reducing the CNS functional impairments (EC) • Drug therapies should be provided under strict medical supervision. When selecting and monitoring drug therapies, the recommendations of the guidelines “ADHD in children, adolescents and adults” a and “disorder of social behavior” a should be followed, along with the specialist information on the medication (EC)

Abbreviations: EC, expert consensus; EG, evidence grading (low; moderate; high); FASD, fetal alcohol spectrum disorder; FASD, fetal alcohol spectrum disorder; RG, recommendation grading (A—strong recommendation; B—recommendation; 0—open recommendation).

a The guideline is available only in German.

#### Background

When offering pharmaceutical therapies, FASD as well as the individual needs and comorbidities must be considered as well as possible interactions with other medications.

### Reduction of Complications/Secondary Diseases

The evidence-based recommendations and expert consensuses defined in the guideline for the outcome “reduction of complications/secondary disorders” are presented in Tables 3 and 4 .

**Table 3:** Recommendations for reducing complications/secondary diseases in children/adolescents with FASD (part 1)

Recommendation/expert consensus
• To prevent secondary diseases or complications, or at least detect them at an early stage, children and adolescents with FASD should undergo regular pediatric and developmental diagnostic examinations throughout their entire age range, from 0–18 years (EC) • Child and adolescent psychiatry should be promptly involved if there are any indications of psychiatric symptoms or risky behavior (e.g., risky alcohol/drug use, self/other endangerment, suicidal acts) in the child/adolescent (EC) • Depending on the clinical symptoms, other specialties should be consulted, such as pediatric subdisciplines, ear, nose, and throat (ENT) specialists, ophthalmology, orthopedics, pediatric radiology, psychotherapy, and others (EC) • To develop effective therapies and interventions for children/adolescents with FASD, these other specialties ought to be integrated into a comprehensive therapy plan, and professional case management ought to be established for each child (EC) • Transparent, interdisciplinary cooperation and the involvement of the children and adolescents themselves, as well as their caregivers/legal guardians ought to be considered throughout the entire support system and therapy period. A stable social environment ought to be created to prevent secondary disorders (EC)

Abbreviations: EC, expert consensus; EG, evidence grading (low; moderate; high); FASD, fetal alcohol spectrum disorder; RG, recommendation grading (A—strong recommendation; B—recommendation; 0—open recommendation).

**Table 4:** Recommendations for reducing complications/secondary diseases in children/adolescents with FASD (part 2)

Sub-outcome	Recommendation/expert consensus
Risky behavior	• To reduce risky alcohol consumption in adolescents with FASD, alcohol-preventive neurocognitive training ought to be offered to adolescents, along with psychoeducation for their parents (EG: high; RG: B 44 54 ) • It may be considered to offer training to reduce risky behaviors to primary school children with FASD in order to increase their knowledge (EC) a
School failure and drop-out	• To ensure positive learning outcomes and prevent school failure or dropout, learning content and environments ought to be tailored to the impairments of children/young people with FASD. If necessary, additional support measures (at school and/or at home) ought to be introduced. Therefore, doctors/psychologists/therapists in charge ought to communicate with the educational staff at school or after-school care, as well as the children/young people, and their guardians, to coordinate educational measures and support integration into existing support programs (EC)
Delinquency	• To prevent delinquent behavior, it may be considered to use neurocognitive training or drug therapies at an early stage to support the child's regulation of emotions and behavior. Since adolescents with FASD often struggle to foresee the consequences of their actions, the consequences of delinquent behavior ought to be explained to them comprehensibly and repeatedly by various professionals as well as close caregivers. Additionally, these explanations ought to be adapted to the individual's learning style and illustrated accordingly. (EC) • The police and judiciary ought to be educated about FASD and informed about the specific characteristics of the individual child/adolescent with FASD. They ought to involve responsible medical professionals and legal guardians/caregivers in their assessment of delinquent behavior to determine the extent to which age-appropriate capacity for understanding and control is present. This will allow them to judge effectively and fairly in each case (EC)
	•
Maltreatment	• To prevent child maltreatment (as victims or offenders), children and adolescents with FASD ought to be offered early, easily understandable, and repeated education (including sexual education with contraceptive options) as well as strategies for self-assertion and support in interpersonal interactions. In addition, guardians and professionals across the entire support system ought to be informed about the vulnerability of children and adolescents with FASD to child abuse (EC)

Abbreviations: EC, expert consensus; EG, evidence grading (low; moderate; high); FASD, fetal alcohol spectrum disorder; RG, recommendation grading (A—strong recommendation; B—recommendation; 0—open recommendation).

a The training should be tailored to the child's developmental stage and aligned with their level of adaptive functioning.

#### Background

Compared to the average population individuals with FASD have higher rates of conditions such as psychiatric diseases (incl. addictions), risky behavior, school failure, delinquency, maltreatment, hospitalization, and somatic diseases (e.g., visual disturbances, dizziness, insomnia, headaches, and shortness of breath).

Building on the principles of transparent and interdisciplinary cooperation, it is also essential to incorporate a strengths-based perspective that recognizes and leverages the individual strengths of children and adolescents with FASD, as well as those of their caregivers, throughout the support system and therapy period. By emphasizing individual strengths alongside therapeutic interventions, we can promote a holistic approach that fosters self-efficacy and encourages active participation from children, adolescents, and their caregivers in the therapeutic process.

Risky behavior and delinquency: It is important to recognize that individuals with FASD may face challenges not due to a lack of understanding of rules or situations, but rather due to brain-based differences that affect impulse control, learning from experiences, and managing high-risk scenarios.

Transition: In adulthood, many individuals with FASD still require support in their daily lives. The transition from pediatricians to psychiatrists/neurologists for adults is important and should be well prepared. The guideline “Transition from Pediatrics to Adult Medicine” (only provided in German) offers general recommendations for a successful transition.

### Improving the Participation of Children/Young People with FASD

Participation was considered a highly relevant outcome by the guideline group. Based on the literature, recommendations, and expert consensus were developed for three related sub-outcomes (see Table 5 ).

**Table 5:** Recommendations for improving the participation of children/young people with FASD

Sub-outcome	Recommendation/expert consensus
Learning and application of knowledge	• If participation in learning and the application of knowledge cannot be sufficiently ensured for a child or adolescent with FASD due to individual cognitive impairments, the need for support from an integration assistant or school companion ought to be assessed, and other appropriate support measures ought to be implemented if necessary (EC) • Professional integration assistants or school companions ought to be familiar with the clinical profile of FASD and its implications for learning, planning, social behavior, and emotional regulation. They ought to be trained in working with children and adolescents with FASD, and the benefits of this support ought to be reviewed regularly (EC) • Adolescents with FASD ought to be offered educational support measures adapted to their cognitive and socio-emotional abilities as part of an individual, needs-oriented support plan coordinated with them and their legal guardians or caregivers (EC)
Domestic life	• For children with FASD, psychoeducation for parents and/or parent-child training ought to be implemented to improve participation in the home environment (EG: moderate; RG: B 40 ) • Legal guardians or caregivers of children and adolescents with FASD ought to be offered educational, psychological, and financial support tailored to the family's needs and the child's impairments to ensure stable care (EC) • If the promotion of development and education of a child/adolescent in the origin, adoptive, or foster family is not (or no longer) possible, forms of pedagogically supported, supervised living adapted to the individual needs and impairments of the child/adolescent with FASD ought to be provided. (EC)
Interpersonal interaction and relationships	• For children with FASD, neurocognitive training focusing on self-regulation or social skills ought to be implemented in combination with psychoeducation for parents to improve the child's interpersonal skills and thus participation in the lives of peers (EG: moderate; RG: B 41 47 ) • Neurocognitive therapies should be offered to children and adolescents with FASD to improve social interaction. These should be adapted to the specific impairments of children with FASD, which are biologically based due to prenatal alcohol-induced brain damage (EC) • These child-centered therapies ought to be supplemented by psychoeducation for legal guardians or caregivers and by intensive education of other caregivers (e.g., educational, therapeutic, and psychological professionals) so that they can develop an understanding of the condition and the child's individual impairments and establish strategies to improve their interactions with the child. (EC)

Abbreviations: EC, expert consensus; EG, evidence grading (low; moderate; high); FASD, fetal alcohol spectrum disorder; RG, recommendation grading (A—strong recommendation; B—recommendation; 0—open recommendation).

#### Background

Psychoeducation for individuals with FASD, their caregivers, and societal services should consider the brain-based differences of individuals with FASD, focusing on tailored strategies that account for difficulties in impulse regulation and vulnerability management. It is critical to avoid framing individuals with FASD as willfully engaging in maladaptive behaviors, and instead to highlight the importance of structured support systems that address their unique neurodevelopmental needs.

### Improving the Quality of Life of Children/Young People with FASD

Even though the improvement of quality of life through interventions was considered a highly relevant outcome by the guideline group, no literature-based evidence was found, and therefore, an expert consensus was formulated (see Table 6 ).

**Table 6:** Recommendation for improving the quality of life of children/young people with FASD

Recommendation/expert consensus
• Both in the promotion and therapy of children and adolescents with FASD, as well as in the psychoeducation and support of legal guardians and caregivers, the focus ought to be on improving or at least stabilizing the quality of life of the affected children/adolescents and their families (in addition to specific therapy goals based on the individual's impairments; EC)

Abbreviations: EC, expert consensus; EG, evidence grading (low; moderate; high); RG, recommendation grading (A—strong recommendation; B—recommendation; 0—open recommendation).

#### Background

The World Health Organization (WHO) “defines Quality of Life as an individual's perception of their position in life in the context of the culture and value systems in which they live and in relation to their goals, expectations, standards, and concerns”. 22

The systematic literature search identified no publication specifically addressing this topic.

According to subjective reports, forms of animal-assisted interventions can have a positive effect on the quality of life of children and adolescents with FASD and their families. Assistance dogs may improve the quality of life by strengthening social relationships, increasing the child's sense of security, and, thereby, achieving greater independence, as has already been documented with assistance dogs in children with autism spectrum disorders. 23 24 25 26

### Relief for Caregivers (Biological, Foster, and Adoptive Parents, Other Caregivers) and Improving the Quality of Life of the Entire Family/Institution

A guideline recommendation was agreed upon for the quality of life of the entire family, which is linked to parental stress reduction and the fulfillment of family needs ( Table 7 ).

**Table 7:** Recommendation for creating relief for caregivers (biological, foster, and adoptive parents, other caregivers) and for improving the quality of life of the entire family/institution

Recommendation/expert consensus
• Parents of children with FASD ought to be offered psychoeducation (if necessary with individual goal-setting) in combination with therapies for the child and family support to reduce parental stress and improve the fulfillment of the family's needs (EG: moderate; RG: B 42 43 55 )

Abbreviations: EC, expert consensus; EG, evidence grading (low; moderate; high); FASD, fetal alcohol spectrum disorder; RG, recommendation grading (A—strong recommendation; B—recommendation; 0—open recommendation).

#### Background

Long-term support for families adapted to the individual family factors seems necessary to fulfill their needs sustainably.

### Enhancing Knowledge of the Health Condition or Disability and Fostering Insight Into the Associated Challenges

Table 8 presents the guideline group's recommendations and expert consensus for the outcomes of “knowledge enhancement” and “disease understanding.”

**Table 8:** Recommendation for enhancing knowledge of the health condition or disability and fostering insight into the associated challenges

Recommendation/expert consensus
• Caregivers/legal guardians of children with FASD should be provided with information in group workshops in the presence of online information material or written information to improve their knowledge about the condition of FASD (EG: high; RG: A 48 ) • Legal guardians or caregivers of children with FASD ought to be offered psychoeducation in combination with therapies for the child and family support to improve their knowledge about the condition of FASD in the long term. (EG: moderate; RG: B 42 43 ) • When providing psychoeducation to caregivers/parents, we recommend considering their cognitive abilities and any neurological and psychiatric disorders (including FASD; EC) • In psychoeducation for legal guardians or caregivers, attention should be paid to their cognitive conditions and any possible neurological and psychiatric disorders (including FASD; EC) • Children and adolescents with FASD should be provided with information that is adapted to their developmental stage and cognitive abilities to improve their knowledge about the condition of FASD (EC) • According to children and adolescents with FASD and their caregivers, the knowledge and communication about their condition or the cause of their impairments often leads to relief. Therefore, research in this area should be conducted. Studies on interventions to improve awareness of the disorder in children and adolescents with FASD are lacking, but they are extremely relevant from a clinical perspective, especially in relation to risky behavior, recognition of support, help-seeking, and transition. Therefore, research projects should also be planned in this area (EC)

Abbreviations: EC, expert consensus; EG, evidence grading (low; moderate; high); FASD, fetal alcohol spectrum disorder; RG, recommendation grading (A—strong recommendation; B—recommendation; 0—open recommendation).

#### Background

Depending on the cognitive abilities of the caregivers, simple language should be used when providing information.

### Improvement in Coping and Self-Efficacy

One guideline recommendation was adopted for the improvement of coping and self-efficacy as an outcome (see Table 9 ).

**Table 9:** Recommendation for improving coping and self-efficacy in children/adolescents with FASD

Recommendation/expert consensus
• Children/adolescents with FASD and their classmates ought to be educated in school about factors of mental health and strategies for coping with health impairments to strengthen the coping skills as well as the self-concept of children/adolescents with FASD (EG: moderate; RG: B 54 )

Abbreviations: EC, expert consensus; EG, evidence grading (low; moderate; high); FASD, fetal alcohol spectrum disorder; RG, recommendation grading (A—strong recommendation; B—recommendation; 0—open recommendation).

#### Background

By sharing their personal experiences and talking about FASD, children with FASD can help peers, teachers, and parents develop a better understanding of the challenges and needs of individuals with FASD. This can reduce prejudice, increase empathy, and create an inclusive environment. Thereby, the child's needs must be respected and the voluntariness of the educational work must always be emphasized.

### Additional Expert Consensus on Quality of Life, Relief of Caregivers, Knowledge and Coping/Self-Efficacy

Since the guideline group considered the quality of life, relief for caregivers, knowledge enhancement, and coping/self-efficacy as highly relevant for the daily lives of children with FASD and their families, additional expert consensuses were adopted in these outcome areas (see Table 10 ).

**Table 10:** Additional expert consensus regarding the quality of life, relief of caregivers, knowledge, and coping/self-efficacy

Recommendation/expert consensus
• The condition of FASD, individual strengths and weaknesses, daily life organization, current issues, and planned therapy content and goals should be communicated and discussed transparently, adequately, and, if necessary, repeatedly with the children and adolescents. When planning therapy, the individual wishes, participation preferences and concerns of children and adolescents with FASD should be taken into account (EC) • Professionals who care for children and adolescents with FASD ought to be familiar with regional and national FASD self-help groups/patient advocacy organizations (EC) • The professionals ought to inform children and adolescents, their legal guardians, and other caregivers about the offerings and support options of self-help (EC) • Caring professionals, health researchers, and patient advocacy organizations/self-help groups ought to collaborate to exchange knowledge and thereby improve the care and quality of life of people with FASD and the affected families (EC)

Abbreviations: EC, expert consensus; EG, evidence grading (low; moderate; high); FASD, fetal alcohol spectrum disorder; RG, recommendation grading (A—strong recommendation; B—recommendation; 0—open recommendation).

#### Background

Cognitive abilities must be considered when dealing with children/adolescents with FASD, and communication must be adjusted to their developmental level.

## Discussion

With an estimated prevalence of 1.98% in the WHO European Region, FASD represents a significant public health concern. 27 Children diagnosed with FASD encounter a number of challenges that affect their daily functioning and long-term development. The impact of FASD extends beyond childhood and persists into adulthood. Without adequate support and interventions, many children with FASD struggle with everyday situations, preventing them from living independently later in life.

Despite the high prevalence of FASD, there is a lack of studies investigating intervention strategies for children and adolescents with this condition. Particularly concerning are the research gaps regarding enhancing disease management, self-efficacy, and quality of life in this population, prompting an urgent need for further investigation.

The assessment of studies found in the systematic literature research was conducted using the GRADE methodology. However, the limited number of available studies in this research field, combined with their heterogeneity in design, outcomes, and interventions, posed significant challenges for standard comparisons and the calculation of effect estimates. Despite these limitations, GRADE provided a structured framework for evaluating the quality of evidence and allowed us to draw evidence-informed conclusions. In areas where there was insufficient evidence, we were unable to formulate evidence-based recommendations. To address these gaps, we relied on expert consensus, drawing on the knowledge and practical experience of professionals in the field. This approach ensures that even in the absence of strong evidence, high-quality, practice-oriented recommendations can be made, reflecting the current state of knowledge and established clinical practice. The inclusion of expert consensus allows for the formulation of realistic and actionable guidance that is highly relevant in clinical settings.

Existing studies often demonstrate significant qualitative deficiencies. Small study populations hinder sub-analyses of potential confounders such as age, gender, or comorbidities. 28 29 30 31 32 33 34 Study designs without control groups fail to definitively attribute observed effects solely to the interventions themselves and cannot exclude potentially confounding factors such as test-retest effects, the impact of normal development on test results, or the influence of increased attention from study personnel. 30 32 35 36 Additionally, the clinical setting may complicate the transfer of intervention content into real-world settings, leaving the benefits of interventions ambiguous, as children with FASD often suffer from a lack of transfer performance due to executive function disorder. 36 37 38 As some studies examine only a brief intervention period, it is unknown whether extending the intervention duration enhances the therapeutic effect and whether improvements are sustained in the long term. 35 36 39 Moreover, the absence of standardized objective assessment tools makes it difficult to evaluate the efficacy of an intervention. Subjective assessments, such as parent or caregiver surveys, may be biased due to their expectations. 31 38 40 41 42 43 Studies rarely include the perspectives of children or adolescents themselves, 44 mainly reflecting parental or caregiver views. 30 40 41 45 This limitation may partly be caused by the cognitive impairments associated with FASD, as affected individuals may lack the cognitive capacity to understand and adequately respond to questions. Additionally, some children may lack the cognitive maturity to assess their situations accurately, hindering their involvement in the research process. However, by tailoring questions to the cognitive developmental level of the children and addressing individual comprehension issues, children with FASD can be interviewed, as well.

It is essential to note that study populations often include not only children diagnosed with FASD but also those only exposed to prenatal alcohol (PAE), 37 44 46 which does not always lead to the development of the clinical picture of FASD. Even within children with FASD, significant variability exists due to subtypes (FAS, pFAS, and ARND) and individual differences in the neuropsychological profile, heavily influencing intervention effectiveness. Considering the various number of symptoms and their diverse manifestations, interventions for children and adolescents with FASD should be tailored not only to the disorder itself but also to the strengths and weaknesses of the affected individuals.

Individual therapy sessions provide personalized programs tailored to each child's unique abilities and impairments. Particularly, children with severe functional impairments may benefit more from individual therapies than group sessions, where skill variability often leads to suboptimal outcomes. However, group sessions may be more effective for high-functioning children with FASD, enabling them to practice skills within a social peer context, and facilitating integration into daily life.

Beyond the children themselves, parental or caregiver involvement significantly influences intervention effectiveness. Integrating therapy content into family routines profoundly impacts therapy outcomes. Educating caregivers about FASD fosters a deeper understanding of the disorder, creating the framework for providing appropriate support. 31 40 42 43 47 48 Depending on caregivers' cognitive abilities, appropriate language should be used in the communication process and individual impairments need to be considered.

In conclusion, while intervention studies for children and adolescents with FASD are essential for improving outcomes for affected individuals, numerous methodological and practical considerations must be addressed to enhance the validity and applicability of findings, ultimately optimizing intervention strategies for affected children and their families.

## Highlights

- Internationally first evidence-based guideline on interventions for FASD
- Interventions have the potential to improve health outcomes in children with FASD
- Neurocognitive interventions should be adapted to the biological cerebral changes due to PAE
- Psychoeducation for caregivers is recommended to improve children's functioning
- Twenty-one evidence-based recommendations and 26 expert consensus are provided
