# Supplementary material for: First Evidence-Based Guideline for Interventions in FASD
Source: Neuropediatrics. 2025 Mar 19;56(3):160–71. doi: 10.1055/a-2547-4610 (PMC12068929; doi:10.1055/a-2547-4610)
Supplement: Supplementary file 1 — Supplementary Material [file 10-1055-a-2547-4610-s1220243930oa.pdf]

Supplementary Table S1 Members of the guideline group

Guideline coordinators—institutions	Names
Ludwig–Maximilians-University of Munich (LMU), Department of Neuropediatric, Social Pediatric Centre	Prof. Mirjam Landgraf MD
Ludwig–Maximilians-University of Munich (LMU), Department of Neuropediatric, Social Pediatric Centre	Sonja Strieker
Ludwig–Maximilians-University of Munich (LMU), Department of Neuropediatric, Social Pediatric Centre	Prof. Florian Heinen MD
Institute for Evidence in Medicine (IFEM), University of Freiburg	Christine Schmucker MD
Institute for Evidence in Medicine (IFEM), University of Freiburg	Annika Ziegler
Methodological supervision: Association of the Scientific Medical Societies in Germany (AWMF)	Representatives
AWMF-Institute for Medical Knowledge Management, Philipps-University, Marburg	Prof. Ina Kopp MD (Director)<br>Monika Nothacker MD (Vive Director)
German Scientific Societies and Professional Associations	Representatives
Society of Neuropediatric (Germany, Austria, Switzerland; GNP)	Prof. Mirjam Landgraf MD
German Society of Pediatrics and Adolescent Medicine (DGKJ)	Prof. Florian Heinen MD
German Society of Social Pediatrics and Adolescent Medicine (DGSPJ)	Juliane Spiegler MD
German Society of Gynecology and Obstetrics (DGGG)<br>German Society for Prenatal and Obstetric Medicine (DGPGM)	Dietmar Schlembach MD
German Society of Neonatology and Pediatric Intensive Care (GNPI)	Prof. Rolf F. Maier MD
German Society for Perinatal Medicine (DGPM)	Silvia Lobmaier MD
German Society of Child and Adolescent Psychiatry, Psychosomatics and Psychotherapy (DGKJP)	Prof. Christine Freitag MD<br>Substitution: Prof. Frank Häßler MD
German Society of Addiction Research and Addiction Treatment (DG Sucht)	Prof. Bernd Lenz MD
German Society of Addiction Psychology (dg sps)	Prof. Tanja Hoff
German Society of Addiction Medicine (DGS)	Prof. Ulrich Preuss MD<br>Substitution: Prof. Markus Backmund MD
German Association of Midwives (DHV)	Andrea Köbke
Professional Association of Pediatricians (BVKJ)	Matthias Brockstedt MD
Professional Association of Child and Adolescent Psychiatry, Psychosomatics and Psychotherapy (BKJPP)	Annegret Brauer MD
Federal Association of Physicians of the Public Health Services (BVÖGD)	Gabriele Trost-Brinkhues MD
Professional Association of German Psychologists (BDP)	Ralph Schliewenz<br>Substitution: Johanna Thünker
**FASD Experts**	**Names**
Former professor of FASD Centre at Charité University, Berlin	Prof. Hans-Ludwig Spohr
Social Pediatric Centre of Charité University, Berlin	Heike Wolter
Director of the children's home and FASD Centre Sonnenhof, Berlin	Gela Becker<br>Substitution: Lina Schwerg
Director of the Social Pediatric Centre St. Georg, Leipzig	Heike Hoff-Emden MD
FASD Centre, University of Münster	Reinhold Feldmann
FASD Centre at the Social Pediatric Centre at Hospital Ludmillenstift, Meppen	Dorothee Veer MD
Social Pediatric Centre at the Carl-Thiem-Hospital, Cottbus	Kristina Kölzsch MD
FASD Centre for Adults at Elisabeth–Herzberge-Hospital, Berlin	Björn Kruse MD<br>Jessica Wagner
Director of the German Association of the Scientific Medical Societies (AWMF-IMWi)	Prof. Ina Kopp MD (nonvoting)
Child and Adolescent Psychiatry hospital, kbo Heckscher Hospital, Munich	Anna Hutzelmeyer-Nickels MD

(*Continued*)

 © 2025. The Author(s).

Supplementary Table S1 (*Continued*)

Guideline coordinators—institutions	Names
Advocate for Child and Adolescent Rights, specialized in FASD	Gila Schindler
German Patient Support Group FASD Deutschland e.V.	Representatives
President of the Patient Support Group FASD Germany	Gisela Michalowski<br>Substitution: Katrin Lepke
Board Member of the Patient Support Group FASD Germany	Sandra Kramme

Supplementary Table S2 Inclusion criteria (based on PICOS scheme) and exclusion criteria for the systematic literature review

Inclusion criteria/PICOS scheme	
Population	Children and adolescents with fetal alcohol spectrum disorders (aged: 0–18 y)
Intervention	• Drug therapies<br>◦ Stimulants<br>◦ Neuroleptics<br>◦ Food supplements<br>◦ Drugs to regulate the sleep rhythm<br>• Nondrug therapies<br>◦ Psychoeducation of the child/adolescent<br>◦ Psychoeducation of parents/guardians/caregivers<br>◦ Functional, nondrug intervention for the child/adolescent:<br>▪ Occupational therapy<br>▪ Physiotherapy<br>▪ Speech therapy<br>▪ Psychotherapy<br>▪ Training in specific school skills (e.g., mathematics)<br>• Combined medical/nonmedical interventions<br>• Other functional therapies
Comparator	• No intervention<br>• Placebo<br>• Contextual effect<br>• Alternative intervention<br>• Prepost comparison
Outcome	• Improvement in the neuropsychological functions of children/adolescents with FASD, e.g., (relevance 8)<br>◦ Cognitive performance/intelligence<br>◦ Development<br>◦ Epilepsy<br>◦ language<br>◦ Fine-/graphomotoric skills or gross motor coordination<br>◦ Spatial-visual perception or spatial-constructive abilities<br>◦ Executive functions<br>◦ Mathematical skills<br>◦ Learning and memory skills<br>◦ Attention<br>◦ Social skills and behavior<br>• Avoidance of adverse effects of the interventions (relevance 9)<br>• Reduction of complications/secondary diseases, e.g., (relevance 8)<br>◦ Somatic diseases<br>◦ Psychiatric illnesses incl. addictions<br>◦ Risky behavior (risky alcohol/drug consumption, danger to self/others, suicidal acts)<br>◦ School failure and drop-out (or higher rate of school leaving qualifications and vocational training)<br>◦ Delinquency<br>◦ Maltreatment<br>◦ Hospitalization or other inpatient stays<br>• Improving the participation of children/young people with FASD (relevance 9)<br>◦ Learning and application of knowledge<br>◦ General tasks and requirements<br>◦ Communication<br>◦ Mobility

 © 2025. The Author(s).

Supplementary Table S2 (*Continued*)

Inclusion criteria/PICOS scheme	
	◦ Self-care<br>◦ Domestic life<br>◦ Interpersonal interaction and relationships<br>◦ Important areas of life<br>◦ Community, social, and civic life<br>• Improving the quality of life of children/young people with FASD (relevance 9)<br>• Relief for caregivers (biological, foster, and adoptive parents, caregivers) and improving the quality of life of the entire family/institution affected (relevance 8)<br>• Improving knowledge of the deviant state of health/disorder/disability and improvement of insight into the illness (relevance 8)<br>• Improvement in coping and self-efficacy (relevance 8)
Study type	Inclusion of randomized controlled studies, cohort studies, case-control studies, systematic reviews, and meta-analyses
Language	English, German
Exclusion criteria	
A1	Other diseases
A2	Studies with animals or in vitro
A3	No intervention
A4	Study types: case reports, letters, editorials
A5	Unsystematic reviews
A6	Age of the study group predominantly > 18 y (more than 80%)
A7	Published before 2012
A8	Number of participants < 10

 © 2025. The Author(s).

Supplementary Table S3 Search strategies of the systematic literature search

Date of search: August 9, 2022
Databases used: Pubmed, Ebsco, Epistemonikos, Cochrane Library
The search queries consisted of three main components:

1. Target Population—Fetal alcohol spectrum disorder (FASD) and related terms:
   - The search captures various terms related to FASD and associated conditions, including:
     - Direct terms: fetal alcohol spectrum disorder, FASD, alcohol-related birth defects (ARBD), alcohol-related neurodevelopmental disorder (ARND)
     - Combinations of terms such as fetus, embryopathy, prenatal, antenatal with alcohol or ethanol, and various disease-related terms (syndrome, disorder, deficit, effect, exposure)
     - MeSH terms to ensure comprehensive coverage
2. Interventions—Therapies, medications, and complementary measures:
   - Nonpharmacological interventions:
     - Therapeutic approaches such as psychotherapy, psychoeducation, neurofeedback, biofeedback, physiotherapy, occupational therapy, sports therapy, exercise, motor activity, relaxation therapy, workshops, support programs, and education
   - Pharmacological interventions:
     - Medications and active substances, including psychostimulants, psychotropic drugs, antipsychotics, SSRIs, SNRIs, mood stabilizers, valproic acid, melatonin, and other specific substances (e.g., methylphenidate, atomoxetine, benzodiazepines)
   - Nutritional approaches:
     - Dietary supplements, probiotics, vitamins, minerals, and medicinal plants
3. Exclusion criteria—animal studies and in vitro research (not always technically applicable):
   - Studies involving animals or in vitro models are excluded (terms such as animal study, mice, rats, zebrafish, etc.)
     Filters and restrictions (not always technically applicable):
     - Study population: Only humans (filter: humans)
     - Language: Only English and German studies
     - Timeframe: Publications since 2012.

Database (date of search)	Query
Pubmed (August, 9, 2022)	((fetal alcohol spectrum disorder*[tw] OR (FASD*[tiab] AND alcohol*[tiab]) OR alcoholic related birth defect*[tiab] OR alcoholic related neurodevelopmental disorder*[tiab] OR (("fetus"[MH] OR fetus[tiab] OR foetus[tiab] OR fetal[tiab] OR foetal [tiab] OR embryopathy[tiab] OR prenatal*[tiab] OR antenatal*[tiab]) AND (alcohol-*[tiab] OR ethanol[tiab]) AND (disease*[tiab] OR disorder*[tiab] OR syndrome*[tiab] OR deficit*[tiab] OR effect*[tiab] OR expos*[tiab])))<br>AND<br>(("therapeutics"[MH] OR "therapeutic use"[SH] OR "therapy"[SH] OR therap*[tiab] OR intervention*[tiab] OR treatment*[tiab] OR training*[tiab] OR stimulat*[tiab] OR program*[tiab] OR workshop*[tiab] OR support*[tiab] OR "education"[MH] OR education*[tiab] OR ergotherap*[tiab] OR physiotherap*[tiab] OR "Motor Activity"[MH] OR "Sports"[MH] OR sport*[tiab] OR exercise*[tiab] OR physical activit*[tiab] OR hippotherap*[tiab] OR horseback*[tiab] OR "Psychotherapy"[MH] OR psychotherap*[tiab] OR psychoeducation*[tiab] OR neurofeedback*[tiab] OR biofeedback*[tiab] OR rehabilitation*[tiab] OR "Relaxation"[MH] OR "Relaxation Therapy"[MH])<br>OR<br>("Chemicals and Drugs Category"[MH] OR Drug*[tiab] OR medication*[tiab] OR stimulant*[tiab] OR hormon*[tiab] OR "Pharmacological and Toxicological Phenomena"[MH] OR (drug*[tiab] AND (therap*[tiab] OR treatment*[tiab] OR intervention*[tiab])) OR (medic*[tiab] AND (therap*[tiab] OR treatment*[tiab] OR intervention*[tiab])) OR pharmaco*[tiab] OR psychotropic*[tiab] OR psychoactiv*[tiab] OR psychiatric*[tiab] OR adrenergic*[tiab] OR antipsychotic*[tiab] OR analeptic*[tiab] OR psychostimulant*[tiab] OR (tranquilizing[tiab] AND (drug*[tiab] OR agent*[tiab] OR medicin*[tiab] OR medication*[tiab])) OR tryptamin*[tiab] OR melatonin*[tiab] OR methylphenidat*[tiab] OR amphetamin*[tiab] OR amfetamin*[tiab] OR dextroamphetamin*[tiab] OR dextroamfetamin*[tiab] OR dexedrin*[tiab] OR lisdexamphetamine Dimesylate*[tiab] OR lisdexamfetamine Dimesylate*[tiab] OR guanidin*[tiab] OR guanfacin*[tiab] OR atomoxetin*[tiab] OR bupropion*[tiab] OR neuroleptic*[tiab] OR risperidon*[tiab] OR pipamperon*[tiab] OR metylperon*[tiab] OR methylperon*[tiab] OR melperon*[tiab] OR benzodiazepin*[tiab] OR olanzapin*[tiab] OR aripiprazol*[tiab] OR quetiapine Fumarat*[tiab] OR seroquel*[tiab] OR chlorprothixen*[tiab] OR chlorprotixen*[tiab] OR methotrimeprazin*[tiab] OR levomepromazin*[tiab] OR promethazin*[tiab] OR prometazin*[tiab] OR chloral hydrat*[tiab] OR clonidin*[tiab] OR SSRI*[tiab] OR SNRI*[tiab] OR inhibitor*[tiab] OR fluoxetin*[tiab] OR citalopram*[tiab] OR cytalopram*[tiab] OR sertralin*[tiab] OR mood stabilizer*[tiab] OR valproic acid*[tiab] OR divalproex*[tiab] OR lamotrigin*[tiab] OR nutrition*[tiab] OR "Dietary Supplements"[MH] OR ((food*[tiab]

 © 2025. The Author(s).

Supplementary Table S3 (*Continued*)

	OR diet*[tiab]) AND supplement*[tiab]) OR "plants, medicinal"[MH] OR probiotic*[tiab] OR vitamin*[tiab] OR mineral*[tiab]))) NOT (animal study[ti] OR animals study[ti] OR animal survey[ti] OR animals survey[ti] OR animal model*[ti] OR mice[MH] OR mice[ti] OR mouse[ti] OR rats[MH] OR rats[ti] OR rat[ti] OR zebrafish[ti] OR drosophila[ti] OR in vitro[ti]) Filter: Humans, English, German, since 2012
Ebsco (August, 9, 2022)	#1: SU (fetal alcohol syndrome* or fasd or fetal* alcohol spectrum disorder* or prenatal* alcohol exposure* or alcohol* related fetal damage* or alcohol* related birth defect* or alcohol* related neurodevelopmental disorder* or fetal alcohol exposure*) OR TI (fetal alcohol syndrome* or fasd or fetal* alcohol spectrum disorder* or prenatal* alcohol exposure* or alcohol* related fetal damage* or alcohol* related birth defect* or alcohol* related neurodevelopmental disorder* or fetal alcohol exposure*) OR AB (fetal alcohol syndrome* or fasd or fetal* alcohol spectrum disorder* or prenatal* alcohol exposure* or alcohol* related fetal damage* or alcohol* related birth defect* or alcohol* related neurodevelopmental disorder* or fetal alcohol exposure*) #2: SU (therapeutic* OR therap* OR intervention* OR treatment* OR training* OR stimulat* OR program* OR workshop* OR support* OR education* OR ergotherap* OR physiotherap* OR motor Activit* OR sport* OR exercise* OR physical activit* OR hippotherap* OR horseback* OR psychotherap* OR psychoeducation* OR neurofeedback* OR biofeedback* OR rehabilitation* OR Relaxation) OR TI (therapeutic* OR therap* OR intervention* OR treatment* OR training* OR stimulat* OR program* OR workshop* OR support* OR education* OR ergotherap* OR physiotherap* OR motor Activit* OR sport* OR exercise* OR physical activit* OR hippotherap* OR horseback* OR psychotherap* OR psychoeducation* OR neurofeedback* OR biofeedback* OR rehabilitation* OR Relaxation) #3: SU (Drug therapy OR Drug* OR medication* OR stimulant* OR hormon* OR (drug* AND (therap* OR treatment* OR intervention*)) OR (medic* AND (therap* OR treatment* OR intervention*)) OR pharmaco* OR psychotropic* OR psychoactiv* OR psychiatric* OR adrenergic* OR antipsychotic* OR analeptic* OR psychostimulant* OR (tranquilizing AND (drug* OR agent* OR medicin* OR medication*)) OR tryptamin* OR melatonin* OR methylphenidat* OR amphetamin* OR amfetamin* OR dextroamphetamin* OR dextroamfetamin* OR dexedrin* OR lisdexamphetamine Dimesylate* OR lisdexamfetamine Dimesylate* OR guanidin* OR guanfacin* OR atomoxetin* OR bupropion* OR neuroleptic* OR risperidon* OR pipamperon* OR metylperon* OR methylperon* OR melperon* OR benzodiazepin* OR olanzapin* OR aripiprazol* OR quetiapine Fumarat* OR seroquel* OR chlorprothixen* OR chlorprotixen* OR methotrimeprazin* OR levomepromazin* OR promethazin* OR prometazin* OR chloral hydrat* OR clonidin* OR SSRI* OR SNRI* OR inhibitor* OR fluoxetin* OR citalopram* OR cytalopram* OR sertralin* OR mood stabilizer* OR valproic acid* OR divalproex* OR lamotrigin* OR nutrition* OR Dietary Supplements OR ((food* OR diet*) AND supplement*) OR probiotic* OR vitamin* OR mineral*) OR TI (Drug therapy OR Drug* OR medication* OR stimulant* OR hormon* OR (drug* AND (therap* OR treatment* OR intervention*)) OR (medic* AND (therap* OR treatment* OR intervention*)) OR pharmaco* OR psychotropic* OR psychoactiv* OR psychiatric* OR adrenergic* OR antipsychotic* OR analeptic* OR psychostimulant* OR (tranquilizing AND (drug* OR agent* OR medicin* OR medication*)) OR tryptamin* OR melatonin* OR methylphenidat* OR amphetamin* OR amfetamin* OR dextroamphetamin* OR dextroamfetamin* OR dexedrin* OR lisdexamphetamine Dimesylate* OR lisdexamfetamine Dimesylate* OR guanidin* OR guanfacin* OR atomoxetin* OR bupropion* OR neuroleptic* OR risperidon* OR pipamperon* OR metylperon* OR methylperon* OR melperon* OR benzodiazepin* OR olanzapin* OR aripiprazol* OR quetiapine Fumarat* OR seroquel* OR chlorprothixen* OR chlorprotixen* OR methotrimeprazin* OR levomepromazin* OR promethazin* OR prometazin* OR chloral hydrat* OR clonidin* OR SSRI* OR SNRI* OR inhibitor* OR fluoxetin* OR citalopram* OR cytalopram* OR sertralin* OR mood stabilizer* OR valproic acid* OR divalproex* OR lamotrigin* OR nutrition* OR Dietary Supplements OR ((food* OR diet*) AND supplement*) OR probiotic* OR vitamin* OR mineral*) #4: SU (animal research* OR animal stud* OR animal survey OR animal model* OR mice OR mouse OR rat* OR zebrafish OR drosophila OR in vitro) OR TI (animal research* OR animal stud* OR animal survey OR animal model* OR mice OR mouse OR rat* OR

(*Continued*)

 © 2025. The Author(s).

Supplementary Table S3 (*Continued*)

	zebrafish OR drosophila OR in vitro)<br>#5:<br>(#1 AND (#2 OR #3)) NOT #4<br>Limited: since 2012
Epistemonikos (August, 9, 2022)	((title:((FASD AND alcohol*) OR “alcohol-related birth defect” OR “alcohol-related neurodevelopmental disorder” OR ((fetus OR fetus OR fetal* OR fetal* OR embryopathy OR prenatal* OR antenatal*) AND (alcohol* OR ethanol*) AND (disease* OR disorder* OR syndrome* OR deficit* OR effect* OR expos*))) OR abstract:((FASD AND alcohol*) OR “alcohol-related birth defect” OR “alcohol-related neurodevelopmental disorder” OR ((fetus OR fetus OR fetal* OR fetal* OR embryopathy OR prenatal* OR antenatal*) AND (alcohol* OR ethanol*) AND (disease* OR disorder* OR syndrome* OR deficit* OR effect* OR expos*))))<br>AND<br>(title:(therapeutic* OR therap* OR intervention* OR treatment* OR training* OR stimulat* OR program* OR workshop* OR support* OR education* OR ergotherap* OR physiotherap* OR sport* OR exercise* OR physical activit* OR hippotherap* OR horseback* OR psychotherap* OR psychoeducation* OR neurofeedback* OR biofeedback* OR rehabilitation* OR relaxation) OR abstract:(therapeutic* OR therap* OR intervention* OR treatment* OR training* OR stimulat* OR program* OR workshop* OR support* OR education* OR ergotherap* OR physiotherap* OR sport* OR exercise* OR physical activit* OR hippotherap* OR horseback* OR psychotherap* OR psychoeducation* OR neurofeedback* OR biofeedback* OR rehabilitation* OR relaxation))<br>OR<br>(title:(drug* OR medication* OR stimulant* OR hormon* OR (drug* AND (therap* OR treatment* OR intervention*)) OR (medic* AND (therap* OR treatment* OR intervention*)) OR pharmaco* OR psychotropic* OR psychoactiv* OR psychiatric* OR adrenergic* OR antipsychotic* OR analeptic* OR psychostimulant* OR (tranquilizing AND (drug* OR agent* OR medicin* OR medication*)) OR tryptamin* OR melatonin* OR methylphenidat* OR amphetamin* OR amfetamin* OR dextroamphetamin* OR dextroamfetamin* OR dexedrin* OR lisdexamphetamine Dimesylate* OR lisdexamfetamine Dimesylate* OR guanidin* OR guanfacin* OR atomoxetin* OR bupropion* OR neuroleptic* OR risperidon* OR pipamperon* OR metylperon* OR methylperon* OR melperon* OR benzodiazepin* OR olanzapin* OR aripiprazol* OR quetiapine Fumarat* OR seroquel* OR chlorprothixen* OR chlorprotixen* OR methotrimeprazin* OR levomepromazin* OR promethazin* OR prometazin* OR chloral hydrat* OR clonidin* OR SSRI* OR SNRI* OR inhibitor* OR fluoxetin* OR citalopram* OR cytalopram* OR sertralin* OR mood stabilizer* OR valproic acid* OR divalproex* OR lamotrigin* OR nutrition* OR ((food* OR diet*) AND supplement*) OR probiotic* OR vitamin* OR mineral*) OR abstract:(drug* OR medication* OR stimulant* OR hormon* OR (drug* AND (therap* OR treatment* OR intervention*)) OR (medic* AND (therap* OR treatment* OR intervention*)) OR pharmaco* OR psychotropic* OR psychoactiv* OR psychiatric* OR adrenergic* OR antipsychotic* OR analeptic* OR psychostimulant* OR (tranquilizing AND (drug* OR agent* OR medicin* OR medication*)) OR tryptamin* OR melatonin* OR methylphenidat* OR amphetamin* OR amfetamin* OR dextroamphetamin* OR dextroamfetamin* OR dexedrin* OR lisdexamphetamine Dimesylate* OR lisdexamfetamine Dimesylate* OR guanidin* OR guanfacin* OR atomoxetin* OR bupropion* OR neuroleptic* OR risperidon* OR pipamperon* OR metylperon* OR methylperon* OR melperon* OR benzodiazepin* OR olanzapin* OR aripiprazol* OR quetiapine Fumarat* OR seroquel* OR chlorprothixen* OR chlorprotixen* OR methotrimeprazin* OR levomepromazin* OR promethazin* OR prometazin* OR chloral hydrat* OR clonidin* OR SSRI* OR SNRI* OR inhibitor* OR fluoxetin* OR citalopram* OR cytalopram* OR sertralin* OR mood stabilizer* OR valproic acid* OR divalproex* OR lamotrigin* OR nutrition* OR ((food* OR diet*) AND supplement*) OR probiotic* OR vitamin* OR mineral*)))<br>NOT<br>(title:(“animal study” OR “animals study” OR “animal survey” OR “animals survey” OR “animal model” OR “animal models” OR mice OR mouse OR rats OR rat OR zebrafish OR drosophila OR “in vitro”))<br>Limited: since 2012 $\geq$ 431 results<br>Limited: systematic reviews $\geq$ 168 results
Cochrane Library (August, 9, 2022)	[[Keywords: fetal* alcohol* syndrome*] OR [Keywords: fasd] OR [Keywords: fetal* alcohol* spectrum disorder*] OR [Keywords: prenatal* alcohol* exposure*] OR [Keywords: alcohol* related fetal* damage*] OR [Keywords: alcohol* related birth defect*] OR [Keywords: alcohol* related neurodevelopmental disorder*] OR [Keywords: fetal*

 © 2025. The Author(s).

Supplementary Table S3 (*Continued*)

	alcohol* exposure*]] AND [[Keywords: therapeutic*] OR [Keywords: therap*] OR [Keywords: intervention*] OR [Keywords: treatment*] OR [Keywords: training*] OR [Keywords: stimulat*] OR [Keywords: program*] OR [Keywords: workshop*] OR [Keywords: support*] OR [Keywords: education*] OR [Keywords: ergotherap*] OR [Keywords: physiotherap*] OR [Keywords: motor activit*] OR [Keywords: sport*] OR [Keywords: exercise*] OR [Keywords: physical activit*] OR [Keywords: hippotherap*] OR [Keywords: horseback*] OR [Keywords: psychotherap*] OR [Keywords: psycho-education*] OR [Keywords: neurofeedback*] OR [Keywords: biofeedback*] OR [Keywords: rehabilitation*] OR [Keywords: relaxation] OR [Keywords: drug therap*] OR [Keywords: drug*] OR [Keywords: medication*] OR [Keywords: stimulant*] OR [Keywords: hormon*] OR [[Keywords: drug*] AND [[Keywords: therap*] OR [Keywords: treatment*] OR [Keywords: intervention*]]] OR [[Keywords: medic*] AND [[Keywords: therap*] OR [Keywords: treatment*] OR [Keywords: intervention*]]] OR [Keywords: pharmaco*] OR [Keywords: psychotropic*] OR [Keywords: psychoactiv*] OR [Keywords: psychiatric*] OR [Keywords: adrenergic*] OR [Keywords: antipsychotic*] OR [Keywords: analeptic*] OR [Keywords: psychostimulant*] OR [[Keywords: tranquilizing] AND [[Keywords: drug*] OR [Keywords: agent*] OR [Keywords: medicin*] OR [Keywords: medication*]]] OR [Keywords: tryptamin*] OR [Keywords: melatonin*] OR [Keywords: methyl*enidat*] OR [Keywords: am*etamin*] OR [Keywords: dextro-am*etamin*] OR [Keywords: dexedrin*] OR [Keywords: lisdexam*etamine dimesylate*] OR [Keywords: guanidin*] OR [Keywords: guanfacin*] OR [Keywords: atomoxetin*] OR [Keywords: bupropion*] OR [Keywords: neuroleptic*] OR [Keywords: risperidon*] OR [Keywords: pipamperon*] OR [Keywords: metylperon*] OR [Keywords: methylperon*] OR [Keywords: melperon*] OR [Keywords: benzodiazepin*] OR [Keywords: olanzapin*] OR [Keywords: aripiprazol*] OR [Keywords: quetiapine fumarat*] OR [Keywords: seroquel*] OR [Keywords: chlorprothixen*] OR [Keywords: chlorprotixen*] OR [Keywords: methotrimeprazin*] OR [Keywords: levomepromazin*] OR [Keywords: promethazin*] OR [Keywords: prometazin*] OR [Keywords: chloral hydrat*] OR [Keywords: clonidin*] OR [Keywords: ssri*] OR [Keywords: snri*] OR [Keywords: inhibitor*] OR [Keywords: fluoxetin*] OR [Keywords: citalopram*] OR [Keywords: cytalopram*] OR [Keywords: sertralin*] OR [Keywords: mood stabilizer*] OR [Keywords: valproic acid*] OR [Keywords: divalproex*] OR [Keywords: lamotrigin*] OR [Keywords: nutrition*] OR [Keywords: dietary supplement*] OR [[[Keywords: food*] OR [Keywords: diet*]] AND [Keywords: supplement*]] OR [Keywords: probiotic*] OR [Keywords: vitamin*] OR [Keywords: mineral*]] AND [Earliest: (January 1, 2012–August 31, 2012]

Supplementary Document 1: Publications included in the systematic evidence classification

Original Publications

1 Boroda E, Krueger AM, Bansal P, et al. A randomized controlled trial of transcranial direct-current stimulation and cognitive training in children with fetal alcohol spectrum disorder. Brain Stimul 2020;13(04):1059–1068

2 Coles CD, Kable JA, Taddeo E, Strickland D. GoFAR: improving attention, behavior and adaptive functioning in children with fetal alcohol spectrum disorders: brief report. Dev Neurorehabil 2018;21(05):345–349

3 Coles CD, Kable JA, Taddeo E, Strickland DC. A metacognitive strategy for reducing disruptive behavior in children with fetal alcohol spectrum disorders: GoFAR pilot. Alcohol Clin Exp Res 2015;39(11):2224–2233

4 Graham DM, Glass L, Mattson SN. The influence of extrinsic reinforcement on children with heavy prenatal alcohol exposure. Alcohol Clin Exp Res 2016;40(02):348–358

5 Jirikowic T, Westcott McCoy S, Price R, Ciol MA, Hsu LY, Kartin D. Virtual sensorimotor training for balance: pilot study results for children with fetal alcohol spectrum disorders. Pediatr Phys Ther 2016;28(04):460–468

6 Kable JA, Coles CD, Strickland D, Taddeo E. Comparing the effectiveness of on-line versus in-person caregiver education and training for behavioral regulation in families of children with FASD. Int J Ment Health Addict 2012;10(06):791–803

7 Kable JA, Taddeo E, Strickland D, Coles CD. Community translation of the math interactive learning experience program for children with FASD. Res Dev Disabil 2015;39:1–11

8 Kable JA, Taddeo E, Strickland D, Coles CD. Improving FASD children's self-regulation: piloting phase 1 of the GoFAR intervention. Child Fam Behav Ther 2016;38(02):124–141

9 Kerns KA, Macoun S, MacSween J, Pei J, Hutchison M. Attention and working memory training: a feasibility study in children with neurodevelopmental disorders. Appl Neuropsychol Child 2017;6 (02):120–137

10 Kully-Martens K, Pei J, Kable J, Coles CD, Andrew G, Rasmussen C. Mathematics intervention for children with fetal alcohol spectrum disorder: A replication and extension of the math interactive learning experience (MILE) program. Res Dev Disabil 2018; 78:55–65

11 Leenaars LS, Denys K, Henneveld D, Rasmussen C. The impact of fetal alcohol spectrum disorders on families: evaluation of a family intervention program. Community Ment Health J 2012; 48(04):431–435

12 McCoy SW, Jirikowic T, Price R, et al. Virtual sensorimotor balance training for children with fetal alcohol spectrum disorders: feasibility study. Phys Ther 2015;95(11):1569–1581

13 Nash K, Stevens S, Greenbaum R, Weiner J, Koren G, Rovet J. Improving executive functioning in children with fetal alcohol spectrum disorders. Child Neuropsychol 2015;21(02):191–209

 © 2025. The Author(s).

14 Nguyen TT, Risbud RD, Mattson SN, Chambers CD, Thomas JD. Randomized, double-blind, placebo-controlled clinical trial of choline supplementation in school-aged children with fetal alcohol spectrum disorders. Am J Clin Nutr 2016;104(06):1683–1692
15 O'Connor MJ, Laugeson EA, Mogil C, et al. Translation of an evidence-based social skills intervention for children with prenatal alcohol exposure in a community mental health setting. Alcohol Clin Exp Res 2012;36(01):141–152
16 O'Connor MJ, Quattlebaum J, Castañeda M, Dipple KM. Alcohol intervention for adolescents with fetal alcohol spectrum disorders: project step up, a treatment development study. Alcohol Clin Exp Res 2016;40(08):1744–1751
17 Petrenko CLM, Demeusy EM, Alto ME. Six-month follow-up of the families on track intervention pilot trial for children with fetal alcohol spectrum disorders and their families. Alcohol Clin Exp Res 2019;43(10):2242–2254
18 Petrenko CLM, Pandolfino ME, Robinson LK. Findings from the families on track intervention pilot trial for children with fetal alcohol spectrum disorders and their families. Alcohol Clin Exp Res 2017;41(07):1340–1351
19 Regehr E. The Impact of an Intervention on Social Skills of Young Children with Prenatal Alcohol Exposure [Master's Thesis, University of Alberta]. Alberta. Accessed 2015 at: https://dx.doi.org/10.7939/r3b56dc77
20 Śmiarowska M, Brzuchalski B, Grzywacz E, et al. Influence of *COMT* (rs4680) and *DRD2* (rs1076560, rs1800497) gene polymorphisms on safety and efficacy of methylphenidate treatment in children with fetal alcohol spectrum disorders. Int J Environ Res Public Health 2022;19(08):4479
21 Smith SM, Virdee MS, Eckerle JK, et al. Polymorphisms in SLC44A1 are associated with cognitive improvement in children diagnosed with fetal alcohol spectrum disorder: an exploratory study of oral choline supplementation. Am J Clin Nutr 2021;114(02):617–627
22 Soh DW, Skocic J, Nash K, Stevens S, Turner GR, Rovet J. Self-regulation therapy increases frontal gray matter in children with fetal alcohol spectrum disorder: evaluation by voxel-based morphometry. Front Hum Neurosci 2015;9:108
23 Vidal R, Vidal L, Ristol F, et al. Dog-assisted therapy for children and adolescents with fetal alcohol spectrum disorders a randomized controlled pilot study. Front Psychol 2020;11:1080
24 Wells AM, Chasnoff IJ, Schmidt CA, Telford E, Schwartz LD. Neurocognitive habilitation therapy for children with fetal alcohol spectrum disorders: an adaptation of the alert program. Am J Occup Ther 2012;66(01):24–34
25 Wozniak JR, Fink BA, Fuglestad AJ, et al. Four-year follow-up of a randomized controlled trial of choline for neurodevelopment in fetal alcohol spectrum disorder. J Neurodev Disord 2020;12(01):9
26 Wozniak JR, Fuglestad AJ, Eckerle JK, et al. Choline supplementation in children with fetal alcohol spectrum disorders: a randomized, double-blind, placebo-controlled trial. Am J Clin Nutr 2015;102(05):1113–1125
27 Wozniak JR, Fuglestad AJ, Eckerle JK, et al. Choline supplementation in children with fetal alcohol spectrum disorders has high feasibility and tolerability. Nutr Res 2013;33(11):897–904
28 Zarnegar Z, Hambrick EP, Perry BD, Azen SP, Peterson C. Clinical improvements in adopted children with fetal alcohol spectrum disorders through neurodevelopmentally informed clinical intervention: a pilot study. Clin Child Psychol Psychiatry 2016;21(04):551–567

Systematic Reviews

1 Flannigan K, Coons-Harding KD, Anderson T, et al. A systematic review of interventions to improve mental health and substance use outcomes for individuals with prenatal alcohol exposure and fetal alcohol spectrum disorder. Alcohol Clin Exp Res 2020;44(12):2401–2430
2 Mela M, Okpalauwaekwe U, Anderson T, et al. The utility of psychotropic drugs on patients with fetal alcohol spectrum disorder (FASD): a systematic review. Psychiatry Clin Psychopharmacol 2018;28(04):436–445
3 Ordenewitz LK, Weinmann T, Schlüter JA, et al. Evidence-based interventions for children and adolescents with fetal alcohol spectrum disorders - a systematic review. Eur J Paediatr Neurol 2021;33:50–60
4 Reid N, Dawe S, Shelton D, et al. Systematic review of fetal alcohol spectrum disorder interventions across the life span. Alcohol Clin Exp Res 2015;39(12):2283–2295

Supplementary Document 2: Risk of Bias Assessment

Risk of bias assessment—original studies

<table>
<tr><th>Reference
study type</th><th colspan="2">Participants (number and characteristics)</th><th colspan="2">Drop-outs</th><th>Intervention</th></tr>
<tr><td>Smiarowska et al[1]
Uncontrolled in-
tervention study</td><td colspan="2">–Inclusion criteria: children (>6 y old) with ADHD and hPAE who did not benefit from cognitive behavioral therapy for the last 6 mo or who have severe ADHD symptoms and dysfunctional environmental functioning
–Exclusion criteria: psychiatric or developmental disorders
–Enrolled: $n = 303$
–Included: $n = 114$
–Age: > 6 y</td><td colspan="2">NA</td><td>MPH:
–20 mg MPH hydrochloride or 36 mg MPH
–Maximum dose (60 mg MPH hydrochloride or 36 mg MPH) was used only in individual cases
–Daily doses for 4 wks</td></tr>
<tr><td rowspan="2">Nguyen et al[2]
RCT (multisite, randomized, dou-
ble-blinded, place-
bo-controlled, par-
allel-group clinical trial)</td><td colspan="2">–Inclusion criteria: children with confirmed hPAE; primary English speakers
–Exclusion criteria: head injury, substantial physical or psychiatric disability; any other causes of mental deficiency; prescription of medication with risk of atherosclerosis
–5–10 y old</td><td colspan="2" rowspan="2">Discontinued intervention: $n = 0$</td><td rowspan="2">Choline:
–625 mg choline (in the form of a glycerophosphocholine liquid concentrate (5.25 mL/d))
–Daily doses for 6 wks</td></tr>
<tr><td>Choline group:
–Assigned: $n = 30$
–Received: $n = 29$
–Lost to follow-up: $n = 1$
–Completed: $n = 28$
–Analyzed with in-
tention-to-treat: $n = 29$</td><td>Placebo group:
–Assigned: $n = 28$
–Received: $n = 26$
–Lost to follow-up: $n = 2$
–Completed: $n = 24$
–Analyzed with intention-to-treat: $n = 26$</td></tr>
<tr><td rowspan="2">Wozniak et al[3]
RCT (double-blind, randomized, pla-
cebo-controlled trial) pilot study</td><td colspan="2">–Inclusion: Children with FASD diagnosis
–Exclusion: developmental or neurological disorder; other medical conditions affecting the brain
–No exclusion: psychiatric co-morbidities (ADHD)
–2,5–4,9 y old</td><td rowspan="2">Choline:
Discontin-
ued: $n = 1$ (refused to test agent for more than 1 mo)</td><td rowspan="2">Placebo:
Discontinued: $n = 1$ (declined to continue)</td><td rowspan="2">Choline:
–1.25 g choline bitartrate powder delivering 500 mg choline
–Daily doses for 9 mo</td></tr>
<tr><td>Choline group:
–Assigned: $n = 13$
–Received: $n = 10$
–Lost to follow-up: $n = 0$
–Completed: $n = 9$
–Analyzed with in-
tention-to-treat: $n = 10$</td><td>Placebo group:
–Assigned: $n = 12$
–Received: $n = 10$
–Lost to follow-up: $n = 1$
–Completed: $n = 8$
–Analyzed with intention-to-treat: $n = 10$</td></tr>
<tr><td rowspan="2">Wozniak et al[4]
RCT (randomized, double-blind, pla-
cebo-controlled pi-
lot trial)</td><td colspan="2">–Inclusion: Children with confirmed hPAE or suspected hPAE with dysmorphic faces and cognitive deficits
–Exclusion: developmental or neurological disorder; traumatic brain injury; other medical conditions
–No exclusion: psychiatric co-morbidity (ADHD or learning disorder)
–2,5–5 y old</td><td rowspan="2">Choline:
Discontin-
ued: $n = 5$ (declined to contin-
ue: $n = 4$; refused in-
tervention: $n = 1$)</td><td rowspan="2">Placebo:
Discontinued: $n = 3$ (declined to continue)</td><td rowspan="2">Choline:
–1.25 g choline bitartrate powder delivering 500 mg choline
–Daily doses for 9 mo</td></tr>
<tr><td>Choline:
–Assigned: $n = 34$
–Received: $n = 31$
–Lost to follow-up: $n = 0$
–Completed: $n = 26$
–Analyzed with in-
tention-to-treat: $n = 31$</td><td>Placebo:
–Assigned: $n = 31$
–Received: $n = 29$
–Lost to follow-up: $n = 1$
–Completed: $n = 25$
–Analyzed with intention-to-treat: $n = 29$</td></tr>
</table>

(*Continued*)

 © 2025. Thieme. All rights reserved.

(*Continued*)

Reference<br>study type	Participants (number and characteristics)		Drop-outs		Intervention
Wozniak et al[5]<br>4-y follow-up of an RCT (randomized, double-blind, placebo-controlled trial)	–Inclusion: Children with confirmed hPAE or suspected hPAE with dysmorphic faces and cognitive deficits; supplement adherence in initial trial > 50% of days<br>–Exclusion: developmental or neurological disorder; traumatic brain injury; other medical conditions<br>–No exclusion: psychiatric co-morbidity (ADHD or learning disorder)<br>–2,5–5 y old in initial trial		Choline:<br>Initial trial:<br>Discontinued: $n = 5$ (declined to continue: $n = 4$; refused intervention: $n = 1$)	Placebo:<br>Initial trial:<br>Discontinued: $n = 3$ (declined to continue)	Choline:<br>–1.25 g choline bitartrate powder delivering 513 mg choline<br>–Daily doses for 9 mo
	Choline:<br>Initial trial:<br>–Assigned: $n = 34$<br>–Received: $n = 31$<br>–Lost to follow-up: $n = 0$<br>–Completed: $n = 26$<br>–Analyzed with intention-to-treat: $n = 31$<br>Follow-up:<br>–Lost to follow-up: $n = 9$<br>–Analyzed: $n = 15$	Placebo:<br>Initial trial:<br>–Assigned: $n = 31$<br>–Received: $n = 29$<br>–Lost to follow-up: $n = 1$<br>–Completed: $n = 25$<br>–Analyzed with intention-to-treat: $n = 29$<br>Follow-up:<br>–Lost to follow-up: $n = 8$<br>–Analyzed: $n = 16$			
Smith et al[6]<br>Retrospective analysis of an RCT (randomized, double-blind, placebo-controlled trial)	–Inclusion: Children with confirmed hPAE or suspected hPAE with dysmorphic faces and cognitive deficits; supplement adherence in initial trial > 50% of days; providing a blood sample for genomics<br>–Exclusion: developmental or neurological disorder; traumatic brain injury; other medical conditions<br>–No exclusion: psychiatric co-morbidity (ADHD or learning disorder)<br>–2,5–5 y old in initial trial		Choline:<br>Initial trial:<br>Discontinued: $n = 5$	Placebo:<br>Initial trial:<br>Discontinued: $n = 3$	Choline:<br>–1.25 g choline bitartrate powder delivering 500 mg choline<br>–Daily doses for 9 mo
	Choline:<br>Initial trial:<br>–Assigned: $n = 34$<br>–Received: $n = 31$<br>–Lost to follow-up: $n = 0$<br>–Completed: $n = 26$<br>–Blood sample: $n = 26$<br>–Analyzed with intention-to-treat: $n = 31$<br>Follow-up:<br>–Lost to follow-up: $n = 11$<br>–Analyzed: $n = 15$	Placebo:<br>Initial trial:<br>–Assigned: $n = 31$<br>–Received: $n = 29$<br>–Lost to follow-up: $n = 1$<br>–Blood sample: $n = 26$<br>–Completed: $n = 25$<br>–Analyzed with intention-to-treat: $n = 29$<br>Follow-up:<br>–Lost to follow-up: $n = 9$<br>–Analyzed: $n = 16$			

 © 2025. Thieme. All rights reserved.

(*Continued*)

Reference study type	Participants (number and characteristics)	Drop-outs	Intervention
Boroda et al[7]<br>RCT	–Inclusion criteria: documented history of heavy PAE; or suspected of heavy PAE with full-FAS diagnosis based on dysmorphology; at baseline characterized according to modified IOM criteria<br>–Age: 9–16 y<br>tDCS:<br>–Assigned: $n = 20$<br>–Lost to follow-up: $n = 0$<br>–Analyzed: $n = 19$<br>Sham stimulation augmented (sham):<br>–Assigned: $n = 24$<br>–Lost to follow-up: $n = 2$<br>–Analyzed: $n = 19$	tDCS:<br>Discontinued: $n = 1$ (stimulation discomfort)<br>Sham:<br>Discontinued: $n = 3$ (stimulation discomfort: $n = 1$, time commitment: $n = 2$)	tDCS group:<br>2 parallel components:<br>–Cognitive training: 5 tasks from BrainHQ focussing on working memory and attention. Tasks were completed 4 times (total of 46 min) during each of 5 weekly sessions<br>–tDCS: transcranial stimulation was initiated 30 s (at 2mA intensity) prior to cognitive training and lasted 13 min. Afterward, it turned off and stayed off for 20 min. Then it turned on again for 13 min.
Vidal et al[8]<br>RCT (randomized, rater-blinded, controlled pilot trial)	–Inclusion: FASD diagnosis (FAS, pFAS, ARND); 6–18 y of age; with stabilized doses of medication for at least 2 mo before the study<br>–No exclusion: comorbidities, borderline IQ/intellectual disability<br>DAT:<br>–Assigned: $n = 19$<br>–Completed: $n = 17$<br>–Lost at posttreatment: $n = 0$<br>–Analyzed: $n = 17$<br>TAU:<br>–Assigned: $n = 20$<br>–Completed: $n = 16$<br>–Lost at posttreatment: $n = 1$<br>–Analyzed: $n = 16$	DAT:<br>Discontinued medication: $n = 2$<br>TAU:<br>Dropped-out: $n = 1$; Discontinued medication: $n = 2$	DAT:<br>–12 manualized sessions in 2 phases (6 individual sessions, 6 group activity sessions<br>–Sessions included 2 certified therapy dogs<br>–Groups of 3–4 patients<br>–Weekly 45-min sessions for about 3 mo<br>–Pharmacological treatment as usual
Kerns et al[9]<br>Uncontrolled intervention study	–Inclusion: Children with diagnosed FASD or Autism Spectrum Disorder who receive Educational Assistant Support within their school program<br>–Exclusion: history of traumatic brain injury, chronic health problem, inability to verbally communicate, or diagnosis of an intellectual disability based on information provided by special education staff and parent or caregiver<br>–Age: 6–13 y old<br>–Enrolled: $n = 23$<br>–Completed: $n = 17$<br>–Analyzed: $n = 17$ (Children with FASD: $n = 10$<br>Children with Autism Spectrum Disorder: $n = 7$)	Discontinued: $n = 6$ (Education Assistant scheduling difficulties)	Caribbean Quest:<br>–Video game with one-to-one support by a trained and tested educational assistant using metacognitive strategies<br>–Game consisting of 5 hierarchically structured self-adjusting mini-games to improve attention and working memory<br>–30-min sessions, 2–3 times a week over a 10–12 wk span
Kable et al[10]<br>RCT	–Inclusion: clinical diagnosis of FAS/pFAS or significant levels of alcohol-related dysmorphia<br>–Exclusion: IQ < 50; diagnosis of mental health problems interfering with learning; no stable placement<br>–Parents needed to complete two workshops (education about neurodevelopmental characteristics of FASD; strategies to deal with behavioral regulation problems)<br>–Age: 3–10 y old<br>–Recruited: $n = 68$	Centre MILE:<br>Discontinued: $n = 1$ (session 3-travel and time)<br>Community MILE:<br>Discontinued: $n = 1$ (session 4-scheduling conflicts/ travel)<br>Parents Instruction:<br>Discontinued: $n = 0$	MILE:<br>–Parents completed workshops and received a manual discussing math learning in children with FASD and strategies for facilitating math learning at home<br>–MILE: Program targeting learning behavior and math development and focussing on the core deficit of mathematical competence (metacognitive control strategies adapted from FAR)<br>–One-on-one individualized tutorial sessions by trained instructor<br>–Weekly home assignments<br>–Weekly sessions for 15 wks

(*Continued*)

 © 2025. Thieme. All rights reserved.

(*Continued*)

Reference study type	Participants (number and characteristics)		Drop-outs		Intervention
Kully-Martens et al[11]<br>CCT (not randomized)	–Inclusion: confirmed PAE or FASD diagnosis<br>–No exclusion: common mental health comorbidities<br>–Enrolled: $n=29$<br>–Age: 4–10 y		MILE:<br>Discontinued: $n=0$	SSIS:<br>Discontinued: $n=0$	MILE:<br>–No parent workshops<br>–MILE: Program targeting learning behavior and math development and focussing on the core deficit of mathematical competence (metacognitive control strategies adapted from FAR)<br>–One-on-one individualized tutorial sessions by trained instructor<br>–Weekly home assignments<br>–10–30-min sessions once/twice a wk for 6–8 wks
	MILE:<br>–Assigned: $n=15$<br>–Received: $n=15$<br>–Lost to immediate follow-up: $n=0$<br>–Analyzed (immediate): $n=15$<br>–Lost to 6-mo follow-up: $n=3$<br>–Analyzed (6-mo): $n=12$	SSIS:<br>–Assigned: $n=13$<br>–Received: $n=13$<br>–Lost to immediate follow-up: $n=0$<br>–Analyzed (immediate): $n=13$<br>–Lost to 6-mo follow-up: $n=6$<br>–Analyzed (6-mo): $n=7$			
Wells et al[12]<br>RCT (rater-blinded)	–Inclusion: confirmed PAE, FAS, or ARND diagnosis<br>–Exclusion: serious head trauma; current/historical lead poisoning; genetic/dysmorphic syndrome (other than FAS)<br>–No exclusion: exposures to other drugs (marijuana/cocaine)<br>–Age: 6–11 y<br>–Eligible for enrolment: $n=90$<br>–Enrolled: $n=78$		NA		NHT:<br>–Parents received feedback and recommendations regarding the child's behavior, learning, and emotional functioning<br>–Parent training: psychoeducation in a group setting<br>–Children's' training:<br>–NHT: program in a group setting teaching children to recognize individual deficits and to develop strategies to compensate for them (integration of techniques of therapy of traumatic brain injury, and Alert Program: analogy of car engine)<br>–Conjoined parent and children training at the end of each session<br>–Weekly 75-min sessions for 12 wks
	Neurocognitive habilitation therapy (NHT):<br>$n=40$	Control:<br>$n=38$			

 © 2025. Thieme. All rights reserved.

(*Continued*)

Reference study type	Participants (number and characteristics)	Drop-outs	Intervention
Nash et al[13]<br>CCT (not randomized)	–Inclusion: Children with FASD diagnosis<br>–Exclusion: IQ < IQ 70<br>–Age: 8–12 y<br>TXT:<br>–Assigned: $n = 14$<br>–Completed: $n = 12$<br>–Received: $n = 15$<br>–Analyzed (immediate): $n = 12$<br>–Lost to 6-mo follow-up: $n = 3$<br>–Analyzed (6-mo): $n = 9$<br>Delayed treatment control group (DTC):<br>–Assigned: $n = 15$<br>–Completed: $n = 13$<br>–Received: $n = 15$<br>–Analyzed (immediate): $n = 13$	No completion: $n = 4$<br>(3 children (1 TXT, 2 DTC) had custody access issues and did not continue after baseline testing; and 1 child was lost to follow-up between the initial screening interview and scheduling of baseline testing)	TXT:<br>–Alert: Program targeting self-regulation skills through sensory integration and cognitive processing activities (analogy of a car engine) in three stages: awareness, self-regulation strategies, independent usage<br>–12 1-h sessions for 14 wks
Soh et al[14]<br>CCT (not randomized)	Treatment and waiting list group:<br>–Inclusion: Children with FASD included in clinic files or children in FASD support groups<br>–Exclusion: Head injury requiring hospitalization, other neurological abnormalities, a debilitating or chronic medical condition, contraindications to MRI (e.g., braces, other implanted metal devices)<br>–No exclusion: ADHD<br>Healthy control group:<br>–Inclusion: Children without PAE, psychiatric diagnosis (e.g., ADHD) or learning disability<br>In total:<br>–Age: 8–12 y<br>–Recruited: $n = 65$	TXT:<br>Before pre-test:<br>$n = 3$<br>(drop-out: 1; refused scan:2)<br>After pre-test:<br>Drop-out:<br>$n = 1$; refused scan:<br>$n = 1$)<br>DTC:<br>Before pre-test:<br>$n = 1$ (refused scan)<br>After pre-test:<br>$n = 1$<br>(movement)<br>After post-test: $n = 2$<br>(movement)<br>CT:<br>After post-test: $n = 7$<br>(undisclosed exposure: 1; low IQ/ learning disability: 2, technical problems: 1; movement: 1; braces: 2)<br>After post-test: $n = 1$<br>(movement)	TXT:<br>–Alert: Program targeting self-regulation skills through sensory integration and cognitive processing activities (analogy of a car engine) in three stages: awareness, self-regulation strategies, independent usage<br>–12 1,5-h sessions for 14 wks
Coles et al[15]<br>RCT	–Inclusion: Children with PAE with significant levels of alcohol-related physical features or with a clinical diagnosis of FAS/pFAS<br>–Age: 5–10 y<br>–Recruited: 30 children	GoFAR:<br>Drop-out:<br>$n = 3$ (unknown)<br>FACE-LAND:<br>Drop-out:<br>$n = 3$ (family crisis: 2; unknown: 1)<br>Control:<br>Drop-out:<br>$n = 1$ (unknown)	GoFAR with 3 components:<br>–Children: Children learn metacognitive control strategies (FAR methodology) through computer games (5 weekly sessions)<br>–Parents: Parents learn about the neurodevelopmental/ behavioral impacts of PAE and how to facilitate the child's behavioral regulation skills (5 weekly 1-h sessions parallel to children's sessions)<br>–Children + Parents: Behavior analogue therapy (BAT): Children and parents apply the FAR methodology in everyday contexts (5 weekly sessions after 5 wks of children and parent training)

(*Continued*)

 © 2025. Thieme. All rights reserved.

(*Continued*)

Reference study type	Participants (number and characteristics)	Drop-outs	Intervention
Coles et al[16]<br>RCT	–Inclusion: Children with PAE with significant levels of alcohol-related physical features or with a clinical diagnosis of FAS/pFAS<br>–Parents needed to attend a group workshop on the impact of PAE on neuro-developmental functioning before enrolment<br>–Age: 5–10 y<br>–Recruited: $n = 30$<br>–Completed: $n = 25$	NA	GoFAR with 3 components:<br>–Children: Children learn metacognitive control strategies<br>(FAR methodology) through computer games (5 weekly sessions)<br>–Parents: Parents learn about the neurodevelop-mental/behavioral<br>impacts of PAE and how to facilitate the child's behavioral regulation<br>skills (5 weekly 1-h sessions parallel to children's sessions)<br>–Children + Parents: Behavior analog therapy (BAT): Children and<br>parents apply the FAR methodology in everyday contexts (5<br>weekly sessions after 5 wks of children and parent training)
Kable et al[17]<br>RCT	–Inclusion: Children with PAE with significant levels of alcohol-related physical features or with a clinical diagnosis of FAS/pFAS<br>–Parents needed to attend a group workshop on the impact of PAE on neuro-developmental functioning before enrolment<br>–Age: 5–10 y<br>–Recruited: $n = 30$<br><br>GoFAR: –Assigned: $n = 10$; –Completed: $n = 9$; –Analyzed: $n = 9$<br>FACELAND: –Assigned: $n = 10$; –Completed: $n = 9$; –Analyzed: $n = 10$<br>Control: –Assigned: $n = 10$; –Completed: $n = 9$; –Analyzed: $n = 9$	FACELAND:<br>Re-initiation after 8 mo due to family crisis: $n = 1$	GoFAR with 2 components:<br>–Children: Children learn metacognitive control strategies (FAR methodology) through computer games (5 weekly sessions)<br>–Parents: Parents learn about the neurodevelop-mental/behavioral<br>impacts of PAE and how to facilitate the child's behavioral regulation<br>skills (5 weekly 1-h sessions parallel to children's sessions)

 © 2025. Thieme. All rights reserved.

(*Continued*)

Reference study type	Participants (number and characteristics)	Drop-outs	Intervention
Petrenko et al[18]<br>RCT	–Inclusion: FASD diagnosis or confirmed PAE; 4–8 y old; living within a reasonable distance of two New York study sites; expected to remain in their current placement for the study duration (~18 mo, including 9-mo intervention and follow-up time points)<br>–Exclusion: moderate to severe intellectual disabilities (IQ < 55); lacked sufficient English proficiency; severe physical or mental conditions<br>–Age: 4–8 y<br>FoT:<br>–Assigned: $n = 19$<br>–Accepted: $n = 16$<br>–Completed:<br>$n = 15$<br>–Analyzed: $n = 15$<br>Control:<br>–Assigned: $n = 11$<br>–Accepted: $n = 10$<br>–Completed: $n = 9$<br>–Analyzed: $n = 12$ (3 declining treatments were included in the analysis)	FoT:<br>3 families declining treatment (logistical difficulties)	FoT:<br>Children received a neuropsychological and diag-nostic<br>evaluation to promote the protective factor of early diagnos<br>is and to identify the child's neuropsychological profile (Pers<br>onalized feedback to caregivers)<br>FoT includes 2 empirically validated programs:<br>–The preschool/kindergarten Promoting Alternative Thin<br>king Strategies (PATHS) curriculum (Domitrovich et al, 200<br>5): Program in small groups including children with and with<br>out PAE and aiming at preventing violence, ag-gression, and ot<br>her behavioral problems by promoting social com-petence and<br>developing emotional skills. Children learn self-control, emotio<br>nal understanding, positive self-esteem, peer rela-tionships, and<br>interpersonal problem-solving skills<br>–The Families Moving Forward (FMF) Program (Bertrand, 2009):<br>Core sessions for parents in groups aiming at cre-ating a stable<br>home to reduce violence by targeting family-level risk and protective factors
Petrenko et al[19]<br>RCT	–Inclusion: FASD diagnosis or confirmed PAE; 4–8 y old; living within a reasonable distance of two New York study sites; expected to remain in their current placement for the study duration (~18 mo, including 9-mo intervention and follow-up time points)<br>–Exclusion: moderate to severe intellectual disabilities (IQ < 55); lacked sufficient English proficiency; severe physical or mental conditions<br>–Age: 4–8 y<br>FoT:<br>Initial trial:<br>–Assigned: $n = 19$<br>–Accepted: $n = 16$<br>–Completed:<br>$n = 15$<br>–Analyzed: $n = 15$<br>Follow-up:<br>–Completed:<br>$n = 14$<br>–Analyzed: $n = 14$<br>Control:<br>Initial trial:<br>–Assigned: $n = 11$<br>–Accepted: $n = 10$<br>–Completed: $n = 9$<br>–Analyzed: $n = 12$ (3 declining treatments were included in the analysis)<br>Follow-up:<br>–Completed: $n = 10$<br>–Analyzed: $n = 10$	Follow-up:<br>$n = 3$ (change in child placement ($n = 1$); loss of contact ($n = 1$); declining to participate due to time demands of other services ($n = 1$))	FoT:<br>Children received a neuropsychological<br>and diagnostic evaluation to promote the protective factor of early diagnosis and to identify the child's neuropsychological profile (Personalized feedback to caregivers)<br>FoT includes 2 empirically validated programs:<br>–The preschool/kindergarten Promoting Alternative Thinking Strategies (PATHS) curriculum (Domitro-vich<br>et al, 2005): Program in small groups including children<br>with and without PAE and aiming at preventing violence,<br>aggression, and other behavioral problems by pro-moting<br>social competence and developing emotional skills. Children<br>learn self-control, emotional understanding, positive self-esteem, peer relationships, and inter-personal<br>problem-solving skills<br>–The Families Moving Forward (FMF) Program (Bertrand, 2009):<br>Core sessions for parents in groups aiming at cre-ating a<br>stable home to reduce violence by targeting family-level risk and protective factors

(*Continued*)

 © 2025. Thieme. All rights reserved.

(*Continued*)

Reference study type	Participants (number and characteristics)	Drop-outs	Intervention
O'Connor et al[20]<br>RCT	–Inclusion: Composite IQ ≥ 70; English speaking; living with at least 1 custodial parent/guardian; history of PAE<br>–Exclusion: diagnosis of intellectual disability; psychotic disorder, pervasive developmental disorder<br>–Age: 13–18 y<br>–Recruited: $n = 83$<br>–Eligible after screening: $n = 56$<br>–Analyzed: $n = 54$	SUI:<br>Drop-out: $n = 2$ (conflicting obligation)	SUI with 2 components (parallel; each b = 6; weekly 1-h sessions in small groups):<br>–Adolescents: Modified version of an empirically validated procedure.<br>The used strategies are focused on modeling, coaching, behavioral rehearsal,<br>and performance feedback; the intervention incorporated motivational enhancement<br>techniques, normative feedback, education, risk assessment, coping, and alcohol refusal<br>skills training. Participants got a Workbook<br>–Caregivers: Adapted from the NIAAA protocol "Make a Difference: Talk to Your Child About Alcohol". The aim was to empower them in assisting their teens to resist alcohol use.<br>Caregivers got a workbook, as well.
	Project Step up (SUI):<br>–Assigned: $n = 28$<br>–Analyzed: $n = 26$<br>–Abstinent/infrequent drinkers:<br>$n = 15$<br>–Light/moderate drinkers: b = 11<br><br>Control:<br>–Assigned: $n = 28$<br>–Analyzed: $n = 28$<br>–Abstinent/infrequent drinkers: $n = 21$<br>–Light/moderate drinkers: b = 7		
Jirikowic et al[21]<br>CCT (not randomized)	–Inclusion: confirmed PAE; diagnosis of FASD (FAS, SE-AE, or ARND); a previously identified sensorimotor impairment based on clinical diagnostic assessment results<br>–Exclusion: IQ < 60; a severe, co-occurring neuromotor condition that impaired ambulation/independent standing for at least 2 min; a history of serious head injury/seizures; a visual acuity impairment not corrected by glasses; report of any lower limb or back injury within the previous 6 mo; current living in an unstable home placement<br>–Age: 8–15 y	STABLE home:<br>3 children received equipment but never started; 2 started but did not finish due to frustration or dizziness; 1 finished but did not complete postintervention assessments	STABEL:<br>Virtual reality game (STABEL) that facilitates task-specific balance<br>practice under altered sensory conditions (visual, vestibular,<br>somatosensory) by moving on a pliable standing surface.<br>Training consisted of 3 6-min blocks that progressed in difficulty by<br>altering the stability and complexity of the VR visual display.<br>Total of 5 30–35 min sessions over 1 mo.
	University laboratory (STABEL lab):<br>–Enrolled: $n = 6$<br>–Completed: $n = 6$<br>–Analyzed: $n = 6$<br><br>Home (STABEL home):<br>–Enrolled:<br>$n = 15$<br>–Completed:<br>$n = 9$<br>–Analyzed:<br>$n = 9$<br><br>Control:<br>–Enrolled: $n = 8$<br>–Completed: $n = 8$<br>–Analyzed: $n = 8$		STABEL lab:<br>Participants used STABEL in a university laboratory<br><br>STABEL home:<br>Participants used STABEL at home

 © 2025. Thieme. All rights reserved.

(*Continued*)

Reference study type	Participants (number and characteristics)		Drop-outs	Intervention
McCoy et al[22] Uncontrolled intervention study	–Children with FASD: Inclusion criteria: 8–16 y; confirmed PAE; FASD diagnosis; previously identified sensorimotor impairment based on clinical diagnostic assessment results<br>–Exclusion: IQ < 60; severe co-occurring neuromotor condition that impaired ambulation or independent standing for ≥ 2 min; history of serious head injury/seizures; visual acuity impairment not corrected by glasses; report of any lower limb or back injury within the previous 6 mo; currently living in an unstable home placement<br>–Age: 8–16 y<br>–Received: $n = 11$<br>–Completed: $n = 11$<br>–Analyzed: $n = 11$	–Typically developed children (TD):<br>–Inclusion: 8–16 y<br>–Exclusion: identified sensory/motor impairment; current/past special education services; history of serious head injury/seizures; PAE (> 3 reported drinks by mother for the duration of pregnancy); visual acuity impairment not corrected by glasses; report of any lower limb or back injury within the previous 6 mo<br>–Age: 8–16 y Received: $n = 11$<br>–Completed: $n = 11$<br>–Analyzed: $n = 11$	NA	STABEL:<br>Virtual reality game (STABEL) that facilitates task-specific balance practice under altered sensory conditions (visual, vestibular, somatosensory) by moving on a pliable standing surface. Training consisted<br>of 3 6-min blocks that progressed in difficulty by altering the<br>stability and complexity of the VR visual display for a total of 30 min.
Zarnegar et al[23] Uncontrolled intervention study	–Inclusion: age of ≤5 y, in the care of their adopted families for 6 mo, diagnosis of FASD by a medical provider, history of maltreatment or loss, adoptive caregiver(s) who could fully engage in the intervention process during the study time period and who could complete measures in English<br>–Exclusion: taking of psychotropic medications, additional genetic syndrome, active grand-mal epileptic seizures, history of serious head injury, profound intellectual disability<br>–Age: 10–53 mo<br>–Agreed to participate: $n = 38$<br>–Assigned: $n = 15$<br>–Excluded before treatment: $n = 5$ (missed appointments: 3; movement: 2)<br>–Excluded after treatment: $n = 3$ (movement: 1; other familial reasons: 2)<br>–Completed for at least 6 mo: $n = 10$<br>–Analyzed: $n = 10$ children and 20 adoptive parents		NA	The Neurosequential Model of Therapeutics (NMT) Metrics were used to estimate the child's functional capacity. Based on that individual somatosensory interventions<br>were suggested for each child<br>Additionally to somatosensory interventions:<br>–Child-Parent Psychotherapy (CPP): evidence-based,<br>relationship-focused, reflective, and developmentally<br>oriented model of psychotherapy that uses caregivers as the<br>agents of change. Weekly for 6 mo<br>–Mindful Parenting Education (MPE): Parents received psychoeducation<br>regarding FASD, their child's self-regulation, and how to work through their<br>own feelings and emotions while dealing with them. Twice per week for 6 mo.

(*Continued*)

 © 2025. Thieme. All rights reserved.

(*Continued*)

Reference study type	Participants (number and characteristics)	Drop-outs	Intervention
Regehr[24]<br>CCT (not randomized)	–Inclusion: PAE or FASD, 4–10 y old<br>–Exclusion: significant neurological or medical condition that would prevent them from benefiting from the interventions (e.g., autism)<br>–Age: 4–10 y<br>–Enrolled: $n = 29$<br><br>The Social Skills Improvement System Intervention Guide (SSIS-IG):<br>–Assigned: $n = 14$<br>–Completed: $n = 14$<br>–Analyzed: $n = 14$<br><br>MILE:<br>–Assigned: $n = 15$<br>–Completed: $n = 15$<br>–Analyzed: $n = 14$	NA	SSIS-IG:<br>–Individual program that focusses on instruction, modeling, rehearsal, and performance feedback on social<br>skills difficulties and problem behaviors<br>–One-on-one instruction<br>–30 min. Sessions, 1–2 times a week over 5–7 wks (total of 5 h)
O'Connor et al[25]<br>CCT (not randomized)	–Families were required to complete 2 intake sessions with a Child and Family Guidance Centre clinician (assessment and treatment planning session)<br>–Inclusion criteria for children: 6–12 y of age; IQ $\geq$ 70; English speaking; living with at least 1 custodial parent or guardian; with/without PAE<br>–Inclusion criteria for parents: English or Spanish-speaking<br>–Exclusion criteria for children: major sensory or motor deficits; past diagnosis of intellectual disability, psychotic disorder, pervasive developmental disorder<br>–Age: 6–12 y<br>–Recruited: 85 children (with PAE = 32; without PAE = 53)<br><br>Children's Friendship Training (CFT):<br>–Assigned: $n = 41$<br>–Received: $n = 32$<br>–Analyzed posttreatment: $n = 32$<br>–Analyzed using multiple imputations: $n = 41$<br><br>Standard of care (SOC):<br>–Assigned: $n = 44$<br>–Received: $n = 35$<br>–Analyzed posttreatment: $n = 35$<br>–Analyzed using multiple imputations: $n = 44$	CFT<br>$n = 9$<br>Reasons for not receiving intervention: child illness, family circumstances, child unsafe, unknown<br><br>SOC:<br>$n = 9$<br>Reasons for not receiving intervention: child illness, family circumstances, child unsafe, unknown	Modified CFT with 2 components:<br>–Children training in group settings to emphasize the child's<br>friendship skills. It is tailored to the neurodevelopmental needs of<br>children with FASD. Social skills were taught using instruction on simple rules<br>of social behavior, modeling, behavioral rehearsal, and performance feedback<br>through coaching during treatment sessions. 12 90-min sessions over the<br>course of 12 wks<br>–Parents training in separate concurrent sessions in group settings to learn<br>the key skills being taught to their children. They were taught how to facilitate social<br>competence in their children by arranging play dates, facilitating the completion of<br>weekly homework assignments, and providing in vivo social coaching. Handouts<br>outlining the skills being taught to children are distributed to parents.<br>12 90-min sessions over the course of 12 wks.
Leenaars et al[26]<br>Retrospective cohort study	–Inclusion: closed case files of families for which at least one post needs or goals measure was available; families with a child with FASD (confirmed FASD diagnosis; children possibly having FASD, but<br>–maternal drinking was not confirmed, children were suspected of having FASD, but had not yet been assessed)<br>–Age: 1–23 y<br>–Analyzed: $n = 186$ families	NA	Coaching Families Program (CF) is a family goal-based<br>mentoring program on an individual level. Mentors educate families about FASD, help them access resources, and engage them in successful advocacy.
Graham et al[27]<br>Intervention study	–Inclusion: English as primary language, 8–12 y<br>–Exclusion: other known causes of mental deficiency, adopted from abroad after the age of 5, head injury involving loss of consciousness, physical or psychiatric conditions that prevented involvement<br>–Exclusion for analyses: accuracy $<$ 80% in Flanker task; being an extreme outlier (at least 3 SD from group mean) across RT and accuracy in Flanker task<br>–Age: 8–12 y	NA	Modified flanker task including reward (positive reinforcement) and response cost (negative punishment):<br>4 blocks of 96 trials (total of 25 min) were presented varying by flanker type:<br>–Congruent<br>–Incongruent

 © 2025. Thieme. All rights reserved.

(*Continued*)

Reference study type	Participants (number and characteristics)			Drop-outs	Intervention	
	Alcohol-exposed (AE):<br>–Inclusion: heavy PAE<br>–exclusion: other known causes of mental deficiency, adopted from abroad after the age of 5, head injury involving loss of consciousness, physical or psychiatric conditions that prevented involvement<br>–Analyzed: $n = 34$<br>–Analyzed with ADHD symptoms: $n = 29$	idiopathic ADHD (ADHD):<br>–inclusion criteria: ADHD<br>–exclusion: greater than minimal PAE (average exposure < 1 drink per week and no more than 2 drinks per occasion)<br>–Analyzed: $n = 23$	Controls (CON):<br>–exclusion: indicators of ADHD; subclinical symptoms of ADHD on the C-DISC-4.0<br>–Analyzed: $n = 31$		–Neutral<br>–Single<br>And reinforcement condition:<br>–No Reward or Response Cost (NR)<br>–Reward Only (REW)<br>–Reward + Occasional Response Cost (ROR)<br>–Equal Probability of Reward and Response Cost (EQ)<br>Points were earned or lost based on speed and accuracy and were shown on screen (feedback and extrinsic motivation). Prize corresponds to the points at the end.	
Kable et al[28]<br>RCT	–Recruited from a multidisciplinary FAS diagnostic clinic<br>–Inclusion for children: clinical diagnosis of FAS or pFAS (IOM Criteria) or significant levels of alcohol-related dysmorphology (standard pediatric dysmorphia checklist)<br>–Inclusion for adults: parents or caregivers of children<br>–Mean age of participating children: 6–7 y			$n = 23$	Workshop group:<br>–2 d workshop in-person (each 2h)<br>–Education about FASD, information on effective behavior management strategies, and advocacy tools	Internet group:<br>–Web-based workshops<br>–Education about FASD, information on effective behavior management strategies, and advocacy tools
	Workshop group:<br>–Recruited: $n = 29$<br>–Analyzed: $n = 23$	Standard Information group:<br>–Recruited: $n = 24$<br>–Analyzed: $n = 18$	Internet group:<br>–Recruited: $n = 29$<br>–Analyzed: $n = 18$			

 © 2025. Thieme. All rights reserved.

Control	Outcomes	Results	Comments	Risk of bias
NA	–Tolerability (depending on polymorphisms)<br>–Severity of ADHD symptoms (depending on polymorphisms)	Tolerability: 104 successfully treated: 3 without improvement, 7 discontinued due to adverse effects (occurred at the time of drug introduction and decreased after the introduction of a modified form of MPH); no cardiotoxic effects or life-threatening symptoms; Borderline significance between adverse effects and the COMT rs4680 minor allele (G > A; $p < 0.049$)<br>Severity of ADHD:<br>–All children: treatment was effective in > 90% of children<br>–Children with morphological features of FASD: significant reduction in symptoms of hyperactivity and impulsivity ($p < 0.0001$); no improvement in attention deficits ($p = 0.2024$)<br>–Children without morphological features of FASD: significant improvement in attention ($p < 0.001$); reduction in hyperactivity ($p = 0.0163$); no significant reduction in impulsivity ($p = 0.1274$)<br>No association of the studied polymorphisms: DRD2 rs1076560: C > A or DRD2 rs1800497: G > A with the efficacy or safety of MPH	–No specific age range<br>–Missing source of recruitment and recruitment process<br>–Very short treatment period<br>–No international diagnostic valid instrument for the severity questionnaire<br>–Genomic DNA was extracted and genotyping for COMT rs4680, DRD2 rs1076560, and rs1800497 SNPs was used<br>–No monitoring of adherence<br>–Children with morphological features of FASD had significantly higher doses of MPH<br>–No subanalyses of sex, medicine	Low for effectiveness<br>Moderate for adverse effects<br>(ROBINS-I modified
Placebo:<br>–Equivalent doses of an oral inactive placebo treatment<br>–Daily doses for 6 wks	–Neuropsychological measures of memory, executive function, attention and hyperactivity<br>–Association between treatment compliance/dietary choline intake and outcomes<br>–Tolerability	Cognitive performance:<br>–Choline group did not differentially improve in any cognitive performance domain (no group or group x time interaction)<br>–Treatment compliance and mean dietary choline intake were not predictive of cognitive performance<br>–No significant interaction of group x time x age group in any cognitive outcome variable<br>Compliance: high treatment compliance in both groups (about 96%)<br>Tolerability and adverse events:<br>–Significantly more children in the choline group reported at least 1 adverse event<br>–No serious adverse events	–Very small sample size in subgroup analyses of age<br>–Intention-to-treat analyses (subanalyses of children completing the study did not change results)<br>–Children without FASD diagnosis included	Low<br>(RoB-2)
Placebo:<br>–Equivalent doses of an oral inactive placebo treatment<br>–Daily doses for 9 mo	–Feasibility of parental administration<br>–Tolerability<br>–Serum choline levels	Feasibility:<br>–Compliance: 82–87%<br>–No evidence for dietary confounding<br>Tolerability:<br>–Minimal adverse effects: no group differences on all adverse events except for a fishy body odor in the choline group ($p = 0.011$)<br>–In both groups: taste problems at least once (55%); nonstandard administration at least once (75%)<br>Serum choline level:<br>–Choline group increased choline at all time points: 1 mo ($p = 0.004$), 6 mo ($p < 0.001$), and 9 mo ($p < 0.001$)<br>–Choline group increased betaine concentration at all time points: 1 mo ($p = 0.04$), 6 mo ($p = 0.03$), and 9 mo ($p = 0.04$)<br>–No changes in phosphatidycholine<br>–Choline group had higher sphingomyelin concentrations at baseline ($p = 0.04$) and months 1 ($p = 0.05$), but no differences in months 6 ($p = 0.25$) and 9 ($p = 0.91$)	–No child living with biological parents<br>–Prenatal drug use was suspected with alcohol being the dominant substance ($n = 14$)<br>–Reasons for drop-out: not related to the study; lost to follow-up; refused dose after one time trying it<br>–Potential unblinding due to fishy body odor<br>–Not all children at each testing point	Low (RoB-2)

 © 2025. Thieme. All rights reserved.

(*Continued*)

Control	Outcomes	Results	Comments	Risk of bias
Placebo:<br>–Equivalent doses of an oral inactive placebo treatment<br>–Daily doses for 9 mo	–Neurocognitive functioning (particularly hippocampal-dependent memory)<br>–Feasibility<br>–Serum choline levels<br>–Tolerability	Global cognitive functioning: no main effects of treatment and no interaction effect<br>The Mullen Early Learning Composite was correlated (with age controlled for) with EI delayed performance for items (partial $r = 0.56$, $p < 0.001$) and ordered pairs (partial $r = 0.47$, $p < 0.001$) at baseline but not at the 9-mo visit ($p > 0.17$ for all)<br>Hippocampus-dependent long-term memory:<br>–No significant main effects of treatment on EI delayed memory performance<br>–race and FASD diagnosis as no moderators<br>–Age as a moderator:<br>◦ subanalysis: splitting participants into a younger group consisting of 2.5 to ≤ 4.0-y-olds ($n = 30$; placebo: $n = 13$; choline: $n = 17$) and an older group consisting of > 4.0–5.0-y-olds ($n = 30$; placebo: $n = 16$; choline: $n = 14$)<br>◦ Largest improvement in delayed EI performance in the young choline group<br>–For items: *t*-test [t(28) = −2.41, $p = 0.023$]; d = 0.54 young choline group showed an increase of 21% compared with 7% in the young placebo group<br>–For ordered pairs: [t(28) = −2.18, $p = 0.038$]; d = 0.50 young choline group showed an increase of 28% compared with 16% in the young placebo group<br>◦ No significant differences for the older age groups<br>Feasibility:<br>–Compliance: dose on 88% of days<br>–Diet: no group differences regarding compliance or dietary changes<br>Serum choline levels:<br>Significant increase in serum choline (102%; $p < 0.0001$) and betaine (106%; $p < 0.0001$) in choline group<br>Tolerability: fishy body odor is the only adverse event	–All children received the same dosage regardless of their weight<br>–Intention-to-treat analysis<br>–Due to different group distributions age, race, and FASD diagnosis were included as covariates<br>–Immediate recall performance showed no improvement in the choline group for items; but for ordered pairs, the choline group performed worse than the placebo<br>–Improvements in delayed memory in the young group were only present after controlling for immediate recall performance<br>–Potential ceiling effect in EI: young choline group had slightly lower delayed EI performance than the other groups at baseline	Low (RoB-2)
Placebo:<br>–Equivalent doses of an oral inactive placebo treatment<br>–Daily doses for 9 mo	Potential long-term cognitive and behavioral implications (intelligence, memory, executive functioning, and behavior)	General cognitive functioning:<br>–Choline group had higher nonverbal IQ (8% difference; (F1, 28) = 5.17; $p = 0.03$; ηp2 = 0.17); and higher working memory scores (11.7% difference; F (1, 28) = 7.74; $p = 0.01$; ηp2 = 0.23)<br>–Components of nonverbal IQ: significant group effects in 2 of 5 components: nonverbal Visual-Spatial Reasoning with Choline group showing better performance (28.9% difference; F(1, 29) = 9.93; $p = 0.004$), and nonverbal Working Memory with Choline group showing better performance (26.8% difference; F(1, 29) = 6.37; $p = 0.018$)<br>–No significant differences in Verbal IQ; Fluid Reasoning; Knowledge; Quantitative Reasoning; Visual-Spatial Processing and Full-Scale IQ<br>Memory functioning:<br>No significant group differences regarding the EI paradigm; age was not a significant modulator;<br>In the NEPSY-II choline group scored significantly higher in Memory for Names Delayed (37.9% difference; $p = 0.04$; d = 0.77)<br>Executive functioning:<br>No group differences in the Dimensional Change Card Sort Test; but a trend toward higher performance in the Flanker Inhibitory Control Test in the choline group compared to placebo (13.5% difference; $p = 0.08$; d =0.66)	–No measures of serum choline level<br>–Dietary intake as a potential mediator	Low (RoB-2)

(*Continued*)

 © 2025. Thieme. All rights reserved.

(*Continued*)

Control	Outcomes	Results	Comments	Risk of bias
		Behavioral and emotional functioning:<br>Choline group had significantly lower scores in the parent-reported scale for ADHD problems (estimated marginal mean = 62.1; SE = 2.1) compared to placebo group (estimated marginal mean = 69.0; SD = 2.0; 10.5% difference; F (1,28) = 5.57; $p = 0.026$; ηp2 = 0.17)		
Placebo:<br>–Equivalent doses of an oral inactive placebo treatment<br>–Daily doses for 9 mo	Correlation between choline-related SNPs and memory and cognition (at study terminus, and 4 y follow-up)	14 SNPs within the choline transporter gene SLC44A1 were significantly associated with the change score (pre-/post) on an EI sequential memory task ($p = 0.04969$)<br>Same 14 SNPs + 2 SNPs within SLC44A1 were associated with change scores for adjacent pairs of items from the sequence ($p = 0.023$)<br>Only participants in the choline group who had these variants were more likely to show improvement in the memory task (pre-/post)<br>Some SNPs were associated with improved performance in the working memory measure of the Stanford-Binet Intelligence Scale, version 5, at 4 y follow-up, in the EI immediate memory task at baseline, in the NIH Toolbox Dimensional Card Sort Test at 4 y follow-up, and change-score measures from baseline to 9 mo for the Immediate Memory Task in the EI	Small number of participants with specific SNPs	Low (RoB-2)
Sham:<br>2 parallel components:<br>–Cognitive training: 5 tasks from BrainHQ focussing on working memory and attention. Tasks were completed 4 times (total of 46 min) during each of 5 weekly sessions<br>–Sham: transcranial stimulation ramped up to 2mA over the course of 30 s, ramped down to 0mA over 30 s and remained at 0mA.	–Feasibility<br>–Tolerability<br>–Cognitive gains (near/far transfer)	Tolerability:<br>No significant differences in tDCS-related side effects between the groups and no serious adverse events<br>Near transfer of cognitive gains:<br>–For visuospatial working memory, a significant effect of time was observed (F (1, 144) = 2.46, $p = 0.047$), with both groups showing improvement over the visits, but no significant effect for tDCS versus sham (F(1, 39) = 0.017, $p = 0.911$) or an interaction effect (F(1, 144) = 4.41, $p = 0.612$). No meaningful between-group effect size<br>–In the continuous performance test tDCS performed significantly better over time than sham (F(1, 39) = 4.31, $p = 0.043$). No significant overall effect of time (F(1, 144 = 1.36, $p = 0.247$) or an interaction (F(1, 144) = 1.46, $p = 0.221$). Posthoc contrast analyses: significant tDCS versus sham differences at visit 3 ($p = 0.033$), visit 4 ($p = 0.043$) and visit 5 ($p = 0.046$). Medium between-group effect size (d = 0.64)<br>Far transfer of cognitive gains:<br>–For the verbal fluency test, no significant effects of tDCS were seen for either letter VF (F(1, 36) = 0.067, $p = 0.797$), or category verbal fluency (F(1, 36) = 0.049, $p = 0.826$)<br>–No treatment effect was seen for the trail-making test performance for number sequencing (F(1, 36) = 0.064, $p = 0.801$), letter sequencing (F(1, 36) = 2.75, $p = 0.107$), nor combined letter and number sequencing (F1(1, 36) = 0.197, $p = 0.659$).	–Main effect of treatment was only marginally significant and would likely not remain significant after correction for multiple comparisons<br>–In cognitive training attention was emphasized and working memory was only trained in 2 tasks<br>–Effects of more training sessions unclear	Low (RoB-2)
TAU:<br>Pharmacological treatment as usual	–Social skills<br>–Internalized symptomatology<br>–Externalized symptomatology<br>–Severity of FASD symptoms	Social Skills:<br>–A main effect on time [F(1.30) = 15.54, $p = 0.001$] and an interaction time x group with the DAT group being the one who improved more [F(1.30) = 13.82, $p = 0.02$, d = 0.8]<br>–Problem behavior: no interaction of time x group<br>Internalizing symptoms:<br>Main effect of time [F(1.30) = 10.45, $p = 0.001$], but there was no significant interaction of time x group<br>Externalizing symptoms:<br>Main effect of time [F(1.30) = 12.35, $p = 0.001$] and also a significant interaction on time x group [F(1.30) = 11.59, $p = 0.03$, d = 0.56<br>Severity of FASD Symptoms:	–Low power<br>–All participants had ADHD<br>–Effects of DAT only with pharmacological treatment<br>–Maintenance of the results is unclear<br>–No definition of TAU<br>–No clear description of DAT<br>–Results might be due	Moderate (RoB-2)

 © 2025. Thieme. All rights reserved.

(*Continued*)

Control	Outcomes	Results	Comments	Risk of bias
		Main effect of time [F(1.30) = 12.549, $p = 0.001$] and also a main effect on time x group interaction with FASD severity decreasing significantly more in the DAT group [F(1.30) = 16.54, $p = 0.001$, d = 0.5].	to the intensive treatment sessions and not due to the dogs involved<br>–Big range of age (6–18 y)	
NA	–Everyday problem behavior and attention skills<br>–Emotional and behavioral strengths<br>–Utility and feasibility<br>–Attention<br>–Working Memory<br>–Academic skills<br>–Children's response to training	–Everyday problem behavior and attention skills: BRIEF and CRS-3 could not be analyzed due to very low questionnaire return rates<br>–Emotional and behavioral strength: BERS-2 could not be analyzed due to very low questionnaire return rates<br>–Utility and feasibility: 80% reported easy incorporation into school schedule<br>–Attention: sign. reductions in total errors on the KiTAP for distractibility ($p = 0.002$, d = 0.87) and divided ($p = 0.001$, d = 0.91) tasks, and no significant reduction of total errors in the flexibility task ($p = 0.226$, d = 0.31); no differences on the total correct responses of any KiTAP task<br>–Working Memory: significant improvement in the Listening Recall ($p = 0.003$, d = 0.45) and Counting Recall ($p = 0.001$, d = 0.61) verbal working memory tasks from WMTB-C; no significant changes on the WISC-IV verbal and spatial span tasks<br>–Academic skills (AIMSweb): significant reductions in errors on the oral reading fluency task ($p = 0.002$, d = 1.30); the total number of correct words did not change (children read less quickly, but the read words were more likely to be correct)<br>–Academic skills (interview with Educational Assistants): spelling, reading, and math were ameliorated (no quantitative testing)<br>–Children's response to training (interview with Educational Assistants): functional improvements in the classroom (improved focus and alertness, decreased hyperactivity, less resistance to engaging in new/challenging activities, increased academic engagement and mastery); emotional and social improvements	–Potential bias due to additional support services<br>–Possible practice effect<br>–No sub-analysis of disorder type<br>–No manualized version of the Caribbean Quest intervention protocol that includes evidence-based guidelines to assist in metacognitive training<br>–Impact of components of intervention unclear (serious game vs metacognitive training)	Moderate (ROB-INS-I modified)
Parents Instruction:<br>Parents completed workshops and received a manual discussing math learning in children with FASD and strategies for facilitating math learning at home	–Instructor satisfaction<br>–Instructor knowledge<br>–Instructor fidelity<br>–Child's academic outcomes<br>–Parent satisfaction	Instructor satisfaction, knowledge, and fidelity:<br>–High satisfaction and willingness to recommendation<br>–Significant group effect on the knowledge scores (F(2, 48) = 8.21, $p < 0.001$, ηp2 = 0.255) with center-based employees who were not trained in the MILE program receiving lower scores (X = 8.00, STD = 1.92) than both instructors trained at the center (X = 9.83, STD = 0.76) or in the community (X = 9.39, STD = 0.41), but no difference between center-instructors and community-instructors<br>–The session number was positively related to the fidelity score ($r = 0.36$, p 0.005), suggesting that instructors were improving over the course of sessions. A	–Original treatment plan of 6 wks was extended to 15 wks<br>–Detailed instruction training with feedback on the sessions and mock sessions<br>–Children were evaluated by a psychologist or psychology trainee blind to group status<br>–Possible impact of maturation effects as groups differed in days of completion<br>–KeyMath only administered to children ≥ 5 y (n's: Centre = 9; Community = 14; Parent Instruction = 12)<br>–No results for the instrument adapted from math concepts	Moderate (RoB-2)

(*Continued*)

 © 2025. Thieme. All rights reserved.

(*Continued*)

Control	Outcomes	Results	Comments	Risk of bias
		significant effect was found for block (F(2, 52) = 4.26, $p < 0.019$) but was not found for the site. Higher ratings of fidelity were obtained in the final block of five sessions relative to the initial block of sessions<br>Child's academic outcomes:<br>–No significant group*time effect on the individual tests<br>–Using the math summary score from summing the raw scores from Bracken, TEMA, and Handwriting measure: significant time*group effect with MILE groups demonstrating more positive gains in math skills than Parent Instruction group (F(2, 41) = 3.4, $p < 0.04$, ηp2 = 0.139)<br>–Within the MILE groups, fidelity ratings were significantly positively correlated with change in the total score of KeyMath (standard score D: $r = 0.48$, $p < 0.02$) and the TEMA (raw score D: $r = 0.35$, $p < 0.04$; standard score D: $r = 0.45$, $p < 0.04$), but no significant correlations with the raw scores of KeyMath, the number of writing score or the total scores from Bracken<br>Parent satisfaction:<br>–Compared to MILES groups, the Parent Instruction group reported less agreement that the child improved in math skills ($p < 0.001$) and that their ability to help the child to study had improved ($p < 0.01$)<br>–Centre-MILES group reported a more favorable rating than the Parent Instruction group on: informative ($p < 0.05$), helpful ($p < 0.05$), improved understanding of FAS/pFAS ($p < 0.01$), and helped child's study habits ($p < 0.05$);<br>–Community-MILE group and Parent Instruction group did not differ significantly	administered as part of the Bayley Scales of Infant Development 2nd Edition (for children < 5 y)<br>–Raw and standardized scores were analyzed<br>–Broad age span (possible floor effects with younger children)	
SSIS:<br>–The Social Skills Improvement System Intervention:	–Mathematical skills<br>–Executive functioning	Mathematical skills:<br>–MILE group improved significantly more on total KeyMath score from pre-to posttesting compared to the contrast group (F(1, 27) = 5.89, $p < 0.05$, η2 = 0.19);	–Posttesting by a blinded research assistant<br>–Working Memory	Moderate (ROB-INS-I)

 © 2025. Thieme. All rights reserved.

(*Continued*)

Control	Outcomes	Results	Comments	Risk of bias
Program focussing on social skills<br>–One-on-one individualized tutorial sessions Weekly home assignments<br>–10–30-min sessions once/twice a week for 6–8 wks	–Working memory<br>–Visuospatial functioning<br>–Influence of participant's characteristics	–MILE group gained significantly more raw points on the Basic Concepts composite than the contrast group ($F(1, 27) = 4.98$, $p < 0.05$, $\eta 2 = 0.16$) but the overall MANOVA of the 5 subtests of the Basic Concepts composite was not significant ($F(5, 27) = 2.01$, $p > 0.05$);<br>–MILE group did not gain significantly more points on Operations and Problem Solving than the contrast group;<br>–MILE group showed greater increases in total math achievement than the control group from pre-test to 6-mo follow-up ($F(1, 18) = 5.47$, $p < 0.05$, $\eta 2 = 0.24$)<br>Executive functioning:<br>No significant differences in raw scores on the Auditory Attention and Response set, but the trend: MILE group had larger gains in total correct in Auditory Attention ($p = 0.18$, total correct in Response ($p = 0.13$), and in Omission errors in Response ($p = 0.13$) compared to control group<br>Working Memory: No significant treatment effect<br>Visuospatial functioning: No significant treatment effect<br>Influence of participant's characteristics:<br>–Within the MILE group: Older age was associated with higher KeyMath Total and Operations raw change scores. PAE 'diagnosis' was strongly associated with greater raw point gains in Operations, Problem Solving, and Total Score. A lower Verbal IQ was associated with greater change in KeyMath Operations and Problem-Solving raw scores. A strong negative relationship was observed between overall IQ and KeyMath Problem Solving raw change score ($r(13) = -0.54$, $p < 0.05$). Sex was not significantly related to the KeyMath Total raw change score. SES was not significantly correlated with changes in math achievement<br>–Within the SSIS group: PAE 'diagnosis' was not associated with greater raw point gains in Operations, Problem Solving, and Total Score. A higher Verbal and Visual IQ was associated with more raw changes in problem-solving. IQ was strongly positively related to the KeyMath Problem-Solving raw change score ($r(11) = 0.85$, $p < 0.01$). Sex was not significantly related to the KeyMath Total raw change score. SES was not significantly correlated with changes in math achievement.	Test Battery for Children suitable for children aged 5–15 y<br>–No assessment of executive functioning, working memory, and visuospatial functioning at 6-mo follow-up<br>–N at follow-up: 19<br>–Possible effect of control intervention on outcomes<br>–Possible practice effect<br>–MILE group had lower Math scores at the pretest (greater improvement potential)	
Control:<br>–Parents received feedback and recommendations regarding the child's behavior, learning, and emotional functioning<br>–No further intervention	–Executive functioning<br>–Emotional and social problem-solving skills	Executive functioning:<br>–Significant interaction between group and time, $F(8, 57) = 3.09$, $p = 0.006$, $\eta p2 = 0.30$); significant main effect for group, $F(8, 57) = 2.61$, $p = 0.02$ with treatment group showing more improvement; nonsignificant main effect for time, $F(8, 57) = 1.93$, $p = 0.07$<br>–No specific subtest was responsible for the significant effect, but the combination of the subtests<br>Emotional problem solving:<br>–Significant interaction between group and time, $F(7, 52) = 2.92$, $p = 0.012$, $\eta p2 = 0.28$; significant main effect for group, $F(7, 52) = 3.54$, $p = 0.003$ with treatment group showing more improvement; and significant main effect of time, $F(7, 52) = 492.88$, $p < 0.001$<br>–Specific subtest was responsible for significant effect: treatment group did not rely on easy or unrealistic solutions to problems	–Only children living with foster or adoptive caregivers<br>–No predetermined allocation sequence for randomization, but randomization through random numbers<br>–Transformation of the data to eliminate skewness by extreme outliers<br>–Outcome differences might be muted by the extensive feedback and comprehensive recommendations the assessment psychologist provided to all	Moderate (RoB-2)

(*Continued*)

 © 2025. Thieme. All rights reserved.

(*Continued*)

Control	Outcomes	Results	Comments	Risk of bias
DTC:<br>Waiting list	–Cognitive executive functioning<br>–Socio-affective executive functioning<br>–Emotional/behavioral functioning<br>–Social skills	–Significant improvements of TXT compared to DTC in inhabitation naming (F(2, 20) = 6.12, $p = 0.001$, effect size = 0.283) with scores changing into the normal range<br>–No significant changes in Inhibition-Inhibition score (F(2, 18) = 3.27, $p = 0.15$, effect size = 0.060) or Inhibition-Switching (F(2, 18) = 2.12, $p = 0.30$, effect size = 0.010) in TXT compared to DTC<br>For attention, trend-level effect for the TEA-Ch Score (F(2, 22) = 2.89, $p = 0.15$; effect size = 0.047)<br>–No group differences in attention switching or planning from CANTAB<br>–Significant treatment effect for NEPSY Affect recognition (F(2, 21) = 4.82, $p = 0.05$, effect size = 0.103) with scores improving into the normal in TXT<br>–For social cognition, trend level effect for Strategic Control of Emotions (F(2, 21) = 6.49, $p = 0.07$, effect size = 0.004) with TXT showing improvement and Personalized Emotions (F(2, 21) = 5.46, $p = 0.09$, effect size = 0.002) with DTC showing improvements<br>–Sign. treatment effect in behavioral regulation (F(2, 21) = 22.6, $p = 0.01$, effect size = 0.189) and trend-level in General Executive Functioning (F(2, 21) = 21.7, $p = 0.06$, effect size = 0.103) with TXT showing improvements<br>–Sign. treatment effect for Emotional control (F(2, 21) = 4.29 , $p = 0.03$, effect size = 0.170) with TXT showing improvements<br>–Trend-level for Inhibition control (F(2, 21) = 1.96, $p = 0.09$, effect size =0 .085) and CBCL Externalizing Problems (F(2, 21) = 34.6, $p = 0.08$, effect size = 0.095) with TXT showing improvements<br>–No treatment effects were observed for the CBCL Total Behavior Problems or SSIS Social Skills scores<br>–Results from parent-questionnaire data obtained at 6-mo follow-up in nine TXT cases revealed that treatment effects observed at the first posttest were sustained after 6 mo, while an improvement on the Inhibit subscale of the BRIEF was also noted (M posttest (SD): 78.9 (8.7); M follow-up (SD) 74.6 (10.6); $p = 0.01$)	–Different tests for similar outcomes<br>–did not reach significance<br>–Differences in the groups regarding ADHD diagnosis, and alcohol and secondary drugs<br>–No correction for comorbidities<br>–Child with IQ = 70 did not master the third stage	Moderate (ROBINS-I)
DTC:<br>waiting list	CT:<br>no intervention	–Emotion regulation<br>–Inhibition<br>–Brain structure and<br>–function<br><br>Emotion regulation:<br>BRIEF: significant group*time interaction with TXT having the largest improvement ($p = 0.04$; TXT > CT > DTC)<br>Inhibition:<br>NEPSY-II: sign. group*time interaction in inhibition subscale with improvements in TXT and CT ($p = 0.01$; CT, TX > DTC)<br>Brain structure and function:<br>MRI:<br>–While controlling for multiple comparisons: no significant changes among groups<br>–Uncorrected data: Significant increase in grey matter volumes in some brain regions (e. g. related to self-regulation) in TXT compared to DTC (p between < 0.0001 and 0.005)<br>–Increase in grey matter volume in TXT, DTC, and CT in different areas (p between 0.0001 and	–2 children without FASD diagnosis in TXT<br>–Only 1 child with FAS in DTC and no child in TXT<br>–1 family in TXT was reassignment to DTC after pretest<br>–No between and within group differences with false discovery rate applied (only uncorrected)<br>–Large number of comparisons (possible false-positive/type 1 errors)<br>–Group differences in time between pre– and posttesting (TXT > DTC > CT)<br>–More females in DTC than TXT (differences in	High (ROBINS-I)

 © 2025. Thieme. All rights reserved.

(*Continued*)

Control	Outcomes	Results		Comments	Risk of bias
			0.001)<br>–TXT still differed from the CT group regarding neuroanatomy after treatment	neurodevelopmental peaks)<br>–Normal brain changes during this age<br>–No examination of structure-function correlations on DTC and CT (placebo effect)<br>–Mask was relatively large and allowed for a large number of voxel comparisons	
FACELAND with 3 components:<br>–Children: Children learn to identify emotions through a computer game (5 weekly sessions)<br>–Parents: Parents learn about the neurodevelopmental/behavioral impacts of PAE and how to facilitate the child's behavioral regulation skills (5 weekly 1-h sessions parallel to children's sessions)<br>–Children + Parents: Behavior analogue therapy (BAT): Children and parents apply the FAR methodology in everyday contexts (5 weekly sessions after 5 wks of children and parent training)	Control:<br>no intervention	Disruptive behavior	–No overall time point effect, F(5, 90) < 1, but a significant treatment group*time point effect on the disruptive behavior composite, F(4, 36) = 2.903, $p < 0.035$, ηp2 = 0.244<br>–No time point differences in the Control<br>–GoFAR had significant improvements in disruptive behavior at Mid-Treatment after the Game Learning<br>–FACELAND had significant improvements in disruptive behavior at Posttreatment, after completing the BAT sessions<br>–Intent to treat analysis: trend for the treatment group*time point interaction, F(4, 42) = 1.823, $p < 0.142$, ηp2 = 0.148	–Maintenance of the behavioral change is unknown<br>–Generalisability to other situations is unknown<br>–Parents and observers were not blinded	–Moderate<br>–(RoB-2)
FACELAND with 3 components<br>–Children: Children learn to identify emotions through a computer game (5	Control:<br>no intervention	–Neurocognition<br>–Adaptive functioning<br>–Behavior<br>–Parents' satisfaction and fidelity to	Neurocognition (attention regulation):<br>–Only GoFAR showed significant improvements in a summary score of TOVA = Attention Performance Index (API) that measures efficiency in sustaining	–Highly motivated parents<br>–Children with intellectual disability (IQ < 60) could not complete the neurocognitive measures; others	Moderate (RoB-2)

(*Continued*)

 © 2025. Thieme. All rights reserved.

(*Continued*)

Control	Outcomes	Results		Comments	Risk of bias
weekly sessions)<br>–Parents: Parents learn about the neurodevelopmental/behavioral impacts of PAE and how to facilitate the child's behavioral regulation skills (5 weekly 1-h sessions parallel to children's sessions)<br>–Children + Parents: Behavior analogue therapy (BAT): Children and parents apply the FAR methodology in everyday contexts (5 weekly sessions after 5 wks of children and parent training)		the treatment protocol	attention and inhibiting impulsive responding. On this measure, children who received the GoFAR intervention showed significant improvement at the Posttest while the other two groups did not (Wald χ (2)= 6.09, $p < 0.05$)<br>–Control group performed significantly better in NEPSY-Auditory Attention-SS than the intervention groups<br>Adaptive functioning:<br>FACELAND and GoFAR had significant improvements in the Vineland Daily Living Skills, Domestic subscale that reflects adaptive functioning in the home (in contrast to control; Wald χ (1) =5.39, $p < 0.02$)<br>Behavior:<br>On the CBQ, which measures Temperamental Functioning, Fear, one of the elements of Negative Affect was significantly reduced both when the three groups were compared (Wald χ (2) = 8.59, $p < 0.01$) and when both intervention groups were combined (Wald χ (1) = 7.91, $p < 0.005$)<br>Fidelity:<br>–Significant improvements in parent fidelity in carrying out the FAR methodology in FACELAND and GoFAR group (F(4, 13) = 8.0, $p < 0.002$, eta2 = 0.71)<br>–Parent thought the program was helpful and would recommend it.	completed only some sessions<br>–Parents were not blinded (VABS, CBQ)<br>–No bias with TOVA (computerized measures)<br>–Both intervention groups had higher TOVA API at baseline compared to controls (possible ceiling effect)	
FACELAND with 2 components<br>–Children: Children learn to identify emotions through a computer game (5 weekly sessions)<br>–Parents: Parents learn about the neurodevelopmental/behavioral impacts of PAE and how to facilitate the child's behavioral regulation	Control:<br>no intervention	–Impact of parental engagement in the learning program on child's self-regulation skills<br>–Disruptive behavior	Impact of parental engagement in the learning program on child's self-regulation skills:<br>–Child's ability to regulate attention was significantly related to therapist's ratings of achievement of therapy goals ($r = -0.70$, $p < 0.001$)<br>–Trend: Child's ability to regulate attention was correlated with parental completion of homework ($r = -0.44$, $p = 0.059$)<br>–Trend between therapist's ratings of parent's achievement of therapy goals and reduction in children's destructive behavior	–Highly motivated parents<br>–Parents were not blinded (parents questionnaire for disruptive behavior)	Moderate (RoB-2)

 © 2025. Thieme. All rights reserved.

(*Continued*)

Control	Outcomes	Results	Comments	Risk of bias
skills (5 weekly 1-h sessions parallel to children's sessions)		($r = 0.39$, $p = 0.10$)<br>Disruptive behavior:<br>–No significant multivariate group effect, $F(12, 42) = 1.58$, $p = 0.134$, $\eta 2 = 0.311$<br>–Trend for a specific univariate effect on change in sustained mental effort, $F(2, 25) = 2.77$, $p = 0.08$, $\eta 2 = 0.181$<br>–GoFAR had a significant reduction in frustration level relative to individuals in FACELAND and Controls, $p = 0.05$, and a trend was found for those in GoFAR making more improvement in sustained mental effort, $p = 0.09$. Contrasts between those in FACELAND and Controls were not significant<br>–GoFAR demonstrated greater reductions in disruptive behavioral outcomes than FACELAND on change in sustained mental effort $F(1, 17) = 5.85$, $p = 0.027$, $\eta 2 = 0.26$ (but not a significant multivariate group effect).		
Control:<br>Children received a neuropsychological and diagnostic evaluation to promote the protective factor of early diagnosis and to identify the child's neuropsychological profile (Personalized feedback to caregivers)	–Satisfaction with FoT<br>–Child's emotional and behavioral functioning<br>–Child's impairment<br>–Child's self-perception and environment<br>–Child's behavioral problems<br>–Parental knowledge and advocacy<br>–Families' needs met<br>–Parenting strategies and parental attributions for child misbehavior<br>–Efficacy in parenting role and satisfaction with parenting role:<br>–Perceives support from family, friends, significant	Satisfaction with FoT:<br>–CSQ: high satisfaction<br>–PEI-FOT: high level of enjoyment; felt that they could apply what they learned; good relationship with their FMF Specialist; felt the Specialist understood their feelings and problems; children generally looked forward to coming to the group; and learned new skills; children had relatively more difficulty applying what they learned<br>Child's emotional and behavioral regulation:<br>ERC:<br>–Emotion regulation: significant group difference: parents reported a change in child emotion regulation (ERC $d_{ppc} = 1.18$). This effect reflected a medium to large improvement in emotion regulation for the intervention group and a medium-sized decrement for the comparison group<br>–Negative effect: main effect of time<br>Child's impairment:<br>IRS: medium to large group effect size for parent-reported self-esteem was found (IRS self-esteem $d_{ppc} = 0.77$), which was not statistically different between groups. (significant effect of time $p = 0.046$); main effect of time for global impairment<br>Child's self-perception and environment:<br>BPI: medium to large group effect was found for child-reported anxiety symptoms (BPI Overanxious $d_{ppc} = 0.75$), which did not reach statistical significance. FoT had a higher level of anxiety symptoms at preintervention with improvement over time (d within $= 0.80$) Controls had a minimal change in self-reported anxiety. Main effect of time for children to report prosocial skills and conduct problems<br>Child's behavioral problems:<br>ECBI: Main effect of time for parental report of child disruptive behavior	–Families were not precluded from participating in other intervention programs<br>–Fidelity was monitored in weekly individual/group supervision<br>–Attempts were made to blind the research assistant to intervention conditions at assessment points<br>–Small sample size powered to detect only large effects<br>–Effect sizes are resistant to sample size influence and give a truer measure of the magnitude of effects<br>–3 families declined intervention for logistical reasons and were combined with the control group in analyses<br>–All caregivers	Moderate (RoB-2)

(*Continued*)

 © 2025. Thieme. All rights reserved.

(*Continued*)

Control	Outcomes	Results	Comments	Risk of bias
	others, and involved professionals<br>–Change in self-care<br>–Stress in the parent-child system	Parental knowledge and advocacy:<br>K&A: statistically significant between-group difference: large effect size for knowledge and advocacy with FoT improving (K&A $d_{ppc} = 1.02$)<br>Families' needs met:<br>FNM: significant between-group difference: large effect with FoT improving ($d_{ppc} = 0.72$)<br>No statistical significance:<br>–Parenting strategies and parental attributions for child misbehavior<br>–Efficacy in the parenting role and satisfaction with parenting role<br>–Perceives support from family, friends, significant others, and involved professionals<br>–Change in self-care<br>–Stress in the parent-child system	completed one individualized session; 12 completed 2 individualized sessions; 11 completed the school consultation<br>–Children in both groups declined in their self-esteem. FoT may have buffered this decline<br>–Highly motivated families<br>–Some families had logistical reasons not to participate in the intervention<br>–Lack of objective data (parental reports)<br>–Findings could be due to the more intensive care with FoT compared to controls	
Control:<br>Children received a neuropsychological and diagnostic evaluation to promote the protective factor of early diagnosis and to identify the child's neuropsychological profile (Personalized feedback to caregivers)	6 mo sustainability of:<br>–Child's emotional and behavioral functioning<br>–Child's impairment<br>–Child's self-perception and environment<br>–Child's behavioral problems<br>–Parental knowledge and advocacy<br>–Families' needs met<br>–Parenting strategies and parental attributions for child misbehavior<br>–Efficacy in parenting role and satisfaction with parenting role:<br>–Perceives support from family, friends, significant others, and involved	Child's emotional and behavioral regulation:<br>ERC:<br>–Emotion regulation: FoT had medium-large improvements during the intervention and declined in follow-up (remained above baseline levels; d within $= -0.56$); controls had a moderate worsening during the study and improved in follow-up (slightly below baseline levels; d within $= 0.38$). Significant group*time effect: changes in emotion regulation over time differed significantly by treatment group ($F(2, 44) = 8.032$, $p = 0.001$)<br>–Negative affect: FoT had a small-medium improvement during the intervention and an additional minimal-small improvement in follow-up (d within $= 0.15$); controls had a minimal improvement during the study and a small improvement in follow-up (d within $= 0.24$), both groups had a similar magnitude of change. Significant time effect: significantly higher levels of negative affect at baseline than at 6-mo follow-up ($F(2, 44) = 4.68$, $p = 0.014$), no significant group or group*time effect<br>Child's impairment:<br>IRS: FoT remained stable during the intervention and had a moderate decrease in follow-up (d within $= -0.41$); controls had a medium-large decline during the study and a minimal decline in follow-up (d within $= -0.10$). Significant time effect: $F(2, 38) = 10.07$, $p = 0.018$), no significant group or group*time effect. Both groups had a decline in self-esteem<br>Child's behavioral problems:<br>ECBI: FoT group moderately decreased in behavioral intensity during the course of the intervention and maintained this change over the 6-mo follow-up interval (d within $= 0$). Children in the comparison group, who had a small decrease in behavioral intensity during the intervention time, had an additional small decrease in intensity of behavior problems during the last 6 mo (d within $= -0.25$). When considering an overall change from baseline to follow-up, effect size analysis showed negligible group difference ($d_{ppc} = 0.03$).<br>Significant time effect, with the most intense behaviors at baseline,	–Families were not precluded from participating in other intervention programs<br>–Fidelity was monitored in weekly individual/group supervision<br>–Attempts were made to blind the research assistant to intervention conditions at assessment points<br>–Small sample size powered to detect only large effects<br>–Effect sizes are resistant to sample size influence and give a truer measure of the magnitude of effects<br>–3 families declined intervention for logistical reasons and were combined with the control group in analyses<br>–All caregivers completed one individualized session; 12	Moderate (RoB-2)

 © 2025. Thieme. All rights reserved.

(*Continued*)

Control	Outcomes	Results	Comments	Risk of bias
	professionals<br>–Change in self-care<br>–Stress in the parent-child system	significantly less intense behaviors at postintervention, and significantly less intense behaviors at 6-mo follow-up (F(2, 44) = 16.77, $p < 0.001$) on average across groups. No significant main effect for the group and no significant group*time interaction<br>Parental knowledge and advocacy:<br>K&A: FoT had large gains during the intervention and maintained them in follow-up; controls had approximately the same level across all time points. Significant main effect of time; no group effect; significant group*time effect (F(2, 40) = 3.241, $p < 0.050$), with families in the intervention group reporting significantly less knowledge at baseline (M = 26.50) compared to postintervention (M = 31.00) and 6-mo follow-up (M = 31.21))<br>Families' needs met:<br>FNM: FoT had a large increase in needs met during the intervention and had a large decline (d within = −1.03) in the follow-up (remained above baseline-level and reflected an overall medium-level improvement (d within = 0.55)); controls had a small-medium increase during the study and a large decline (d within = −1.03) in follow-up (below baseline-level (d within = −0.60)). The overall group effect across the length of the study was large ($d_{ppc} = 1.07$), and favored the intervention group (M = 3.2) as compared to the comparison group (M = 2.8; F(1, 20) = 4.682, $p = 0.043$). Main effect for time: scores for FNM were significantly higher for both groups immediately postintervention (M = 3.37) than they were at baseline (M = 2.84) or follow-up (M = 2.79; F(2, 40) = 6.78, $p = 0.003$). Significant group*time interaction (F(2, 40) = 2.90, $p = 0.067$) with FoT reporting that their needs were better-met postintervention (M = 3.62) compared to baseline (M = 2.81), and the comparison group reporting their needs were better-met postintervention (M = 3.13) compared follow-up (M = 2.41)<br>Efficacy in the parenting role and satisfaction with parenting role<br>–PSOC: Parenting self-efficacy: FoT had a small-medium improvement during the study and an additional small-medium improvement (d within = 0.34) in follow-up; controls had a minimal change during the study and a small-medium worsening (d within = −0.39) in follow-up. Large group effect in follow-up ($d_{ppc} = 1.14$ large effect size), favoring FoT. Changes in efficacy over time significantly differed by treatment group (F(2, 44) = 3.51, $p = 0.038$)<br>–Parenting satisfaction: FoT had a medium improvement during the intervention and a small-medium worsening (d within = −0.37) in follow-up (remaining above baseline); controls had a minimal change during the study and a moderate improvement (d within = 0.52) in follow-up (similar level to FoT postintervention). Significant group*time interaction, with parenting satisfaction in the comparison group significantly higher at follow-up (M = 38.3) than at baseline (M = 34.6) or postintervention (M = 34.3; F(2, 44) = 3.48, $p = 0.039$). Small-moderate group difference favoring the comparison group ($d_{ppc} = -0.38$)<br>Stress in the parent-child system<br>PSI: both groups had minimal-small changes in distress across each time point; FoT had minimal-small improvements (d within = −0.11) in follow-up; controls had minimal-small worsening (d within = 0.13) in follow-up. Small group effect at follow-up compared to baseline ($d_{ppc} = 0.21$), no significant time effect, no significant group or group*time effect<br>Outcomes meeting statistical significance (treatment x group: $p < 0.05$) or practical significance ($d_{ppc} = 0.41$):<br>–Parents' outcomes: self-efficacy ($p = 0.039$; dpcc = 1.14), family needs met ($p = 0.067$; dpcc = 0.67), FASD knowledge ($p = 0.050$; dpcc = 0.60), parenting satisfaction ($p = 0.038$; dpcc = −0.38)	completed 2 individualized sessions; 11 completed the school consultation<br>–Children in both groups declined in their self-esteem. FoT may have buffered this decline<br>–Highly motivated families<br>–Some families had logistical reasons not to participate in an intervention<br>Follow-up:<br>–Lack of objective data (parental reports)<br>–3 participants completed the follow-up measures at home<br>–The decline in families' needs met could be due to an increase in needs as children progress in early school years<br>–Data collection at different time points: baseline (summer), posttest (summer), follow-up (winter)<br>–No intent-to-treat analysis results could overestimate the treatment effect	

(*Continued*)

 © 2025. Thieme. All rights reserved.

(*Continued*)

Control	Outcomes	Results	Comments	Risk of bias
		–Children's outcomes: emotion regulation ($p = 0.001$; dpcc = 0.30), self-esteem ($p = 0.294$; dpcc = 0.56).		
Control:<br>Adolescents and caregivers got written materials on alcohol misuse and stress reduction.	–Prevention and reduction of alcohol-related negative outcomes<br>–Determination of possible increase in alcohol risk in abstinent youths<br>–Satisfaction of SUI	Prevention and reduction of alcohol-related negative outcomes:<br>–Light/moderate drinkers (postintervention): significant treatment effects, with SUI having significantly lower levels of alcohol risk and fewer negative behaviors than controls: AUDIT ($F(1, 15) = 5.43$, $p = 0.03$, $d = 1.08$) and RAPI ($F(1, 15) = 8.60$, $p = 0.01$, $d = 0.99$). No significant differences in CRAFFT<br>–Light/moderate drinkers (Follow-up): Gains in RAPI sustained ($F(1, 15) = 4.53$, $p = 0.05$, $d = 0.83$). Gains in AUDIT reached a nonsignificant large effect size ($d = 0.76$)<br>Determination of possible increase in alcohol risk in abstinent youths:<br>No group differences at baseline; no differences or change in outcome variables at posttest or at 3 mo follow-up<br>Satisfaction of SUI:<br>–Adolescents: 96% reported being confident in avoiding risky situations based upon what they have learned; 92% reported the program to be helpful<br>–Caregivers: 96% stated that they believe the program helps the teens to make better choices regarding alcohol; 96% reported to be satisfied	–Trained and qualified group leaders, standardized manuals, ongoing weekly supervision, and live monitoring of sessions.; fidelity rating ≥ 95%<br>–No detailed definition of drinker-type; no heavy drinker-type classification<br>–No significant differences in CRAFFT might be due to the low occurrence of behaviors measured in CRAFFT<br>–Motivated caregivers who actively seek help<br>–Impact of caregivers has not been assessed<br>–Impact of age has not been analyzed (large age range)	Moderate (RoB-2)
Control:<br>No intervention	–Effectiveness of STABEL on balance and motor performance<br>–Feasibility in laboratory and home setting	Motor skills<br>MABC-2:<br>–Balance standard score: no significant interaction, but significant differences by session ($p = 0.02$) and group ($p = 0.04$); home group had significant improvements compared to controls ($p = 0.01$); home and lab group (together) had significant improvements from pretest to 1 wk ($p = 0.004$), but no significant improvements from pretest ($p = 0.09$) or 1 wk ($p = 0.11$) to 1 mo<br>–Total Motor standard score: significant interaction ($p = 0.05$); significant differences by session; home and lab group (together) had significant improvements from pretest to 1 wk, and pretest to 1 mo, but not from 1 wk to 1 mo<br>Dynamic balance:<br>DGI: no significant differences across time or between groups<br>Static balance:<br>P-CTSIB-2: Total Sensory Score: significant interactions ($p = 0.02$) and significant improvements for home STABEL compared to controls ($p = 0.01$); trends show higher postintervention scores for lab and home groups	–P-CTSIB-2 is only suitable for children aged 6–12 y<br>–Small dose of STABEL<br>–No randomization<br>–No control for fidelity in-home group and for other parallel interventions<br>–Possible ceiling effect might explain no detected changes in dynamic balance<br>–Pre–and 1-wk-test differences were beyond the error of the MABC-2 test, at a level that also suggests potential clinical significance<br>–Lab group did not show significant improvements in any test compared to the controls<br>–Home group had	High (ROBINS-I)

 © 2025. Thieme. All rights reserved.

(*Continued*)

Control	Outcomes	Results	Comments	Risk of bias
			overall milder CNS dysfunction based on their FASD diagnosis; the lab group had more muscular weakness and<br>–1/3 who agreed to the home intervention did not complete the training protocol due to unknown reasons, frustration, or dizziness	
NA	–Feasibility of STABEL<br>–Immediate effect on sensory attention and postural control	Feasibility: all participants interacted with STABEL and completed all training blocks<br>For FASD children:<br>1. block:<br>–fun: 82% had fun, 18% felt ok, 0% had no fun<br>–dizziness: 0% felt dizzy, 18% felt a little dizzy, 82% had no dizziness<br>2. block:<br>–fun: 100% had fun<br>–dizziness: 9% felt dizzy, 9% felt a little dizzy, 82% had no dizziness<br>3. block:<br>–fun: 55% had fun, 18% felt ok, 27% had no fun<br>–dizziness: 18% felt dizzy, 0% felt a little dizzy, 82% had no dizziness<br>For all children:<br>Postural control:<br>–No significant interactions for ellipse area of body sway or velocity outcomes<br>–Significantly higher medial-lateral and anterior-posterior RMS velocities in post STABEL in most conditions in both groups ($p \leq 0.01$ to 0.05)<br>–No significant differences in ellipse area of body sway pre compared to posttesting or FASD compared to TD<br>Sensory attention:<br>–Entrainment gain: LLM = visual screen gain and tilt board gain increased significantly from pre–to posttesting only in TD ($p = 0.08$); LLL ($p = 0.06$) and LLH ($p = 0.09$) = significantly higher touch pole entrainment gain in both groups in posttesting; LLH = significantly higher visual screen gain in both groups in posttesting ($p = 0.02$); HHL = significantly lower touch pole gain in posttesting ($p = 0.02$)<br>–SAF: No significant interaction or pre/post differences	–Dizziness did not persist<br>–Different exclusion criteria for FASD and TD children (TD children might have other diagnoses)<br>–Examiner was not blinded for FASD or TD<br>–$\alpha = 0,1$<br>–Decreased postural stability could be due to fatigue (long testing sessions of 2.5h)<br>–One-time practice with STABEL might be not enough to change sensory attention fractions<br>–Body sway without any extra sensory stimulation has not been measured<br>–Measures of balance and functional motor performance have not been included to complement kinematic measures of sensory attention and postural control	Low (ROBINS-I modified)
NA	–Children's developmental skills<br>–Children's functional capacity<br>–Parental skills<br>–Parental stress	Baseline: all children had clinically significant deficits in all 4 functional domains in the CMR; all parents had clinically significant parenting stress<br>Children's developmental skills:<br>BBDI-2 Total Score: Statistically significant improvements from preintervention to postintervention (premean and 95% CI: 0.205 [0.148, 0.261]; Postmean and 95% CI: 0.518 [0.394, 0.641]; Standard error: 9.80; Standardized test statistic: 2.81; r (rank-biserial correlation): 0.63; p: 0.005*)<br>Children's functional capacity:<br>–NMT Total Score: Statistically significant improvements from preintervention	–Therapists were under the supervision of a licensed pediatric psychologist who was trained in CPP, Mindful Parenting, and NMT<br>–Significant amount of families did not complete intervention	NI (ROBINS-I modified)

(*Continued*)

 © 2025. Thieme. All rights reserved.

(*Continued*)

Control	Outcomes	Results	Comments	Risk of bias
		to postintervention (premium and 95% CI: 23.40 [18.56, 28.24); Postmean and 95% CI: 45.20 [40.88, 49.52]; Standard error: 9.79; Standardized test statistic: 2.81; r (rank-biserial correlation): 0.64; p: 0.005*)<br>–NMT Cortical Modulation Ratio: Statistically significant improvements from preintervention to postintervention (premean and 95% CI: 0.205 [0.148, 0.261]; Postmean and 95% CI: 0.518 [0.394, 0.641]; Standard error: 9.80; Standardized test statistic: 2.81; r (rank-biserial correlation): 0.63; p 0.005*)<br>Parental skills:<br>Satisfaction survey: 18 reported an improvement in different areas of parental skills<br>Parental stress:<br>PSI-SF Total Score: Statistically significant improvements from preintervention to postintervention (premean and 95% CI: 23.40 [18.56, 28.24; Postmean and 95% CI:] 45.20 [40.88, 49.52]; Standard error: 9.79; Standardized test statistic −2.81; r (rank-biserial correlation): −0.63; p: 0.005*)	(unknown reasons)<br>–Improvements observed by multiple reporters: clinicians, parents<br>–Possible effect of time<br>–Different somatosensory interventions for each child<br>–Unknown impact of CPP, MPE, and NMT alone<br>–Very short evaluation window (unknown long-term effects)	
MILE:<br>–Individualized program that is based on specific math deficits and learning needs<br>–One-on-one-instruction<br>–30 min. sessions, 1–2 times a week over 5–7 wks (total of 5 h)	–Social skills and competing problem behaviors<br>–Social, emotional, and behavioral problem areas	Social skills and competing problem behaviors<br>SSIS-RS:<br>–No significant impact of SSIS-IG on the SSIS-RS composite scores social skills F (1, 26) = 0.016, $p = 0.90$; problem behaviors F(1, 26) = 2.81, $p = 0.12$ relative MILE<br>–Analysing differences between participants pre–and posttest SSIS-RS scores separately within each intervention (paired-sample *t*-tests): SSIS-IG improved significantly on problem behavior scale (decrease by 8.6 standard points; t (13)= 2.52, $p = 0.03$) compared to MILE (decrease by 1.7; t(13) = 0.76, $p = 0.46$)<br>Social, emotional, and behavioral problem areas<br>CBCL: Social composite approached significance F(1, 21) = 3.4, $p = 0.08$; however it did not approach significance for the social problems subtest F(1, 21)= 0.54, $p = 0.47$.	–Inclusion of children with ADHD or ODD<br>–Groups not randomized but matched by age, diagnosis, IQ, and gender<br>–Group assignment after pretests<br>–Families with two children in the study were allowed to have both children in the same group (sibling pairs $n = 2$)<br>–Posttests by a blinded research assistant<br>–SSIS-IG: normally in a group setting<br>–Caregivers who are able to have their child participate in a study may also be more likely to connect their children with various social activities<br>–Possible impact of individualized attention from researchers on problem behavior (both groups)<br>–Unknown long-term effects<br>–Possible impact of MILE on social skills<br>–Possible mismatch between the degree of each type of deficit	Low (ROBINS-I)

 © 2025. Thieme. All rights reserved.

(*Continued*)

Control	Outcomes	Results	Comments	Risk of bias
			targeted within SSIS and children's social skills impairments<br>–Baseline: all children in the clinical range and significantly different than the normative mean on social skills, the Externalizing Problem scale and Total Problems scale<br>–Improvements of SSIS-IG on problem behavior cannot be exclusively attributed to the intervention<br>–CBCL social scales were only available for participants ≥ 5 (*n* for each group = 12).	
SOC:<br>–Children training in group sessions that were process-oriented and behaviorally based, involving group discussion and co-operative projects. Training involved discussion and practice of rules of social behavior typically thought important by adults, but not necessarily empirically demonstrated to be predictive of peer acceptance nor often practiced by socially skilled children in naturalistic settings. 12 90-min sessions over the course of 12 wks<br>–No parent training	–Knowledge of social skills<br>–Child self-concept<br>–Overall social skills<br>–Behavior problems (parent-report)<br>–Comparison between children with and without FASD	Knowledge of social skills:<br>TSSK: Significant condition effect, with CFT showing significantly improved knowledge of appropriate social skills compared to SOC, F(1, 62) = 21.34, $p < 0.0001$, d = 1.22 (95% CI (0.69, 1.73)); $F^2 = 0.34$ (95% CI (0.09, 0.90)). No other significant main or interaction effects<br>Child self-concept:<br>Piers Harris 2: Significant condition effect, with CFT showing significantly improved overall self-concept, F(1, 62) = 4.21, $p < 0.05$, especially on individual domains of self-concept, children reported improved behavioral adjustment F(1, 62) = 5.69, $p < 0.02$, d = 0.58 (95% CI (0.09, 1.07)), $F^2 = 0.09$ (95% CI (0.004, 0.36)); intellectualschool status, F(1, 62) = 6.01, $p < 0.02$, d = 0.39 (95% CI (-0.10, 0.87)), $F^2 = 0.10$ (95% CI (0.006, 0.34)); and freedom from anxiety, F(1, 62) = 7.63, $p < 0.01$,d = 0.70 (95% CI (0.21, 1.19)), $F^2 = 0.12$ (95% CI (0.008, 0.42)), compared to SOC. No significant improvement in physical appearance, F(1, 62) = 0.12, $p = 0.73$, popularity, F(1, 62) = 0.51, $p = 0.48$, or happiness and satisfaction, F(1, 62) = 1.85, $p = 0.18$. No other significant main or interaction effects<br>Overall social skills:<br>SSRS-P: No significant condition effect in the improvement of overall social skills, F(1, 62) = 2.37, $p = 0.12$ because the 2 groups differed on their pretreatment social skills scores. Some children in the SOC group started out scoring higher than the children in the CFT group and actually demonstrated a significant decline in social skills according to parent reports. The CFT group, while showing a significant 18-point improvement compared to the improvement of 4 points in the SOC group, did not differ from the SOC group after controlling for pretreatment levels. Analyses of individual index scores revealed statistically significant condition effects for assertion, F(1, 62) = 4.04, $p < 0.05$, d = 0.18 (95% CI (−0.31, 0.66)), $F^2 = 0.07$ (95% CI (0.0009, 0.28)); and responsibility, F(1, 62) = 4.53, $p < 0.04$, d = 0.16 (95% CI (-0.32, 0.64)), $F^2 = 0.07$ (95% CI (0.001, 0.31)); in favor of the CFT condition over the SOC condition. Analyses of cooperation, F(1, 62) = 0.30, $p = 0.59$, and self-control, F(1, 62) = 0.75, $p = 0.39$, did not yield statistically significant effects. No other significant main or interaction effects<br>Parent satisfaction questionnaire:	–Possible impact of involvement of parents in the program on subjective outcome measures<br>–Children reported changes themselves<br>–No independent evaluation of children's behavior in a naturalistic setting<br>–No child with FAS<br>–Only families included parents who actively sought help for their children and who were highly motivated to participate<br>–2 children were asked to leave the program because of significant disruptive behavior (CFT and SOC)<br>–Therapists in the SOC condition were provided weekly supervision by their supervisors	Moderate (ROB-INS-I)

(*Continued*)

 © 2025. Thieme. All rights reserved.

(*Continued*)

Control	Outcomes	Results	Comments	Risk of bias
		–90.7% in CFT and 68.6% in SOC reported confidence in their children's ability to get along better with other children because of treatment ($p < 0.04$)<br>–87.5% in the CFT and 57% in SOC reported that they were confident that they were better able to help their children make and keep friends because of the treatment ($p < 0.007$)<br>–Groups were comparable in their overall satisfaction with ($p < 0.68$). Overall, 93.7% of CFT and 88.6% of SOC reported being very satisfied or highly satisfied with the treatment<br>Therapist satisfaction questionnaire:<br>In CFT, 84% agreed that the treatment was helpful, 100% agreed that their clients enjoyed the treatment, and 92% agreed that they would like to see the program adopted permanently at the Child and Family Guidance Centre and would continue to use it. Concerns: the program was hard to integrate into busy schedules, and more time needed<br>Treatment is equally effective for children with and without PAE		
NA	–Individual needs<br>–Goal attainment<br>–Caregiver stress<br>–Satisfaction	Individual needs and goal attainment:<br>–Length of time in the program was significantly related to both needs ($r = -0.27$, $p < 0.001$) and goals ($r = 0.22$, $p < 0.001$) indicating that the longer families spent in the program, the greater their reduction in needs and achievement of goals<br>–Individual needs significantly decreased from pre to postprogram: $F(1, 187) = 152.69$, $p < 0.001$, $\eta p2 = 0.45$<br>–Significant increase in goal achievement from pre–to postprogram: $F(1, 165) = 317.46$, $p < 0.001$, $\eta p2 = 0.66$<br>Caregiver stress:<br>Significant decrease in overall levels of caregiver stress from pre–to postprogram: $F(1, 72) = 39.409$, $p < 0.001$, $\eta p2 = 0.354$<br>No gender or age effect<br>Satisfaction:<br>–High satisfaction with the program (98%) and willingness to participate again (99%); 32.1% of caregivers reported parenting and handling their child better, 28.2% reported understanding their child and/or FASD better, and 14.5% reported feeling less stressed, having increased patience, and being more positive. 65.6% reported that they had not experienced any problems with the program<br>–Reported challenges: feeling that mentor did not understand what it was like to live with a child with FASD, difficulties collaborating with other services, and a need for longer-term support. 38.8% reported that there was no need for improvement or were unsure.	–Self-referred recruitment<br>–No control for quality of mentorship, participation in other services, family variables, or comorbid disorders<br>–Many files were not included in the analyses as there were no post needs or goals measures available (possible bias)	Moderate (ROB-INS-I modified)
NA	Influence of extrinsic motivation on response time (RT) and accuracy as measures of interference control (ability to suppress competing distracters to carry out a target response)	Significant between-group differences on FSIQ (Wechsler Intelligence Scale for Children-4th Edition): AE < ADHD < CON<br>Inhibitory control performance:<br>–Accuracy: AE was significantly slower than ADHD ($p = 0.038$) and CON ($p < 0.001$) and significantly slower in incongruent trials compared to congruent trials ($p < 0.001$)<br>–RT: AE had significantly poorer accuracy in incongruent trials than CON ($p = 0.001$) and significantly poorer accuracy in incongruent trials compared to congruent trials ($p < 0.001$). Age significantly interacted with the condition [$F(1.235, 103.725) = 7.74$, $p = 0.004$, $\eta p2 = 0.084$] and flanker type [$F(2.144, 180.130) = 4.24$, $p = 0.014$, $\eta p2 = 0.048$]<br>Response to reward:<br>–In all conditions, AE was significantly slower than CON ($p < 0.001$) and slower than ADHD ($p = 0.046$; except for REW, $p = 0.051$)	–Study was part of a larger project at the Centre for Behavioral Teratology at San Diego State University. Flanker task was the third out of four computerized attention tasks that lasted about 1 h and 45 min in total and required a long duration of attention (possible impact on outcome)	Moderate (ROB-INS-I)

 © 2025. Thieme. All rights reserved.

(*Continued*)

Control	Outcomes	Results	Comments	Risk of bias
		–For all groups, RT in the NR condition was significantly slower than in the other conditions ($p = 0.002$)<br>–For AE ($p > 0.39$) and ADHD ($p > 0.19$), RT was similar for all 3 reinforcement conditions<br>–For CON, ROR improved RT compared to REW ($p = 0.03$)<br>–All groups improved with reinforcement in RT, but CON showed the most improvement in RT when response cost was applied. For AE and ADHD, the type of reinforcement was not critical<br>–For RT, the main effect of the group [F (1, 84) = 10.69, $p < 0.001$, ηp2 = 0.203] with AE being slower than CON and ADHD $p < 0.017$)<br>–For RT, the main effect of flanker type [F (2.144, 180.130) = 11.70, $p < 0.001$, ηp2 = 0.122]. RTs were significantly slower without reinforcement ($p < 0.001$)<br>–For congruent trials, accuracy was better in the NR condition compared to REW ($p = 0.03$) and ROR ($p = 0.01$)<br>–For incongruent trials ($p < 0.001$) and neutral trials ($p < 0.029$), accuracy was better in the NR condition compared to all other conditions<br>–Accuracy was poorer for incongruent trials compared to all other trials ($p < 0.001$)<br>–For ROR, accuracy was significantly poorer for neutral trials compared to congruent trials ($p = 0.019$)<br>–For EQ, accuracy was poorer for neutral trials compared to congruent ($p = 0.005$) or single trials ($p = 0.03$)<br>–For accuracy, the main effect of flaker type [F (1.530, 130.049) = 118.06, $p < 0.001$, ηp2 =.581] and condition [F (2.309, 196.230) = 37.01, $p < 0.001$, ηp2 = 0.303]<br>–For accuracy, no main effect of the group	–Children were asked to abstain from medication use on the day of testing. However, 7 AE and 2 ADHD took medication<br>–AE showed greater difficulties with executive control<br>–Regarding RT, AE and ADHD benefited similarly from both types of extrinsic reinforcement<br>–Regarding accuracy, all groups showed better performance without reinforcement for all conditions except for single targets<br>–Study utilized primary and secondary reinforcement<br>–Oppositional defiant disorder and conduct disorder have not been assessed<br>–No child with ADHD symptoms had the hyperactive/impulsive type; all of them had the inattentive or combined type<br>–Analyses were repeated without the 5 AE without ADHD same results<br>–IQ was significantly correlated with accuracy in AE and CON, and with RT in ADHD	
Standard Information group:<br>–Paper form<br>–Information packets regarding the diagnosis, neurodevelopmental consequences, and access to community services and	–Satisfaction<br>–Knowledge about FASD<br>–Behavioral changes in children	Satisfaction (Likert scale response and open-ended questions):<br>–All groups: high satisfaction<br>–Workshop group: higher ratings on usefulness, understandability, amount, overall satisfaction, and willingness to recommend than Standard Information group<br>–Workshop group: higher ratings on the amount of information and overall satisfaction than Internet group<br>Knowledge (Caregiver advocacy knowledge questionnaire (CA) and Behavioral regulation knowledge questionnaire (BR)):<br>–Standard Information group: significant gains in knowledge on behavioral regulation (BR: t (17) = −2.7, $p < 0.01$, $\eta p^2 = 0.305$); the only trend for improvement on the caregiver advocacy knowledge (CA: t (17) = −1.9,	–Gender differences between the groups as a potential reason for the Internet group not showing signs. Improvements<br>–Significantly more participants with a higher dysmorphia score dropped out of the Internet group. This trend was also	Moderate (ROB-2)

(*Continued*)

 © 2025. Thieme. All rights reserved.

(*Continued*)

Control	Outcomes	Results	Comments	Risk of bias
information sources		$p < 0.08$, $\eta p^2 = 0.170$)<br>–Workshop group: significant gains in both areas of knowledge: Caregiver advocacy (CA: t (21) = −3.9, $p < 0.001$, $\eta p^2 = 0.422$; BR: (t (11)= −6.7, $p < 0.0001$, $\eta p^2 = 0.668$)<br>–Internet group: significant gains in both areas of knowledge (CA: t (11) = −2.8, $p < 0.02$, $\eta p^2 = 0.412$; BR: (t (11) = −3,4, $p < 0.005$, $\eta p^2 = 0.526$)<br>–Significant time effect and group effect<br>–No significant group*time effect, but a trend was found in the BR data (F (2, 50) = 2.0, $p < 0.152$, $\eta p^2 = 0.073$) for the Internet group gaining more knowledge than the Standard Information group<br>–Strongest relationship between caregiver educational level and knowledge gains was in the Standard Information group (CA: $r = -0.36$, $p < 0.16$ and BR: $r = 0.44$, $p < 0.08$) as compared to the Workshop (CA: $r = -0.15$, $p < 0.52$ and BR: $r = 0.25$, $p < 0.26$) and Internet (CA: $r = -0.03$, $p < 0.93$ and BR: $r = 0.18$, $p < 0.57$) groups<br>Child behavioral changes (Children behavior checklist):<br>–Standard Information group: trend in improving total problem behavior (t (1, 17) = 1.8, $p < 0.09$, $\eta p^2 = 0.164$) and externalizing problem behavior (t (1, 17) = 2.0, $p < 0.06$, $\eta p^2 = 0.192$)<br>–Workshop group: For total problem behaviors, a significant effect was found (t (1, 21) = 2.7, $p < 0.014$, $\eta p^2 = 0.254$)<br>–Internet group: no changes in total problem behavior<br>–On the total problems scale, a significant treatment by group effect was found (F (2, 50) = 3.2, $p < 0.048$, $\eta p^2 = 0.115$) with improvements in behavioral ratings only in the Standard Information and Workshop groups<br>–On the externalizing scale, a significant general treatment effect by gender (F (1, 50) = 5.3, $p < 0.026$, $\eta p^2 = 0.096$) was found with males showing greater improvements than females and a trend was found for a time by group effect (F (2, 50) = 2.9, $p < 0.064$, $\eta p^2 = 0.104$) with those in the Standard Information and Workshop groups showing improved behavior but those in the Internet group not. For the internalizing scale, there was a trend for a general treatment effect (F (1, 50) = 2.2, $p < 0.14$, $\eta p^2 = 0.043$) with posttest scores being lower than those at the pretest<br>–Examination of the pattern of change scores found on the externalizing and total problem scores suggested that the treatment effects were skewed (Externalizing = 1.175 and Total = 1.492) such that about 50% of participants made positive gains with half of these making what could be termed clinically significant changes (> 1/2 of standard deviation).	seen in the Standard Information group, but not in the Workshop group<br>–Initiation of the Internet group was the biggest hurdle (once logged in the method was effective)<br>–Caregiver educational level had the strongest relationship with knowledge gains in the Standard Information group (not significant)<br>–Only 50% of the children showed improvements, only 25% showed significant improvements only effective for some families<br>–Content differences between the Standard Information group and Workshop group/ internet group	

Note: RCT, randomized controlled trials; ROBINS-I, a tool for assessing the risk of bias in nonrandomized studies of interventions; ROB-2, Cochrane risk-of-bias tool (2nd version).

 © 2025. Thieme. All rights reserved.

References for Supplementary Document 2

1 Śmiarowska M, Brzuchalski B, Grzywacz E, et al. Influence of *COMT* (rs4680) and *DRD2* (rs1076560, rs1800497) gene polymorphisms on safety and efficacy of methylphenidate treatment in children with fetal alcohol spectrum disorders. Int J Environ Res Public Health 2022;19(08):4479

2 Nguyen TT, Risbud RD, Mattson SN, Chambers CD, Thomas JD. Randomized, double-blind, placebo-controlled clinical trial of choline supplementation in school-aged children with fetal alcohol spectrum disorders. Am J Clin Nutr 2016;104(06):1683–1692

3 Wozniak JR, Fuglestad AJ, Eckerle JK, et al. Choline supplementation in children with fetal alcohol spectrum disorders has high feasibility and tolerability. Nutr Res 2013;33(11):897–904

4 Wozniak JR, Fuglestad AJ, Eckerle JK, et al. Choline supplementation in children with fetal alcohol spectrum disorders: a randomized, double-blind, placebo-controlled trial. Am J Clin Nutr 2015; 102(05):1113–1125

5 Wozniak JR, Fink BA, Fuglestad AJ, et al. Four-year follow-up of a randomized controlled trial of choline for neurodevelopment in fetal alcohol spectrum disorder. J Neurodev Disord 2020;12(01):9

6 Smith SM, Virdee MS, Eckerle JK, et al. Polymorphisms in SLC44A1 are associated with cognitive improvement in children diagnosed with fetal alcohol spectrum disorder: an exploratory study of oral choline supplementation. Am J Clin Nutr 2021;114(02):617–627

7 Boroda E, Krueger AM, Bansal P, et al. A randomized controlled trial of transcranial direct-current stimulation and cognitive training in children with fetal alcohol spectrum disorder. Brain Stimul 2020;13(04):1059–1068

8 Vidal R, Vidal L, Ristol F, et al. Dog-assisted therapy for children and adolescents with fetal alcohol spectrum disorders a randomized controlled pilot study. Front Psychol 2020;11:1080

9 Kerns KA, Macoun S, MacSween J, Pei J, Hutchison M. Attention and working memory training: a feasibility study in children with neurodevelopmental disorders. Appl Neuropsychol Child 2017;6 (02):120–137

10 Kable JA, Taddeo E, Strickland D, Coles CD. Community translation of the math interactive learning experience program for children with FASD. Res Dev Disabil 2015;39:1–11

11 Kully-Martens K, Pei J, Kable J, Coles CD, Andrew G, Rasmussen C. Mathematics intervention for children with fetal alcohol spectrum disorder: a replication and extension of the math interactive learning experience (MILE) program. Res Dev Disabil 2018; 78:55–65

12 Wells AM, Chasnoff IJ, Schmidt CA, Telford E, Schwartz LD. Neurocognitive habilitation therapy for children with fetal alcohol spectrum disorders: an adaptation of the alert program. Am J Occup Ther 2012;66(01):24–34

13 Soh DW, Skocic J, Nash K, Stevens S, Turner GR, Rovet J. Self-regulation therapy increases frontal gray matter in children with fetal alcohol spectrum disorder: evaluation by voxel-based morphometry. Front Hum Neurosci 2015;9:108

14 Nash K, Stevens S, Greenbaum R, Weiner J, Koren G, Rovet J. Improving executive functioning in children with fetal alcohol spectrum disorders. Child Neuropsychol 2015;21(02):191–209

15 Coles CD, Kable JA, Taddeo E, Strickland DC. A metacognitive strategy for reducing disruptive behavior in children with fetal alcohol spectrum disorders: GoFAR pilot. Alcohol Clin Exp Res 2015;39(11):2224–2233

16 Coles CD, Kable JA, Taddeo E, Strickland D. GoFAR: improving attention, behavior and adaptive functioning in children with fetal alcohol spectrum disorders: brief report. Dev Neurorehabil 2018;21(05):345–349

17 Kable JA, Taddeo E, Strickland D, Coles CD. Improving FASD children's self-regulation: piloting phase 1 of the GoFAR intervention. Child Fam Behav Ther 2016;38(02):124–141

18 Petrenko CLM, Pandolfino ME, Robinson LK. Findings from the families on track intervention pilot trial for children with fetal alcohol spectrum disorders and their families. Alcohol Clin Exp Res 2017;41(07):1340–1351

19 Petrenko CLM, Demeusy EM, Alto ME. Six-month follow-up of the families on track intervention pilot trial for children with fetal alcohol spectrum disorders and their families. Alcohol Clin Exp Res 2019;43(10):2242–2254

20 O'Connor MJ, Quattlebaum J, Castañeda M, Dipple KM. Alcohol intervention for adolescents with fetal alcohol spectrum disorders: project step up, a treatment development study. Alcohol Clin Exp Res 2016;40(08):1744–1751

21 Jirikowic T, Westcott McCoy S, Price R, Ciol MA, Hsu LY, Kartin D. Virtual sensorimotor training for balance: pilot study results for children with fetal alcohol spectrum disorders. Pediatr Phys Ther 2016;28(04):460–468

22 McCoy SW, Jirikowic T, Price R, et al. Virtual sensorimotor balance training for children with fetal alcohol spectrum disorders: feasibility study. Phys Ther 2015;95(11):1569–1581

23 Zarnegar Z, Hambrick EP, Perry BD, Azen SP, Peterson C. Clinical improvements in adopted children with fetal alcohol spectrum disorders through neurodevelopmentally informed clinical intervention: a pilot study. Clin Child Psychol Psychiatry 2016;21(04): 551–567

24 Regehr E. The Impact of an Intervention on Social Skills of Young Children with Prenatal Alcohol Exposure [Master's Thesis, University of Alberta]. Alberta. Accessed 2015 at: https://dx.doi.org/10.7939/r3b56dc77

25 O'Connor MJ, Laugeson EA, Mogil C, et al. Translation of an evidence-based social skills intervention for children with prenatal alcohol exposure in a community mental health setting. Alcohol Clin Exp Res 2012;36(01):141–152

26 Leenaars LS, Denys K, Henneveld D, Rasmussen C. The impact of fetal alcohol spectrum disorders on families: evaluation of a family intervention program. Community Ment Health J 2012; 48(04):431–435

27 Graham DM, Glass L, Mattson SN. The influence of extrinsic reinforcement on children with heavy prenatal alcohol exposure. Alcohol Clin Exp Res 2016;40(02):348–358

28 Kable JA, Coles CD, Strickland D, Taddeo E. Comparing the effectiveness of on-line versus in-person caregiver education and training for behavioral regulation in families of children with FASD. Int J Ment Health Addict 2012;10(06):791–803

 © 2025. Thieme. All rights reserved.

Risk of Bias Assessment—Systematic Reviews

Reference	Last search	Country	Included articles (*n*)	Types of included articles	Programs or intervention types	Population (age in y)	Population (characteristics)	Outcomes reported
Flannigan et al [1]	01.04.2020	Canada	33	Inclusion: original and peer-reviewed, contributed empirical data (quantitative, qualitative, or mixed), and included the following:<br>(i) interventions for individuals of any age with PAE or FASD,<br>(ii) with quantitatively or qualitatively reported outcomes related to mental health and/or substance use, and<br>(iii) published in English, from the year 2000 onward<br>Exclusion: animal studies; dietary or pharmacological interventions	Any intervention to improve mental health (emotional, psychological, spiritual, behavioral, and social well-being) and substance use outcomes	All age groups	Any individuals with PAE and FASD	Mental health (emotional, psychological, spiritual, behavioral, and social well-being) and substance use outcomes
Mela et al [2]	04.02.2017	Canada	25	Only peer-reviewed journal articles will be sourced or identified. No gray literature searches. Articles will be restricted to English or transcribed English, with no timeline restrictions, only human studies to be included, and no restrictions to study design	All literature evaluating pharmacological interventions for children and adults living with FASD	All age groups	Adults and children either diagnosed with FASD or who are at risk of having FASD	benefits and risks of psychotropic medications on patients (adults and children) diagnosed with FASD
Reid et al [3]	NI	Australia	32	No restrictions on the types of study designs<br>Inclusion: Nonpharmacological intervention studies that aim to improve an aspect of functioning<br>Exclusion: Studies that evaluate diagnostic services	Nonpharmacological interventions	all age groups	Any individuals with FASD	Improvements in functioning for people with FASD, e.g., adaptive, cognitive, self-regulation, social skills, behavior
Ordenewitz et al [4]	Sep 19	Germany	25	Intervention studies, randomized controlled trials (RCTs) for children and adolescents with FASD<br>Language: English, German, French<br>Date of publication: Since January 1, 2000	Any intervention	<18 y	Children and adolescents (<18 y), diagnosed with FASD (FAS, pFAS, ARND)	Effects on the affected CNS domain according to the German guideline for the diagnosis of FASD

 © 2025. Thieme. All rights reserved.

Results	Comments	Critical appraisal modified after AMSTAR-2
–Supporting Attachment and Family Wellness:<br>All interventions included caregiver components; Interventions were original and specifically designed for the PAE/FASD child–caregiver dyad and may be particularly (although not necessarily exclusively) impactful in early childhood. They had positive impacts on attachment and child adjustment, including improved relationships, enhanced caregiving experiences, and increased family functioning. They support a preventative model in which better bonding may aid the developmental process in the child and diminish the risk of adversity<br>–Building Skills and Strategies (Self-regulation, Behavioral Skills, Social Skills, Mental Health Literacy):<br>Nearly half of the interventions involved caregiver/teacher training. They were often conducted in middle childhood<br>Self-regulation and social skills strategies have the strongest evidence for use in children with PAE/FASD, and there is promising evidence for interventions to support the development of positive behavioral skills and strategies<br>Skill-building was not exclusive to the individual with PAE/FASD; in many cases, interventions also incorporated external support through facilitators, caregivers, teachers, or mentors<br>Importantly, these interventions led to improved indicators of mental health, suggesting that the acquisition of skills and strategies is one viable mechanism for individuals with FASD (and their families), to cope, interact, and feel better<br>–Responding to Risk and Reducing Harm (Substance Use, Justice Involvement):<br>In later adolescence and adulthood, as needs may become more complex, interventions shifted to a more responsive approach to mitigate risk and reduce harm<br>–Importance of caregivers and their active and intensive participation<br>–Combined, these approaches may reflect the components critical to integrated and interdependent care planning for individuals with PAE/FASD across the life course.	Most studies were RCTs ($n = 12$) and controlled clinical trials (CCTs; $n = 8$); 4 were case studies, 3 were case series, 3 were cohort (before and after) studies, one was a file review, one was an implementation study, and one was an exploratory study.	1. PICO: Yes (no comparator needed)<br>2. Protocol: Yes<br>3. Study selection: No<br>4. Search strategy: Yes<br>5. Selection in duplicate: Yes<br>6. Extraction in duplicate: Yes<br>7. Excluded studies: No<br>8. Included studies: Partial Yes<br>9. RoB: Yes; Yes<br>10. Funding: No<br>11. Meta-analysis method: NA<br>12. Meta-analysis RoB: NA<br>13. RoB in discussion: Yes<br>14. Heterogeneity: Yes<br>15. Publication bias: NA<br>16. Conflicts: Yes<br>Low RoB
–Hyperactivity and inattention: Inattention was found to respond better to Dextroamphetamine than Methylphenidate, but a high adverse event profile induced discontinuation. Atomoxetine may be useful in the inattention domain of FASD due to its noradrenergic stimulation effect<br>–Social skills: Stimulants were found to be less efficacious compared to second-generation neuroleptics, specifically in the domain of social skills. Stimulants showed comparatively poor response both as monotherapy and in combination with neuroleptics. Greater improvement was found with neuroleptics compared to those not prescribed neuroleptics (with and without combination with stimulants)<br>–Seizure disorders: Second-generation antipsychotics are used to treat complications of seizure disorders, as an adjunct therapy for Conduct Disorder, for disruptive behavior in children with low IQ, and for secondary disabilities associated with FASD<br>–Short-term aggressiveness: Risperidone has demonstrated strong benefits in the treatment of short-term aggressiveness in some research but too low evidence<br>–Appetite: Risperidone has the tendency to increase appetite<br>–Adverse effects: There is concern for long-term use of Risperidone because of the metabolic risk associated with most second-generation antipsychotics as well as having the	Very poor studies included: animal studies, not only patients with FASD, a study with 4 participants, a placebo group with 1 child, and no medication at all critical!<br>A standardized critical appraisal of the studies was done, but the risk of bias or level of evidence is not recorded for the studies included<br>Not all included studies are reported in the discussion<br>No real results/conclusion	1. PICO: Yes (no comparator needed)<br>2. Protocol: Yes<br>3. Study selection: No<br>4. Search strategy: Yes<br>5. Selection in duplicate: Yes<br>6. Extraction in duplicate: Yes<br>7. Excluded studies: No<br>8. Included studies: Partial No<br>9. RoB: Yes; No information (RoB is not documented!!)<br>10. Funding: No<br>11. Meta-analysis method: NA<br>12. Meta-analysis RoB: NA<br>13. RoB in discussion: No<br>14. Heterogeneity: No<br>15. Publication bias: NA<br>16. Conflicts: Yes

(*Continued*)

 © 2025. Thieme. All rights reserved.

(*Continued*)

Results	Comments	Critical appraisal modified after AMSTAR-2
potential for extrapyramidal symptoms and altering the dopaminergic system<br>–Depression: Antidepressants such as SSRI, SNRIs, NRIs, and TCAs are a class of medication prescribed for those diagnosed with FASD in the context of depression<br>–ADHD symptoms: SSRIs were reported as effective in treating ADHD symptoms when those coexist with behavior problems such as outbursts, aggression, and compulsive behaviors in children with FASD. Atomoxetine appears to be less effective than Methylphenidate in children with an IQ below 85. ADHD symptoms can be treated with a stimulant such as Adderall or Dexedrine<br>–In pediatric patients living with FASD, a lot of medications used are "off label"<br>–The choice of medication should be based on the most relevant diagnosis causing functional impairment or targeting two co-existing diagnoses.		Critical RoB because the studies included
–Developmental outcomes in infants: Mixed results: 1 study showed that following their intensive home visiting service, children with PAE scored in the average range on developmental tests. 1 study with a considerably stronger design found no effect of the home visiting service on the same measures of developmental outcome, with children scoring significantly below age-expected norms<br>–Self-regulation and attentional control (early to middle childhood): ALERT was shown to be effective in improving executive functioning and showed changes in grey matter volume in critical regions for self-regulations. A computerized progressive attention program (CPAP) showed a significant decrease in reaction times and distractibility and significant improvement in auditory sustained attention. Activities from the pay attention training protocol with additional visual search tasks showed significant improvements in nonverbal reasoning, auditory and visual sustained attention, and a trend for improved performance on alternating attention. A small study on cognitive control therapy (learning metacognitive skills) showed no gains in cognitive functioning promising results, but limited follow-up<br>–Specific skills: MILE was shown to be effective in improving math knowledge, parent-reported problem behavior, improved nonverbal reasoning, reading comprehension, and mathematics reasoning. CPAP showed improvements in math and reading fluency. A virtual reality game of fire/street safety showed significantly better knowledge of fire/street safety immediately and at follow-up (1 wk) and most children (72%) were able to generalize the information within a behavioral setting. Classroom-based literacy training showed improvements in specific language and literacy skills, but not in general scholastic skills. Experimental group rehearsal training showed an increase in digit span scores. A cover, copy, and compare spelling procedure showed an increase in the number of words spelled correctly. A motor skill training (FAST) could not affect cortisol levels<br>–Social skills in 3–12 y olds: Child Friendship Training (CFT) showed improvements in social skills and a decrease in hostile attribution (maintained at follow-up). Children with PAE can be treated in community settings if interventions are suitable. A community-based social skills group showed gains in parent-rated social skills. Strong evidence for structured programs that include children and parents in helping to improve social skills<br>–Parenting skills: Families moving forward program showed improvement in parental self-efficacy, parent needs, parent self-care, and a reduction in child behavior problems. Child	No study had a strong quality for selection bias, or blinding<br>19 had a strong study design (RCTs and controlled clinical trials)<br>27 used reliable and valid measures<br>17 had a strong for withdrawal/dropouts	1. PICO: Yes<br>2. Protocol: Yes<br>3. Study selection: No<br>4. Search strategy: partial yes<br>5. Selection in duplicate: Yes<br>6. Extraction in duplicate: No information<br>7. Excluded studies: No<br>8. Included studies: Yes<br>9. RoB: Yes<br>10. Funding: No<br>11. Meta-analysis method: NA<br>12. Meta-analysis RoB: NA<br>13. RoB in discussion: Yes<br>14. Heterogeneity: Yes<br>15. Publication bias: NA<br>16. Conflicts: Yes<br>Low RoB

 © 2025. Thieme. All rights reserved.

(*Continued*)

Results	Comments	Critical appraisal modified after AMSTAR-2
interaction therapy and parent-only parenting support and management programs were both effective in reducing child behavioral problems and parent stress. A group workshop, an internet program, and standardized written information are effective in increasing parental knowledge. The workshop and the written information are effective in improving child behavior. Parents show improved well-being from support in managing their children's behavior<br>–Support, education, and advocacy: Coaching Families (CF) was effective in decreasing family needs and caregiver stress and in increasing goals. Specialized FASD training for workers and foster caregivers showed a significant decline in the number of placement changes. Workshops for teachers showed improved classroom behavior. Key worker and parent support program that provides support, education, and liaison to existing intervention services showed a better understanding of FASD and increased emotional and practical support, but only a trend to improve caregiver stress, parenting-self-confidence, and child's behavior.		
Language/speech:<br>Language and literacy training (LLT): FASD-children and healthy control group improved. FASD-Children in the LLT group did catch up to their peers in some subtests regarding their skills in written letters, reading, and spelling of words and nonwords. There was a statistically significant improvement with respect to phonological and literacy skills in the FASD–LLT group compared to the FASD-control group<br>Learning/memory skills:<br>–Choline supplementation: no evidence for an effect on memory, executive function, and attention deficits. No effects on neurocognitive development. But younger participants (<4 y) improved in behavioral imitation tasks more than older participants (4–5 y). Follow-up: statistically significant effect of choline on nonverbal visual-spatial reasoning and nonverbal working memory compared to the placebo group<br>–Verbal rehearsal: no statistically significant differences between the experimental and the control group regarding memory of numbers<br>Executive functions:<br>–ALERT with parents: ALERT-group displayed statistically significant improvements in executive functioning and emotional functioning compared to the control group<br>–ALERT without parents: treatment effect on inhibition tasks in children and on parent-reported behavioral regulation. There were positive treatment effects on emotional control and performance in an inhibition task after the intervention<br>Arithmetic skills:<br>–MILE with parents: increase in parents' knowledge of FASD, caregiver advocacy, and behavioral regulation. Significantly less problematic behavior in their children was reported after the study. MILE-group showed greater gains in math performance than those in the control group. Follow-up: further betterment in mathematical skills in the MILE-group compared to control. An extension of the program to 15 wks did not lead to a more distinct treatment effect (math skills) compared to the 6-wks-program<br>–MILE without parents: Evidence for the effectiveness of the MILE intervention without	No quality analysis of included studies<br>But the quality of studies is very high.	1. PICO: Yes<br>2. Protocol: No<br>3. Study selection: No<br>4. Search strategy: partial yes<br>5. Selection in duplicate: No<br>6. Extraction in duplicate: No information<br>7. Excluded studies: No<br>8. Included studies: Yes<br>9. RoB: No<br>10. Funding: No<br>11. Meta-analysis method: NA<br>12. Meta-analysis RoB: NA<br>13. RoB in discussion: Yes<br>14. Heterogeneity: Yes<br>15. Publication bias: NA<br>16. Conflicts: Yes<br>Low RoB

(*Continued*)

 © 2025. Thieme. All rights reserved.

(*Continued*)

Results	Comments	Critical appraisal modified after AMSTAR-2
parent training<br>Attention:<br>–Sustained attention training: clear evidence for improvements in the intervention group compared to the controls in several domains of attention on direct child measures. But, both groups showed improvements in the teacher-rated domains in attention and executive functioning<br>Social skills and behavior:<br>–Workshop/community/online: workshop–and community-groups showed gains in the overall behavioral functioning of their children. All groups improved their knowledge of parents<br>–Step Up Intervention (SUI): A positive treatment effect for light/moderate drinkers in reducing alcohol consumption compared to controls was observed. For abstinent/infrequent drinkers, no changes in alcohol consumption were reported<br>–Families on Track (FoT) Program: Intervention increased caregivers' knowledge about FASD and their advocacy. Perceived needs of families decreased in both groups, with an observable trend for a larger decrease in the intervention group compared to the controls. A positive effect of FoT on parent-rated emotional regulation of the child was observable<br>–FAR strategy trough GoFAR game: Training in the FAR technique has benefits for the child. Parent training alone did not prove to be sufficient for behavioral changes of the child. Results indicated that parent engagement in the child's treatment is an integral part of behavioral improvements in children with FASD (less parent-reported disruptive behavior of the children). GoFAR group showed greater improvements in behavior than children in the FACELAND group. Positive treatment effects of the GoFAR program on attention and adaptive functioning<br>–Parent-assisted Children's Friendship Training (CFT): Results pointed toward enhancement in appropriate social skills (direct child measure) after the treatment that remained stable over a 3-mo period. Parent-rated social skills and problematic behavioral patterns of their child enhanced notably after treatment. Decline in hostile attributions in peer group entry scenarios, which were maintained at a 3-mo follow-up. Higher levels of self-regulation were associated with greater improvements in social skills after CFT. Treatment effects of the CFT program were further enhanced by neuroleptic medication, whereas stimulant medication or no medication at all did not ameliorate the outcomes. CFT-group benefitted from stronger increments in the knowledge of appropriate social behaviors and self-concept compared to children in the SOC group. Children with FASD demonstrated improvement in social skills and could be successfully integrated into social skills groups with children without PAE in a community mental health setting. No differences in parent reports of social skills between the CFT and SOC groups occurred. However, there was an observable trend that some children in SOC became worse in parent-reported social skills, while children in CFT generally displayed advances after treatment.		

Note: AMSTAR-2, a measurement tool to assess systematic reviews (2nd version).

 © 2025. Thieme. All rights reserved.

References for Supplementary Document 2

1 Flannigan K, Coons-Harding KD, Anderson T, et al. A systematic review of interventions to improve mental health and substance use outcomes for individuals with prenatal alcohol exposure and fetal alcohol spectrum disorder. Alcohol Clin Exp Res 2020;44(12):2401–2430

2 Mela M, Okpalauwaekwe U, Anderson T, et al. The utility of psychotropic drugs on patients with fetal alcohol spectrum disorder (FASD): a systematic review. Psychiatry Clin Psychopharmacol 2018;28(04):436–445

3 Reid N, Dawe S, Shelton D, et al. Systematic review of fetal alcohol spectrum disorder interventions across the life span. Alcohol Clin Exp Res 2015;39(12):2283–2295

4 Ordenewitz LK, Weinmann T, Schlüter JA, et al. Evidence-based interventions for children and adolescents with fetal alcohol spectrum disorders –a systematic review. Eur J Paediatr Neurol 2021;33:50–60

 © 2025. Thieme. All rights reserved.

Supplementary Document 3: Summary of Findings Tables (GRADE-Tables)

The quality of evidence for each outcome has been evaluated based on the GRADE criteria. However, due to variations in the composition of intervention and control groups, diverse test methodologies employed to assess intervention effects, differing methodological approaches, and inconsistent reporting across the included studies, a meaningful or standardized calculation of an effect estimator was not feasible. Consequently, no relative or anticipated absolute effects are presented in the subsequent tables.

 © 2025. Thieme. All rights reserved.

Supplementary Table D3.1 Summary of findings, drugs

Population[g]: Children with FASD or high prenatal alcohol exposure
Setting[g]: Unknown
Intervention[g]: Methylphenidate, stimulants, neuroleptics
Comparison[g]: Diverse drugs, no comparison group, placebo

Outcomes (relevance)	Number of participants (study design)[d]	Factors leading to quality downgrade					Factors leading to quality upgrade			Other factors to consider when making recom-mendations[c]	Certainty of the evidence—GRADE
		Risk of Bias[a]	Indirectness[b]	Inconsistency	Imprecision[b]	Publication bias[b]	Effect size[b]	Dose effect[b]	Residual plausible confounding[b]		
Epilepsy (critical)	$n = 10$ (1× control study)[1]	1× very high	No	No	No	No	No	No	No	1 systematic review; only 1 study; very small sample	Very low ⊕⊝⊝⊝
Social skills and behavior[e] (critical)	N[g,1–3]	2× low, 1× very high	No	Yes	No	No	No	No	No	2 systematic reviews; 13 studies; high data volume; only mild adverse effects	Moderate ⊕⊕⊕⊝
Attention[e] (critical)	$n > 125$ (1× RCT; 1× uncontrolled intervention study)[g,1,3]	1× low, 1× very high	No	Yes	No	No	No	No	No	1 systematic review; teilweise very kleine Stichproben ($n = 10$); unterschiedliche Art an Medikamenten	Low ⊕⊕⊝⊝
Adverse effects[f] (critical)	$n = 114$ (uncontrolled intervention study)[3]	1× low	No	Yes	No	No	No	No	No	Nur 1 Studie; keine Placebo-Gruppe; kein Fokus auf Adverse effects	Moderate ⊕⊕⊕⊝

[a]Assessment: randomized controlled trials: ROB 2; nonrandomized controlled trials: ROBINS-I; uncontrolled trials: adapted form of ROBINS-I; systematic reviews: AMSTAR-2.
[b]"Yes" if at least one publication fulfills this criterion.
[c]Some of the comments relate only to single publications.
[d]For systematic reviews, the number of participants and study designs of the relevant studies in the review are presented.
[e]Suboutcome of: improvement in the neuropsychological functions of children/adolescents with FASD.
[f]Avoidance of adverse effects of the interventions.
[g]Cannot be determined precisely due to the reporting of the systematic reviews.

 © 2025. Thieme. All rights reserved.

References for Supplementary Table D3.1

1. Mela M, Okpalauwaekwe U, Anderson T, et al. The utility of psychotropic drugs on patients with fetal alcohol spectrum disorder (FASD): a systematic review. Psychiatry Clin Psychopharmacol 2018;28(4):436–445
2. Ordenewitz LK, Weinmann T, Schlüter JA, et al. Evidence-based interventions for children and adolescents with fetal alcohol spectrum disorders - a systematic review. Eur J Paediatr Neurol 2021;33:50–60
3. Śmiarowska M, Brzuchalski B, Grzywacz E, et al. Influence of COMT (rs4680) and DRD2 (rs1076560, rs1800497) gene polymorphisms on safety and efficacy of methylphenidate treatment in children with fetal alcohol spectrum disorders. Int J Environ Res Public Health 2022;19(8):4479

 © 2025. Thieme. All rights reserved.

Supplementary Table D3.2 Summary of findings, supplements

Population: Children with FASD
Setting: At home
Intervention: Choline supplementation in different dosages
Comparison: Placebo

Outcomes (relevance)	Number of participants (study design)[d]	Factors leading to quality downgrade					Factors leading to quality upgrade			Other factors to consider when making recommendations[c]	Certainty of the evidence—GRADE
		Risk of Bias[a]	Indirectness[b]	Inconsistency	Imprecision[b]	Publication bias[b]	Effect size[b]	Dose effect[b]	Residual plausible confounding[b]		
Kognitive Leistung/ Intelligenz (critical)	*n* = 91 (2× RCT)[1,2]	2× low	No	No	No	No	Yes	No	No	Fishy body-odor as an adverse effect; only part of cognition; improvement only with latency; different test procedures; high lost-to-follow-up	High ⊕⊕⊕⊕
Executive functions[e] (critical)	*n* = 86 (2× RCT)[2,3]	2× low	No	No	Yes	No	No	No	No	Only 2 studies, each with different age of children, duration and dose of medication; 1 follow-up; fishy body-odor as adverse effect	High ⊕⊕⊕⊕
Learning and memory[e] (critical)	*n* = 168 (4× RCT)[1–4]	4× low	No	Yes	Yes	No	No	No	No	No direct effect, not even in long-term memory; improvement only in nonverbal working memory and only with a latency of a few years; fishy body-odor as an adverse effect	Moderate ⊕⊕⊕⊝
Attention[e] (critical)	*n* = 86 (2× RCT)[2,3]	2× low	No	Yes	No	No	Yes	No	No	High lost-to-follow-up; fishy body-odor as an adverse effect; positive effect only with latency	High ⊕⊕⊕⊕
Adverse effects[f] (critical)	*n* = 132 (3× RCT)[1–5]	3× low	No	Yes	No	No	No	Yes	No	Fishy body-odor as an adverse effect	High ⊕⊕⊕⊕

[a]Assessment: randomized controlled trials: ROB 2; nonrandomized controlled trials: ROBINS-I; uncontrolled trials: adapted form of ROBINS-I; systematic reviews: AMSTAR-2.
[b]"Yes" if at least one publication fulfills this criterion.
[c]Some of the comments relate only to single publications.
[d]For systematic reviews, the number of participants and study designs of the relevant studies in the review are presented.
[e]Suboutcome of: improvement in the neuropsychological functions of children/adolescents with FASD.
[f]Avoidance of adverse effects of the interventions.

 © 2025. Thieme. All rights reserved.

References for Supplementary Table D3.2

1. Wozniak JR, Fuglestad AJ, Eckerle JK, et al. Choline supplementation in children with fetal alcohol spectrum disorders: a randomized, double-blind, placebo-controlled trial. Am J Clin Nutr 2015;102(5):1113–1125
2. Wozniak JR, Fink BA, Fuglestad AJ, et al. Four-year follow-up of a randomized controlled trial of choline for neurodevelopment in fetal alcohol spectrum disorder. J Neurodev Disord 2020;12(1):9
3. Nguyen TT, Risbud RD, Mattson SN, Chambers CD, Thomas JD. Randomized, double-blind, placebo-controlled clinical trial of choline supplementation in school-aged children with fetal alcohol spectrum disorders. Am J Clin Nutr 2016;104(6):1683–1692
4. Wozniak JR, Fuglestad AJ, Eckerle JK, et al. Choline supplementation in children with fetal alcohol spectrum disorders has high feasibility and tolerability. Nutr Res 2013;33(11):897–904
5. Smith SM, Virdee MS, Eckerle JK, et al. Polymorphisms in SLC44A1 are associated with cognitive improvement in children diagnosed with fetal alcohol spectrum disorder: an exploratory study of oral choline supplementation. Am J Clin Nutr 2021;114(2):617–627

 © 2025. Thieme. All rights reserved.

Supplementary Table D3.3 Summary of findings, transcranial direct current stimulation (tDCS)

Population: Children with FASD
Setting: Clinical setting
Intervention: Transcranial direct current stimulation (tDCS)
Comparison: Sham

Outcomes (relevance)	Number of participant (study design)[d]	Factors leading to quality downgrade					Factors leading to quality upgrade			Other factors to consider when making recommendations[c]	Certainty of the evidence—GRADE
		Risk of Bias[a]	Indirectness[b]	Inconsistency	Imprecision[b]	Publication bias[b]	Effect size[b]	Dose effect[b]	Residual plausible confounding[b]		
Executive functions[e] (critical)	*n* = 38 (RCT)[1]	1× low	No	No	No	No	No	No	No	Only 1 study; high effort; potential harm outweighs potential benefit	High ⊕⊕⊕⊕
Learning and memory[e] (critical)	*n* = 38 (RCT)[1]	1× low	No	No	No	No	No	No	No	Only 1 study; high effort; potential harm outweighs potential benefit	High ⊕⊕⊕⊕
Attention[e] (critical)	*n* = 38 (RCT)[1]	1× low	No	No	No	No	No	No	No	Only 1 study; high effort; potential harm outweighs potential benefit	High ⊕⊕⊕⊕
Adverse effects[f] (critical)	*n* = 38 (RCT)[1]	1× low	No	No	No	No	No	No	No	Only 1 study; high effort; potential harm outweighs potential benefit	High ⊕⊕⊕⊕

[a]Assessment: randomized controlled trials: ROB 2; nonrandomized controlled trials: ROBINS-I; uncontrolled trials: adapted form of ROBINS-I; systematic reviews: AMSTAR-2.
[b]"Yes" if at least one publication fulfills this criterion.
[c]Some of the comments relate only to single publications.
[d]For systematic reviews, the number of participants and study designs of the relevant studies in the review are presented.
[e]Suboutcome of: improvement in the neuropsychological functions of children/adolescents with FASD.
[f]Avoidance of adverse effects of the interventions.

Reference for Supplementary Table D3.3

1. Boroda E, Krueger AM, Bansal P, et al. A randomized controlled trial of transcranial direct-current stimulation and cognitive training in children with fetal alcohol spectrum disorder. Brain Stimul 2020;13(4):1059–1068

 © 2025. Thieme. All rights reserved.

Supplementary Table D3.4 Summary of findings, somatosensory trainings

Population: Children with FASD
Setting: Unknown
Intervention: The Neurosequential Model of Therapeutics (NMT) to determine the intervention elements from Parent-Child Psychotherapy (CPP) and Mindful Parenting Education (MPE)
Comparison: No comparison group

Outcomes (relevance)	Number of participants (study design)[d]	Factors leading to quality downgrade					Factors leading to quality upgrade			Other factors to consider when making recommendations[c]	Certainty of the evidence—GRADE
		Risk of Bias[a]	Indirectness[b]	Inconsistency	Imprecision[b]	Publication bias[b]	Effect size[b]	Dose effect[b]	Residual plausible confounding[b]		
Development[e] (critical)	$n = 10$ (uncontrolled intervention study)[1]	1× high	No	Yes	Yes	No	Yes	No	No	Only 1 study; small sample; no follow-up; no differentiation between developmental subcategories	Very low ⊕⊝⊝⊝
Parental relief[f] (critical)	$n = 10$ (uncontrolled intervention study)[1]	1× high	No	Yes	Yes	No	Yes	No	No	Only 1 study; small sample; no follow-up	Very low ⊕⊝⊝⊝
Knowledge acquisition[g] (critical)	$n = 10$ (uncontrolled intervention study)[1]	1× high	No	Yes	Yes	No	Yes	No	No	Only 1 study; small sample; no follow-up	Very low ⊕⊝⊝⊝

[a]Assessment: randomized controlled trials: ROB 2; nonrandomized controlled trials: ROBINS-I; uncontrolled trials: adapted form of ROBINS-I; systematic reviews: AMSTAR-2.
[b]"Yes" if at least one publication fulfills this criterion.
[c]Some of the comments relate only to single publications.
[d]For systematic reviews, the number of participants and study designs of the relevant studies in the review are presented.
[e]Suboutcome of: improvement in the neuropsychological functions of children/adolescents with FASD.
[f]Relief for caregivers (biological, foster and adoptive parents, caregivers) and improving the quality of life of the entire family/institution affected family/institution.
[g]Improving knowledge of the deviant state of health/disorder/disability and improvement of insight into the illness.

Reference for Supplementary Table D3.4

1. Zarnegar Z, Hambrick EP, Perry BD, Azen SP, Peterson C. Clinical improvements in adopted children with fetal alcohol spectrum disorders through neurodevelopmentally informed clinical intervention: A pilot study. Clin Child Psychol Psychiatry 2016;21(4):551–567

 © 2025. Thieme. All rights reserved.

Supplementary Table D3.5 Summary of findings, balance trainings

Population: Children with FASD
Setting: University, laboratory, at home
Intervention: The virtual reality system "Sensorimotor Training to Affect Balance, Engagement and Learning" (STABEL)
Comparison: Inactive comparison group

Outcomes (relevance)	Number of participant (study design)[d]	Factors leading to quality downgrade					Factors leading to quality upgrade			Other factors to consider when making recommendations[c]	Certainty of the evidence—GRADE
		Risk of Bias[a]	Indirectness[b]	Inconsistency	Imprecision[b]	Publication bias[b]	Effect size[b]	Dose effect[b]	Residual plausible confounding[b]		
Fine/graphomotor skills or gross motor coordination[e] (critical)	$n = 45$ (1× nonrandomized control study; 1× uncontrolled study)[1,2]	1× low, 1× high	No	Yes	Yes	No	No	No	No	Possible at home; little time required; uncertain clinical significance; worsening of postural stability and sensory attention (fatigue?); no follow-up	Low ⊕⊕⊖⊖

[a]Assessment: randomized controlled trials: ROB 2; nonrandomized controlled trials: ROBINS-I; uncontrolled trials: adapted form of ROBINS-I; systematic reviews: AMSTAR-2.
[b]"Yes" if at least one publication fulfills this criterion.
[c]Some of the comments relate only to single publications.
[d]For systematic reviews, the number of participants and study designs of the relevant studies in the review are presented.
[e]Suboutcome of: improvement in the neuropsychological functions of children/adolescents with FASD.

References for Supplementary Table D3.5

1. McCoy SW, Jirikowic T, Price R, et al. Virtual sensorimotor balance training for children with fetal alcohol spectrum disorders: feasibility study. Phys Ther 2015;95(11):1569–1581
2. Jirikowic T, Westcott McCoy S, Price R, Ciol MA, Hsu LY, Kartin D. Virtual sensorimotor training for balance: pilot study results for children with fetal alcohol spectrum disorders. Pediatr Phys Ther 2016;28(4):460–468

 © 2025. Thieme. All rights reserved.

Supplementary Table D3.6 Summary of findings, language trainings

Population: Children with FASD
Setting[f]: Unknown
Intervention: Language and literacy training
Comparison: Inactive comparison group, children without FASD

Outcomes (relevance)	Number of participant (study design)[d]	Factors leading to quality downgrade					Factors leading to quality upgrade			Other factors to consider when making recommendations[c]	Certainty of the evidence—GRADE
		Risk of Bias[a]	Indirectness[b]	Inconsistency	Imprecision[b]	Publication bias[b]	Effect size[b]	Dose effect[b]	Residual plausible confounding[b]		
Language[e] (critical)	$n = 59$ (control study)[1]	1× moderate	No	No	No	No	No	No	No	Systematic review with only 1 study; outcome is not receptive/expressive language	Moderate ⊕⊕⊕⊝

[a]Assessment: randomized controlled trials: ROB 2; nonrandomized controlled trials: ROBINS-I; uncontrolled trials: adapted form of ROBINS-I; systematic reviews: AMSTAR-2.
[b]"Yes" if at least one publication fulfills this criterion.
[c]Some of the comments relate only to single publications.
[d]For systematic reviews, the number of participants and study designs of the relevant studies in the review are presented.
[e]Suboutcome of: improvement in the neuropsychological functions of children/adolescents with FASD.
[f]Cannot be determined precisely due to the reporting of the systematic reviews.

Reference for Supplementary Table D3.6

1. Ordenewitz LK, Weinmann T, Schlüter JA, et al. Evidence-based interventions for children and adolescents with fetal alcohol spectrum disorders - a systematic review. Eur J Paediatr Neurol 2021;33:50–60

Neuropediatrics © 2025. Thieme. All rights reserved.

Supplementary Table D3.7 Summary of findings, training for promoting mathematical thinking

Population: Children with FASD
Setting: School, at home, clinical setting
Intervention: The Math Interactive Learning Experience (MILE) Program
Comparison: The Social Skills Improvement System Intervention, inactive comparison group, parental education

Outcomes (relevance)	Number of participant (study design)[d]	Factors leading to quality downgrade					Factors leading to quality upgrade			Other factors to consider when making recommendations[c]	Certainty of the evidence—GRADE
		Risk of Bias[a]	Indirectness[b]	Inconsistency	Imprecision[b]	Publication bias[b]	Effect size[b]	Dose effect[b]	Residual plausible confounding[b]		
Spatial-visual perception or spatial-constructive abilities[e] (critical)	$n = 28$ (control study)[1]	1× moderate	No	Yes	No	No	No	No	No	Only 1 study; no follow-up	Moderate ⊕⊕⊕⊝
Executive functions[e] (critical)	$n = 28$ (control study)[1]	1× moderate	No	Yes	No	No	No	No	No	Only 1 study, no follow-up; No clear advantage of an intervention	Moderate ⊕⊕⊕⊝
Mathematical skills[e]	$n = 190$[h] (3× RCT, 1× control study)[1–4]	2× low, 2× moderate	Yes	No	No	No	Yes	No	No	2 systematic reviews; 4 studies; 2× same sample; comparison with skills training; 2× follow-up; with and without parental involvement; dependence on individual factors unclear	High ⊕⊕⊕⊕
Learning and memory[e] (critical)	$n = 60$ (RCT)[3]	1× moderate	No	Yes	Yes	No	No	No	No	Only 1 study; only parent assessment of the child's learning ability	Low ⊕⊕⊝⊝
Attention[e] (critical)	$n = 28$ (control study)[1]	1× moderate	No	Yes	Yes	No	No	No	Yes	Only 1 study; no follow-up; no clear advantage of an intervention	Very low ⊕⊝⊝⊝
Learning and application of knowledge[f] (critical)	$n = 60$ (RCT)[3]	1× moderate	No	Yes	Yes	No	No	No	No	Only 1 study; only subjective assessment of learning behavior; transferability to everyday life unclear	Low ⊕⊕⊝⊝
Knowledge acquisition[g] (critical)	$n = 60$ (RCT)[3]	1× moderate	No	Yes	Yes	No	No	No	No	Only 1 study; intervention-specific effect unclear	Moderate ⊕⊕⊕⊝

[a]Assessment: randomized controlled trials: ROB 2; nonrandomized controlled trials: ROBINS-I; uncontrolled trials: adapted form of ROBINS-I; systematic reviews: AMSTAR-2.
[b]"Yes" if at least one publication fulfills this criterion.
[c]Some of the comments relate only to single publications.
[d]For systematic reviews, the number of participants and study designs of the relevant studies in the review are presented.
[e]Suboutcome of: improvement in the neuropsychological functions of children/adolescents with FASD.
[f]Suboutcome of: improving the participation of children/young people with FASD.
[g]Improving knowledge of the deviant state of health/disorder/disability and improvement of insight into the illness.
[h]Cannot be determined precisely due to overlaps in the study population.

 © 2025. Thieme. All rights reserved.

References for Supplementary Table D3.7

1. Kully-Martens K, Pei J, Kable J, Coles CD, Andrew G, Rasmussen C. Mathematics intervention for children with fetal alcohol spectrum disorder: a replication and extension of the math interactive learning experience (MILE) program. Res Dev Disabil 2018;78:55–65
2. Ordenewitz LK, Weinmann T, Schlüter JA, et al. Evidence-based interventions for children and adolescents with fetal alcohol spectrum disorders - a systematic review. Eur J Paediatr Neurol 2021;33:50–60
3. Kable JA, Taddeo E, Strickland D, Coles CD. Community translation of the math interactive learning experience program for children with FASD. Res Dev Disabil 2015;39:1–11
4. Reid N, Dawe S, Shelton D, et al. Systematic review of fetal alcohol spectrum disorder interventions across the life span. Alcohol Clin Exp Res 2015;39(12):2283–2295

 © 2025. Thieme. All rights reserved.

Supplementary Table D3.8 Summary of findings, Serious Games

Population: Children with FASD
Setting: School
Intervention: The Caribbean Quest, Virtual Reality Game for fire safety, Virtual Reality Game for traffic safety
Comparison: No comparison group, comparison between two Serious Games

Outcomes (relevance)	Number of participants (study design)[d]	Factors leading to quality downgrade					Factors leading to quality upgrade			Other factors to consider when making recommendations[c]	Certainty of the evidence—GRADE
		Risk of Bias[a]	Indirectness[b]	Inconsistency	Imprecision[b]	Publication bias[b]	Effect size[b]	Dose effect[b]	Residual plausible confounding[b]		
Mathematical skills[e] (critical)	$n=17$ (uncontrolled intervention study)[1]	1× moderate	No	Yes	Yes	No	No	No	No	Only 1 study; small sample; group consisted of children with FASD and ASD—no group difference calculated; only subjective improvement	Very low ⊕⊝⊝⊝
Learning and memory[e] (critical)	$n=17$ (uncontrolled intervention study)[1]	1× moderate	No	Yes	No	No	No	No	No	Only 1 study; small sample; group consisted of children with FASD and ASD—no group difference calculated	Very low ⊕⊝⊝⊝
Danger to self/others[f] (critical)	$n=21$ (2× control study)[1]	1× low	No	No	No	No	No	No	No	1 systematic review; very small sample	Low ⊕⊕⊝⊝
Learning and application of knowledge[g] (critical)	$n=17$ (uncontrolled intervention study)[1]	1× moderate	No	Yes	No	No	No	No	No	Only 1 study; small sample; group consisted of children with FASD and ASD—no group difference calculated; only subjective, qualitative assessment of teachers	Very low ⊕⊝⊝⊝

[a]Assessment: randomized controlled trials: ROB 2; nonrandomized controlled trials: ROBINS-I; uncontrolled trials: adapted form of ROBINS-I; systematic reviews: AMSTAR-2.
[b]"Yes" if at least one publication fulfills this criterion.
[c]Some of the comments relate only to single publications.
[d]For systematic reviews, the number of participants and study designs of the relevant studies in the review are presented.
[e]Suboutcome of: improvement in the neuropsychological functions of children/adolescents with FASD.
[f]Suboutcome of: reduction of complications/secondary diseases.
[g]Suboutcome of: improving the participation of children/young people with FASD.

 © 2025. Thieme. All rights reserved.

Reference for Supplementary Table D3.8

1. Kerns KA, Macoun S, MacSween J, Pei J, Hutchison M. Attention and working memory training: a feasibility study in children with neurodevelopmental disorders. Appl Neuropsychol Child 2017;6(2):120–137

 © 2025. Thieme. All rights reserved.

Supplementary Table D3.9 Summary of findings, neurocognitive trainings

Population: Children with FASD
Setting[g]: Clinical setting, at home, university, community setting
Intervention: (Adapted) Alert-program with/without parental training, GoFAR, The Caribbean Quest; Computerised Progressive Attention Program (CPAP), training based on Pay Attention Training Protocol
Comparison: Inactive comparison group, waiting list, training for emotional recognition (FACELAND), no comparison group

Outcomes (relevance)	Number of participant (study design)[d]	Factors leading to quality downgrade					Factors leading to quality upgrade			Other factors to consider when making recommendations[c]	Certainty of the evidence—GRADE
		Risk of Bias[a]	Indirectness[b]	Inconsistency	Imprecision[b]	Publication bias[b]	Effect size[b]	Dose effect[b]	Residual plausible confounding[b]		
Executive functions[e] (critical)	$n = 151$ (1× RCT, 2× control study)[1–3]	2× moderate, 1× high	Yes	No	Yes	No	Yes	No	Yes	3 studies; applicability to everyday life unclear; 2 studies by the same working group; large sample	Moderate ⊕⊕⊕⊝
Attention[e] (critical)	$n = 105$ (2× RCT, 2× control study 1× uncontrolled intervention study)[4–8]	2× low, 3× moderate	Yes	Yes	Yes	No	Yes	No	No	2 systematic reviews; 5 studies; overlap with other therapies; influence of parents unclear	Moderate ⊕⊕⊕⊝
Quality of life[f] (critical)	$n = 27$ (RCT)[9]	1× moderate	Yes	Yes	No	No	No	No	No	Nur 1 Studie; nur allgemeine Beeinträchtigung ermittelt	Low ⊕⊕⊝⊝

[a]Assessment: randomized controlled trials: ROB 2; nonrandomized controlled trials: ROBINS-I; uncontrolled trials: adapted form of ROBINS-I; systematic reviews: AMSTAR-2.
[b]"Yes" if at least one publication fulfills this criterion.
[c]Some of the comments relate only to single publications.
[d]For systematic reviews, the number of participants and study designs of the relevant studies in the review are presented.
[e]Suboutcome of: improvement in the neuropsychological functions of children/adolescents with FASD.
[f]Improving the quality of life of children/adolescents with FASD.
[g]Cannot be determined precisely due to the reporting of the systematic reviews.

 © 2025. Thieme. All rights reserved.

References for Supplementary Table D3.9

1. Nash K, Stevens S, Greenbaum R, Weiner J, Koren G, Rovet J. Improving executive functioning in children with fetal alcohol spectrum disorders. Child Neuropsychol 2015;21 (2):191–209
2. Soh DW, Skocic J, Nash K, Stevens S, Turner GR, Rovet J. Self-regulation therapy increases frontal gray matter in children with fetal alcohol spectrum disorder: evaluation by voxel-based morphometry. Front Hum Neurosci 2015;9:108
3. Wells AM, Chasnoff IJ, Schmidt CA, Telford E, Schwartz LD. Neurocognitive habilitation therapy for children with fetal alcohol spectrum disorders: an adaptation of the alert program. Am J Occup Ther 2012;66(1):24–34
4. Kerns KA, Macoun S, MacSween J, Pei J, Hutchison M. Attention and working memory training: a feasibility study in children with neurodevelopmental disorders. Appl Neuropsychol Child 2017;6(2):120–137
5. Ordenewitz LK, Weinmann T, Schlüter JA, et al. Evidence-based interventions for children and adolescents with fetal alcohol spectrum disorders - a systematic review. Eur J Paediatr Neurol 2021;33:50–60
6. Reid N, Dawe S, Shelton D, et al. Systematic review of fetal alcohol spectrum disorder interventions across the life span. Alcohol Clin Exp Res 2015;39(12):2283–2295
7. Kable JA, Taddeo E, Strickland D, Coles CD. Improving FASD Children's self-regulation: piloting phase 1 of the GoFAR intervention. Child Fam Behav Ther 2016;38 (2):124–141
8. Coles CD, Kable JA, Taddeo E, Strickland D. GoFAR: improving attention, behavior and adaptive functioning in children with fetal alcohol spectrum disorders: brief report. Dev Neurorehabil 2018;21(5):345–349
9. Petrenko CLM, Pandolfino ME, Robinson LK. Findings from the families on track intervention pilot trial for children with fetal alcohol spectrum disorders and their families. Alcohol Clin Exp Res 2017;41(7):1340–1351

 © 2025. Thieme. All rights reserved.

Supplementary Table D3.10 Summary of findings, emotion regulation trainings

**Population**: Children with FASD, Children with high prenatal alcohol exposure<br>**Setting**: Clinical setting, at home, university, community setting<br>**Intervention**: GoFAR, Alert, The Caribbean Quest, Families on Track<br>**Comparison**: Inactive comparison group, waiting list, training for emotion recognition (FACELAND), no comparison group, feedback from caregivers											
**Outcomes (relevance)**	**Number of participant (study design)[d]**	**Factors leading to quality downgrade**					**Factors leading to quality upgrade**			**Other factors to consider when making recommendations[c]**	**Certainty of the evidence—GRADE**
		**Risk of Bias[a]**	**Indirectness[b]**	**Inconsistency**	**Imprecision[b]**	**Publication bias[b]**	**Effect size[b]**	**Dose effect[b]**	**Residual plausible confounding[b]**		
Social skills and behavior[e] (critical)	$n = 259$ (6× RCT, 1× control study, 1× uncontrolled intervention study)[f1–8]	8× moderate	No	Yes	No	No	Yes	No	Yes	8 studies; large amount of data; follow-ups; long-term effect questionable; 3× same sample; 2× same sample; dependence on individual factors unclear	High ⊕⊕⊕⊕

[a]Assessment: randomized controlled trials: ROB 2; nonrandomized controlled trials: ROBINS-I; uncontrolled trials: adapted form of ROBINS-I; systematic reviews: AMSTAR-2.
[b]"Yes" if at least one publication fulfills this criterion.
[c]Some of the comments relate only to single publications.
[d]For systematic reviews, the number of participants and study designs of the relevant studies in the review are presented.
[e]Suboutcome of: improvement in the neuropsychological functions of children/adolescents with FASD.
[f]Cannot be determined precisely due to overlaps in the study population.

 © 2025. Thieme. All rights reserved.

References for Supplementary Table D3.10

1. Kerns KA, Macoun S, MacSween J, Pei J, Hutchison M. Attention and working memory training: a feasibility study in children with neurodevelopmental disorders. Appl Neuropsychol Child 2017;6(2):120–137
2. Nash K, Stevens S, Greenbaum R, Weiner J, Koren G, Rovet J. Improving executive functioning in children with fetal alcohol spectrum disorders. Child Neuropsychol 2015;21 (2):191–209
3. Wells AM, Chasnoff IJ, Schmidt CA, Telford E, Schwartz LD. Neurocognitive habilitation therapy for children with fetal alcohol spectrum disorders: an adaptation of the alert program. Am J Occup Ther 2012;66(1):24–34
4. Kable JA, Taddeo E, Strickland D, Coles CD. Improving FASD children's self-regulation: piloting phase 1 of the GoFAR intervention. Child Fam Behav Ther 2016;38(2):124–141
5. Coles CD, Kable JA, Taddeo E, Strickland D. GoFAR: improving attention, behavior and adaptive functioning in children with fetal alcohol spectrum disorders: brief report. Dev Neurorehabil 2018;21(5):345–349
6. Coles CD, Kable JA, Taddeo E, Strickland DC. A metacognitive strategy for reducing disruptive behavior in children with fetal alcohol spectrum disorders: GoFAR pilot. Alcohol Clin Exp Res 2015;39(11):2224–2233
7. Petrenko CLM, Pandolfino ME, Robinson LK. Findings from the families on track intervention pilot trial for children with fetal alcohol spectrum disorders and their families. Alcohol Clin Exp Res 2017;41(7):1340–1351
8. Petrenko CLM, Demeusy EM, Alto ME. Six-month follow-up of the families on track intervention pilot trial for children with fetal alcohol spectrum disorders and their families. Alcohol Clin Exp Res 2019;43(10):2242–2254

 © 2025. Thieme. All rights reserved.

Supplementary Table D3.11 Summary of findings, social skills trainings

Population: Children with FASD
Setting: Clinical setting
Intervention: Children's Friendship Training with/without neuroleptics
Comparison: Standard care

Outcomes (relevance)	Number of participant (study design)[d]	Factors leading to quality downgrade					Factors leading to quality upgrade			Other factors to consider when making recommendations[c]	Certainty of the evidence—GRADE
		Risk of Bias[a]	Indirectness[b]	Inconsistency	Imprecision[b]	Publication bias[b]	Effect size[b]	Dose effect[b]	Residual plausible confounding[b]		
Social skills and behavior[e] (critical)	$n = 567$[f] (6× Control study)[1–4]	3× low, 1× moderate	No	Yes	Yes	No	Yes	No	Yes	2 systematic reviews; 6 studies; partly the same samples; good feasibility; low effort; large sample	Moderate ⊕⊕⊕⊖

[a]Assessment: randomized controlled trials: ROB 2; nonrandomized controlled trials: ROBINS-I; uncontrolled trials: adapted form of ROBINS-I; systematic reviews: AMSTAR-2.
[b]"Yes" if at least one publication fulfills this criterion.
[c]Some of the comments relate only to single publications.
[d]For systematic reviews, the number of participants and study designs of the relevant studies in the review are presented.
[e]Suboutcome of: improvement in the neuropsychological functions of children/adolescents with FASD.
[f]Cannot be determined precisely due to overlaps in the study population.

References for Supplementary Table D3.11

1. Ordenewitz LK, Weinmann T, Schlüter JA, et al. Evidence-based interventions for children and adolescents with fetal alcohol spectrum disorders - a systematic review. Eur J Paediatr Neurol 2021;33:50–60
2. Reid N, Dawe S, Shelton D, et al. Systematic review of fetal alcohol spectrum disorder interventions across the life span. Alcohol Clin Exp Res 2015;39(12):2283–2295
3. O'Connor MJ, Laugeson EA, Mogil C, et al. Translation of an evidence-based social skills intervention for children with prenatal alcohol exposure in a community mental health setting. Alcohol Clin Exp Res 2012;36(1):141–152
4. Regehr E. The Impact of an Intervention on Social Skills of Young Children with Prenatal Alcohol Exposure [Master's Thesis, University of Alberta]. Alberta. Accessed 2015 at: https://dx.doi.org/10.7939/r3b56dc77

 © 2025. Thieme. All rights reserved.

Supplementary Table D3.12 Summary of findings, neurocognitive trainings combined with parental trainings

Population: Children/adolescents with FASD and their caregivers
Setting: University, clinical setting
Intervention: Project Step-up, GoFAR, Alert, Children's Friendship Training, Families on Track
Comparison: Written information, FACELAND, inactive comparison group, standard care, feedback from caregivers

Outcomes (relevance)	Number of participant (study design)[d]	Factors leading to quality downgrade					Factors leading to quality upgrade			Other factors to consider when making recommendations[c]	Certainty of the evidence—GRADE
		Risk of Bias[a]	Indirectness[b]	Inconsistency	Imprecision[b]	Publication bias[b]	Effect size[b]	Dose effect[b]	Residual plausible confounding[b]		
Attention[e] (critical)	$n=60$ (2× RCT)[1,2]	2× moderate	No	Yes	Yes	No	Yes	No	No	Only 2 studies; 2× same study population; small group sizes; high parental satisfaction	Moderate ⊕⊕⊕⊝
Risky alcohol/ drug consumption[f] (critical)	$n=54$[h] (2× RCT)[3,4]	1× low, 1× moderate	No	Yes	No	No	Yes	No	Yes	2× same study; follow-up; good feasibility; high satisfaction	High ⊕⊕⊕⊕
Interpersonal interaction and relationship[g] (critical)	$n=145$ (1× RCT, 1× control study)[5,6]	2× moderate	No	Yes	No	No	Yes	No	Yes	2 studies; large sample; long-term effects unclear; transferability unclear	Moderate ⊕⊕⊕⊝

[a]Assessment: randomized controlled trials: ROB 2; nonrandomized controlled trials: ROBINS-I; uncontrolled trials: adapted form of ROBINS-I; systematic reviews: AMSTAR-2.
[b]"Yes" if at least one publication fulfills this criterion.
[c]Some of the comments relate only to single publications.
[d]For systematic reviews, the number of participants and study designs of the relevant studies in the review are presented.
[e]Suboutcome of: improvement in the neuropsychological functions of children/adolescents with FASD.
[f]Suboutcome of: reduction of complications/secondary diseases.
[g]Suboutcome of: improving the participation of children/young people with FASD.
[h]Cannot be determined precisely due to overlaps in the study population.

 © 2025. Thieme. All rights reserved.

References for Supplementary Table D3.12

1. Kable JA, Taddeo E, Strickland D, Coles CD. Improving FASD children's self-regulation: piloting phase 1 of the GoFAR intervention. Child Fam Behav Ther 2016;38(2):124–141
2. Coles CD, Kable JA, Taddeo E, Strickland D. GoFAR: improving attention, behavior and adaptive functioning in children with fetal alcohol spectrum disorders: brief report. Dev Neurorehabil 2018;21(5):345–349
3. O'Connor MJ, Quattlebaum J, Castañeda M, Dipple KM. Alcohol intervention for adolescents with fetal alcohol spectrum disorders: project step up, a treatment development study. Alcohol Clin Exp Res 2016;40(8):1744–1751
4. Flannigan K, Coons-Harding KD, Anderson T, et al. A systematic review of interventions to improve mental health and substance use outcomes for individuals with prenatal alcohol exposure and fetal alcohol spectrum disorder. Alcohol Clin Exp Res 2020;44(12):2401–2430
5. Wells AM, Chasnoff IJ, Schmidt CA, Telford E, Schwartz LD. Neurocognitive habilitation therapy for children with fetal alcohol spectrum disorders: an adaptation of the alert program. Am J Occup Ther 2012;66(1):24–34
6. O'Connor MJ, Laugeson EA, Mogil C, et al. Translation of an evidence-based social skills intervention for children with prenatal alcohol exposure in a community mental health setting. Alcohol Clin Exp Res 2012;36(1):141–152

 © 2025. Thieme. All rights reserved.

Supplementary Table D3.13 Summary of findings, emotion regulation training combined with parental trainings

Population: Children with FASD, Children with high prenatal alcohol exposure
Setting: Clinical setting, at home, university, community setting
Intervention: GoFAR, Alert, Families on Track
Comparison: Inactive comparison group, waiting list, emotion regulation training (FACELAND), feedback from caregivers

Outcomes (relevance)	Number of participant (study design)[d]	Factors leading to quality downgrade					Factors leading to quality upgrade			Other factors to consider when making recommendations[c]	Certainty of the evidence—GRADE
		Risk of Bias[a]	Indirectness[b]	Inconsistency	Imprecision[b]	Publication bias[b]	Effect size[b]	Dose effect[b]	Residual plausible confounding[b]		
Social skills and behavior[e] (critical)	$n = 217^{g}$ (6× RCT)[1–6]	6× moderate	No	Yes	No	No	Yes	No	No	6 studies; large amount of data; follow-ups; long-term effect questionable; partly same samples; dependence on individual factors unclear	High ⊕⊕⊕⊕
Knowledge acquisition[f] (critical)	$n = 51^{g}$ (2× RCT)[5,6]	2× moderate	No	Yes	No	No	Yes	No	No	2× same sample; follow-up; long-term effects	Moderate ⊕⊕⊕⊝

[a]Assessment: randomized controlled trials: ROB 2; nonrandomized controlled trials: ROBINS-I; uncontrolled trials: adapted form of ROBINS-I; systematic reviews: AMSTAR-2.
[b]"Yes" if at least one publication fulfills this criterion.
[c]Some of the comments relate only to single publications.
[d]For systematic reviews, the number of participants and study designs of the relevant studies in the review are presented.
[e]Suboutcome of: improvement in the neuropsychological functions of children/adolescents with FASD.
[f]Improving knowledge of the deviant state of health/disorder/disability and improvement of insight into the illness.
[g]Cannot be determined precisely due to overlaps in the study population.

 © 2025. Thieme. All rights reserved.

References for Supplementary Table D3.13

1. Wells AM, Chasnoff IJ, Schmidt CA, Telford E, Schwartz LD. Neurocognitive habilitation therapy for children with fetal alcohol spectrum disorders: an adaptation of the alert program. Am J Occup Ther 2012;66(1):24–34
2. Kable JA, Taddeo E, Strickland D, Coles CD. Improving FASD children's self-regulation: piloting phase 1 of the GoFAR intervention. Child Fam Behav Ther 2016;38(2):124–141
3. Coles CD, Kable JA, Taddeo E, Strickland D. GoFAR: improving attention, behavior and adaptive functioning in children with fetal alcohol spectrum disorders: brief report. Dev Neurorehabil 2018;21(5):345–349
4. Coles CD, Kable JA, Taddeo E, Strickland DC. A metacognitive strategy for reducing disruptive behavior in children with fetal alcohol spectrum disorders: GoFAR pilot. Alcohol Clin Exp Res 2015;39(11):2224–2233
5. Petrenko CLM, Pandolfino ME, Robinson LK. Findings from the families on track intervention pilot trial for children with fetal alcohol spectrum disorders and their families. Alcohol Clin Exp Res 2017;41(7):1340–1351
6. Petrenko CLM, Demeusy EM, Alto ME. Six-month follow-up of the families on track intervention pilot trial for children with fetal alcohol spectrum disorders and their families. Alcohol Clin Exp Res 2019;43(10): 2242–2254

 © 2025. Thieme. All rights reserved.

Supplementary Table D3.14 Summary of findings, social skills trainings combined with parental trainings

Population: Children with FASD and their caregivers
Setting: Clinical setting
Intervention: Children's Friendship Training with/without neuroleptics
Comparison: Standard care

Outcomes (relevance)	Number of participant (study design)[d]	Factors leading to quality downgrade					Factors leading to quality upgrade			Other factors to consider when making recommendations[c]	Certainty of the evidence—GRADE
		Risk of Bias[a]	Indirectness[b]	Inconsistency	Imprecision[b]	Publication bias[b]	Effect size[b]	Dose effect[b]	Residual plausible confounding[b]		
Social skills and behavior[e] (critical)	n = ca. 567[f] (6× control study)[1–4]	3× low, 1× moderate	No	Yes	Yes	No	Yes	No	Yes	2 systematic reviews; 6 studies; partly same samples; good feasibility; low effort; large sample	Moderate ⊕⊕⊕⊖

[a]Assessment: randomized controlled trials: ROB 2; nonrandomized controlled trials: ROBINS-I; uncontrolled trials: adapted form of ROBINS-I; systematic reviews: AMSTAR-2.
[b]"Yes" if at least one publication fulfills this criterion.
[c]Some of the comments relate only to single publications.
[d]For systematic reviews, the number of participants and study designs of the relevant studies in the review are presented.
[e]Suboutcome of: improvement in the neuropsychological functions of children/adolescents with FASD.
[f]Cannot be determined precisely due to overlaps in the study population.

References for Supplementary Table D3.14

1. Ordenewitz LK, Weinmann T, Schlüter JA, et al. Evidence-based interventions for children and adolescents with fetal alcohol spectrum disorders - a systematic review. Eur J Paediatr Neurol 2021;33:50–60
2. Reid N, Dawe S, Shelton D, et al. Systematic review of fetal alcohol spectrum disorder interventions across the life span. Alcohol Clin Exp Res 2015;39(12):2283–2295
3. O'Connor MJ, Laugeson EA, Mogil C, et al. Translation of an evidence-based social skills intervention for children with prenatal alcohol exposure in a community mental health setting. Alcohol Clin Exp Res 2012;36(1):141–152
4. Regehr E. The Impact of an Intervention on Social Skills of Young Children with Prenatal Alcohol Exposure [Master's Thesis, University of Alberta]. Alberta. Accessed 2015 at: https://dx.doi.org/10.7939/r3b56dc77

 © 2025. Thieme. All rights reserved.

Supplementary Table D3.15 Summary of findings, psychoeducation of parents/caregivers

Population: Children with FASD and their caregivers
Setting: At home, clinical setting
Intervention: Written information, group workshops, online workshop, GoFAR
Comparison: Comparison between different forms, FACELAND, inactive comparison group

Outcomes (relevance)	Number of participant (study design)[d]	Factors leading to quality downgrade					Factors leading to quality upgrade			Other factors to consider when making recommendations[c]	Certainty of the evidence—GRADE
		Risk of Bias[a]	Indirectness[b]	Inconsistency	Imprecision[b]	Publication bias[b]	Effect size[b]	Dose effect[b]	Residual plausible confounding[b]		
Social skills and behavior[e] (critical)	$n = 59$ (RCT)[1]	Moderate	No	Yes	Yes	No	Yes	No	Yes	Only 1 study; good applicability; transferable to other countries; low effort, low costs	Moderate ⊕⊕⊕⊝
Domestic life[f] (critical)	$n = 30$ (RCT)[2]	1× moderate	No	Yes	No	No	No	No	No	Only 1 study; good applicability; transferable to other countries; low effort, low costs	Moderate ⊕⊕⊕⊝
Parental relief[g] (critical)	$n = 231$[i] (2× RCT, 1× uncontrolled study)[3–5]	3× moderate	No	Yes	No	No	Yes	Yes	No	3 studies; 2× same sample; follow-up; long-term effects unclear; dependent on individual factors; large sample	Moderate ⊕⊕⊕⊝
Knowledge acquisition[h] (critical)	$n = 59$ (RCT)[1]	1× moderate	No	No	Yes	No	Yes	No	No	Only 1 study; good applicability; transferable to other countries; low effort, low costs	Moderate ⊕⊕⊕⊝

[a]Assessment: randomized controlled trials: ROB 2; nonrandomized controlled trials: ROBINS-I; uncontrolled trials: adapted form of ROBINS-I; systematic reviews: AMSTAR-2.
[b]"Yes" if at least one publication fulfills this criterion.
[c]Some of the comments relate only to single publications.
[d]For systematic reviews, the number of participants and study designs of the relevant studies in the review are presented.
[e]Suboutcome of: improvement in the neuropsychological functions of children/adolescents with FASD.
[f]Suboutcome of: improving the participation of children/young people with FASD.
[g]Relief for caregivers (biological, foster and adoptive parents, caregivers) and improving the quality of life of the entire family/institution affected family/institution.
[h]Improving knowledge of the deviant state of health/disorder/disability and improvement of insight into the illness.
[i]Cannot be determined precisely due to overlaps in the study population.

 © 2025. Thieme. All rights reserved.

References for Supplementary Table D3.15

1. Kable JA, Coles CD, Strickland D, Taddeo E. Comparing the effectiveness of on-line versus in-person caregiver education and training for behavioral regulation in families of children with FASD. Int J Ment Health Addict 2012;10(6):791–803
2. Coles CD, Kable JA, Taddeo E, Strickland D. GoFAR: improving attention, behavior and adaptive functioning in children with fetal alcohol spectrum disorders: brief report. Dev Neurorehabil 2018;21(5):345–349
3. Petrenko CLM, Pandolfino ME, Robinson LK. Findings from the families on track intervention pilot trial for children with fetal alcohol spectrum disorders and their families. Alcohol Clin Exp Res 2017;41(7):1340–1351
4. Petrenko CLM, Demeusy EM, Alto ME. Six-month follow-up of the families on track intervention pilot trial for children with fetal alcohol spectrum disorders and their families. Alcohol Clin Exp Res 2019;43(10):2242–2254
5. Leenaars LS, Denys K, Henneveld D, Rasmussen C. The impact of fetal alcohol spectrum disorders on families: evaluation of a family intervention program. Community Ment Health J 2012;48(4):431–435

 © 2025. Thieme. All rights reserved.

Supplementary Table D3.16 Summary of findings, extrinsic reinforcements

Population: Children with prenatal alcohol exposure
Setting: Clinical setting
Intervention: Extrinsic reinforcements
Comparison: Children with ADHD, typically developed children, tasks without extrinsic reinforcements

Outcomes (relevance)	Number of participant (study design)[d]	Factors leading to quality downgrade					Factors leading to quality upgrade			Other factors to consider when making recommendations[c]	Certainty of the evidence—GRADE
		Risk of Bias[a]	Indirectness[b]	Inconsistency	Imprecision[b]	Publication bias[b]	Effect size[b]	Dose effect[b]	Residual plausible confounding[b]		
Attention[e] (critical)	$n = 88$ (control study)[1]	1× moderate	Yes	Yes	No	No	No	No	No	Only 1 study; low effort; no costs; good applicability; no negative consequences to be expected; no inactive comparison group; transferability to everyday life unclear	Low ⊕⊕⊝⊝

[a]Assessment: randomized controlled trials: ROB 2; nonrandomized controlled trials: ROBINS-I; uncontrolled trials: adapted form of ROBINS-I; systematic reviews: AMSTAR-2.
[b]"Yes" if at least one publication fulfills this criterion.
[c]Some of the comments relate only to single publications.
[d]For systematic reviews, the number of participants and study designs of the relevant studies in the review are presented.
[e]Suboutcome of: improvement in the neuropsychological functions of children/adolescents with FASD.

Reference for Supplementary Table D3.16

1. Graham DM, Glass L, Mattson SN. The influence of extrinsic reinforcement on children with heavy prenatal alcohol exposure. Alcohol Clin Exp Res 2016;40(2):348–358

 © 2025. Thieme. All rights reserved.

Supplementary Table D3.17 Summary of findings, trainings focusing on mental health

Population: Children with FASD/Autism spectrum disorders/Intellectual disability
Setting: School
Intervention: “The Brain Unit mental health literacy program“ and “Dialectical behavior therapy skill-building”
Comparison: Inactive comparison group

Outcomes (relevance)	Number of participant (study design)[d]	Factors leading to quality downgrade					Factors leading to quality upgrade			Other factors to consider when making recommendations[c]	Certainty of the evidence—GRADE
		Risk of Bias[a]	Indirectness[b]	Inconsistency	Imprecision[b]	Publication bias[b]	Effect size[b]	Dose effect[b]	Residual plausible confounding[b]		
Coping[e] (critical)	$n = 133$ (RCT)[1]	1× low	Yes	No	No	No	No	No	No	1 systematic review, only 1 study; large sample; feasibility unclear	Moderate ⊕⊕⊕⊖

[a]Assessment: randomized controlled trials: ROB 2; nonrandomized controlled trials: ROBINS-I; uncontrolled trials: adapted form of ROBINS-I; systematic reviews: AMSTAR-2.
[b]“Yes” if at least one publication fulfills this criterion.
[c]Some of the comments relate only to single publications.
[d]For systematic reviews, the number of participants and study designs of the relevant studies in the review are presented.
[e]Improvement in coping and self-efficacy.

Reference for Supplementary Table D3.17

1. Flannigan K, Coons-Harding KD, Anderson T, et al. A systematic review of interventions to improve mental health and substance use outcomes for individuals with prenatal alcohol exposure and fetal alcohol spectrum disorder. Alcohol Clin Exp Res 2020;44(12):2401–2430

 © 2025. Thieme. All rights reserved.

Supplementary Table D3.18 Summary of findings, animal-assisted therapies

Population: Children with FASD
Setting: Clinical setting
Intervention: Therapy program with therapy dogs
Comparison: Standard care

Outcomes (relevance)	Number of participant (study design)[d]	Factors leading to quality downgrade					Factors leading to quality upgrade			Other factors to consider when making recommendations[c]	Certainty of the evidence—GRADE
		Risk of Bias[a]	Indirectness[b]	Inconsistency	Imprecision[b]	Publication bias[b]	Effect size[b]	Dose effect[b]	Residual plausible confounding[b]		
Social skills and behavior[e] (critical)	$n=33$ (1× RCT)[1]	1× moderate	No	Yes	No	No	Yes	No	No	Only 1 study	Moderate ⊕⊕⊕⊝
Quality of life[f] (critical)	$n=33$ (1× RCT)[1]	1× moderate	Yes	Yes	No	No	No	No	No	Only 1 study; only severity of disease determined	Low ⊕⊕⊝⊝

[a]Assessment: randomized controlled trials: ROB 2; nonrandomized controlled trials: ROBINS-I; uncontrolled trials: adapted form of ROBINS-I; systematic reviews: AMSTAR-2.
[b]"Yes" if at least one publication fulfills this criterion.
[c]Some of the comments relate only to single publications.
[d]For systematic reviews, the number of participants and study designs of the relevant studies in the review are presented.
[e]Suboutcome of: improvement in the neuropsychological functions of children/adolescents with FASD.
[f]Improving the quality of life of children/adolescents with FASD.

Reference for Supplementary Table D3.18

1. Vidal R, Vidal L, Ristol F, et al. Dog-assisted therapy for children and adolescents with fetal alcohol spectrum disorders a randomized controlled pilot study. Front Psychol 2020;11:1080

 © 2025. Thieme. All rights reserved.
